# A Multicenter, Randomized, Placebo‐Controlled Trial of Atorvastatin for the Primary Prevention of Cardiovascular Events in Patients With Rheumatoid Arthritis

**DOI:** 10.1002/art.40892

**Published:** 2019-07-22

**Authors:** George D. Kitas, Peter Nightingale, Jane Armitage, Naveed Sattar, Jill J. F. Belch, Deborah P. M. Symmons, the TRACE RA Consortium, George Kitas, Jill Belch, Deborah Symmons, Hawys Williams, Shobna Vasishta, Rebecca Storey, Peter Nightingale, Ian Bruce, Paul Durrington, Iain McInnes, Naveed Sattar, Deva Situnayake, Allan Struthers, Gordon Lowe, Jane Armitage, Keith Fox, Dorian Haskard, Caroline Dore, Ailsa Bosworth, George Kitas, Jill Belch, Deborah Symmons, Hawys Williams, Michael Frenneaux, Christopher Edwards, Jonathan Emberson, Deborah Bax, Stuart Cobbe, David Stott, Roger Sturrock, Peter Macfarlane, Rainer Klocke, Tom Pullar, Susan Knight, Iain Rowe, Pradeep Kumar, Nicky Goodson, Diarmuid Mulherin, Micheal Brzeski, Philip Gardiner, Deva Situnayake, David Walker, Rob Callaghan, Margaret Allen, David McCarey, Emmanuel George, Chris Deighton, Bruce Kirkham, Lee‐Suan Teh, Raashid Luqmani, Kuntal Chakravarty, Jenny Nixon, Selwyn Richards, David Scott, Tony Woolf, Peter Prouse, Jonathan Packham, Martin Davies, Denise DeLord, Terence O’Neill, Ira Pande, John Harvie, Richard Watts, Elizabeth Rankin, George Papasavvas, Paul Emery, Arvind Sinha, Bhaskar Dasgupta, Ian Bruce, Paul Creamer, Asad Zoma, David Walsh, Jaap Van‐Laar, Nigel Capps, Andrew Cairns, Christopher Marguerie, Namita Kumar, Rikki Abernethy, Mark Lillicrap, Stuart Ralston, Raad Makadsi, Neil Hopkinson, Su Tan, Mohammed Akil, Yasmeen Ahmad, Matthew Adler, Marwan Bukhari, Paul Sanders, Euthalia Roussou, Khalid Binymin, Alaa Hassan, Rod Hughes, David O’Reilly, Paul Sainsbury, Ruth Richmond, Magliano Malgorzata, Mohammed Nisar, Ann McEntergart, Dipak Roy, Jeffrey Marks, Michael Batley, Frank McKenna, Mike Irani, Helen Harris, Anita Smyth, Eddie Tunn, Adam Young, Joegi Thomas, Frances Hall, Tarnya Marshall, Chandini Rao, Krishnan Baburaj, Josh Dixey, Nagui Gendi, Fraser Birrell, Gladstone Chelliah, Ann Morgan, Daniel Fishman, Sally Knights, David Coady, Raad Makadsi, Bill Smith, Beverley Harrison, David Walker, Stefan Siebert, Anthony Chan, Kiran Putchakayala, Atheer Al‐Ansari, Andrew Gough, Sophia Naz, Namita Kumar, Dev Pyne, Taher Mahmud, Yusaf Patel, Amanda Isdale

**Affiliations:** ^1^ Dudley Group NHS Foundation Trust, Russells Hall Hospital, Stourbridge, UK and Research UK Centre for Epidemiology Manchester UK; ^2^ University of Birmingham Birmingham UK; ^3^ University of Oxford Oxford UK; ^4^ University of Glasgow, Glasgow, UK and Oxford Centre for Diabetes, Endocrinology and Metabolism Oxford UK; ^5^ University of Dundee and Ninewells Hospital and Medical School Dundee UK; ^6^ Arthritis Research UK Centre for Epidemiology, University of Manchester and NIHR Manchester Biomedical Research Center Manchester NHS Foundation Trust Manchester UK

## Abstract

**Objective:**

Rheumatoid arthritis (RA) is associated with increased cardiovascular event (CVE) risk. The impact of statins in RA is not established. We assessed whether atorvastatin is superior to placebo for the primary prevention of CVEs in RA patients.

**Methods:**

A randomized, double‐blind, placebo‐controlled trial was designed to detect a 32% CVE risk reduction based on an estimated 1.6% per annum event rate with 80% power at *P* < 0.05. RA patients age >50 years or with a disease duration of >10 years who did not have clinical atherosclerosis, diabetes, or myopathy received atorvastatin 40 mg daily or matching placebo. The primary end point was a composite of cardiovascular death, myocardial infarction, stroke, transient ischemic attack, or any arterial revascularization. Secondary and tertiary end points included plasma lipids and safety.

**Results:**

A total of 3,002 patients (mean age 61 years; 74% female) were followed up for a median of 2.51 years (interquartile range [IQR] 1.90, 3.49 years) (7,827 patient‐years). The study was terminated early due to a lower than expected event rate (0.70% per annum). Of the 1,504 patients receiving atorvastatin, 24 (1.6%) experienced a primary end point, compared with 36 (2.4%) of the 1,498 receiving placebo (hazard ratio [HR] 0.66 [95% confidence interval (95% CI) 0.39, 1.11]; *P* = 0.115 and adjusted HR 0.60 [95% CI 0.32, 1.15]; *P* = 0.127). At trial end, patients receiving atorvastatin had a mean ± SD low‐density lipoprotein (LDL) cholesterol level 0.77 ± 0.04 mmoles/liter lower than those receiving placebo (*P* < 0.0001). C‐reactive protein level was also significantly lower in the atorvastatin group than the placebo group (median 2.59 mg/liter [IQR 0.94, 6.08] versus 3.60 mg/liter [IQR 1.47, 7.49]; *P* < 0.0001). CVE risk reduction per mmole/liter reduction in LDL cholesterol was 42% (95% CI −14%, 70%). The rates of adverse events in the atorvastatin group (n = 298 [19.8%]) and placebo group (n = 292 [19.5%]) were similar.

**Conclusion:**

Atorvastatin 40 mg daily is safe and results in a significantly greater reduction of LDL cholesterol level than placebo in patients with RA. The 34% CVE risk reduction is consistent with the Cholesterol Treatment Trialists’ Collaboration meta‐analysis of statin effects in other populations.

## Introduction

Despite major advances in therapy over the last two decades, rheumatoid arthritis (RA) continues to be associated with reduced life expectancy compared to the general population 1. Almost half of all deaths in RA (~35–40% of the excess deaths) are attributed to cardiovascular disease (CVD) 2. There are many mechanisms that may underlie the increased CVD morbidity and mortality in RA, but their cross‐talk and relative contributions are not yet fully elucidated. CVD risk factors, including smoking, hypertension, dyslipidemia, increased adiposity, and reduced physical activity, are highly prevalent in RA 3 but do not fully account for the excess CVD 4. A significant part is attributed to “novel” CVD risk factors, such as “high‐grade” inflammation promoting atherothrombotic cardiovascular events (CVEs) 4, 5. Risk algorithms developed for the general population may underestimate CVE risk in patients with RA 6, 7, 8, even when multipliers are applied, as in recently updated European League Against Rheumatism recommendations 9. This makes identification of RA patients who would benefit from primary prevention therapy less precise, leads to significant underuse of statins even in patients who fulfill general population thresholds for statin treatment 10, and has led some to suggest universal prescription of statins in RA 11, as practiced in diabetes mellitus (DM).

The efficacy of statins in the primary and secondary prevention of CVEs has been demonstrated in large‐scale trials and meta‐analyses 12. CVE reduction is related to the degree of reduction of low‐density lipoprotein (LDL) cholesterol levels. Each millimole per liter reduction in LDL cholesterol is associated with a 20–22% reduction in the risk of myocardial infarction (MI), revascularization, and stroke 12. In RA, high‐grade inflammation is associated with suppression of total cholesterol, LDL cholesterol, and high‐density lipoprotein (HDL) cholesterol levels, as well as changes in lipid structure and function, promoting atherosclerosis 13, 14. The potential pleiotropic antiinflammatory/immunomodulatory effects of statins 15 may therefore be more relevant in RA than in the general population. In the Trial of Atorvastatin in Rheumatoid Arthritis (TARA), atorvastatin 40 mg daily, as an adjunct to disease‐modifying antirheumatic drug (DMARD) therapy, provided a modest additional benefit for control of inflammation in RA, at least in a subgroup of patients 16, while the Tayside controlled study of rosuvastatin in RA suggested a potentially beneficial effect on C‐reactive protein (CRP) levels 17. The extent to which statins affect lipid levels and reduce CVEs in RA remains uncertain, due to the small number of RA patients included in general population trials 18.

The lack of robust primary prevention data, coupled with the multifaceted pharmacologic potential of statins in RA suspected at that time, prompted the Trial of Atorvastatin for the Primary Prevention of Cardiovascular Events in Patients with Rheumatoid Arthritis (TRACE RA), the only statin trial with hard CVE end points in this population.

## Patients and methods

#### Study design

TRACE RA was a multicenter, randomized, double‐blind, placebo‐controlled trial comparing atorvastatin 40 mg once daily (supplied by Pfizer UK) with placebo (dummy atorvastatin) for the primary prevention of CVEs in patients with RA. The trial was conducted in 102 rheumatology units in the UK, approved by the Southampton and South West Hampshire Multicentre Research Ethics Committee (Ref. No 06/Q1704/171), and registered with International Standard Randomised Controlled Trial Number 41829447. The final protocol is available at https://www.staffnet.manchester.ac.uk/rbe/ethics-integrity/clinical-trials/portfolio/tracera/ and in Supplementary Methods 1, available on the *Arthritis & Rheumatology* web site at http://onlinelibrary.wiley.com/doi/10.1002/art.40892/abstract. See Appendix A for study centers and members of the TRACE RA Consortium.

#### Participants

Patients were eligible if they fulfilled the American College of Rheumatology 1987 criteria for RA 19, were >50 years of age or had an RA disease duration of >10 years, and gave informed consent. Patients taking statins and those with known CVD requiring statins, DM, myopathy, or other contraindications to statins were excluded (see Supplementary Methods 1). Recruiting centers continued their routine practice for screening (or not) for cardiovascular risk. There were no restrictions with regard to RA treatment prior to or during the trial period, other than the requirement that patients receive stable doses of antirheumatic medication for the 3 months prior to inclusion in the study. Potentially eligible patients were identified during routine clinical visits, given the patient information sheet, and invited to contact the local trial team if they were interested in participating. A screening visit was then arranged. All patients recruited provided written informed consent.

#### Randomization and masking

Trial medication was provided by Pfizer UK, bottled by an independent pharmaceutical company (Catalent Pharma Solutions UK) to good manufacturing practice standards, and dispensed by the local study pharmacist. The randomization process was incorporated into the drug labeling. Center was the only stratifying variable. Catalent performed the randomization, labeled each bottle with a unique number, and supplied the packaged drugs to hospital pharmacies with scratch cards to allow a patient's treatment allocation to be revealed, if necessary. On entering the trial, each patient was given a filled and labeled bottle, coded with a unique study number, which was used for all future supplies for that patient. Study treatment remained double‐blind for patients, investigators, and study personnel throughout.

#### Procedures

The trial comprised 3 stages: 1) a screening visit to confirm patient eligibility, secure consent, counsel (verbally and with a leaflet) patients on modifiable cardiovascular risk factors, and randomize patients (there was no run‐in period); 2) a 3‐month visit to check drug tolerability and safety (by liver function tests and creatine kinase [CK] level); and 3) an intended minimum 5‐year treatment period. At the screening visit, baseline information on demographic characteristics, medical history, family history of premature CVD, smoking status, and concomitant medication was obtained through interview and case note review. The presence of hypertension was assessed by the case report form question, “Is the patient known to have hypertension?” Height, weight, and waist circumference were measured. RA disease activity, severity (physical function), and quality of life were assessed using the Disease Activity Score in 28 joints (DAS28) 20, the UK version of the Health Assessment Questionnaire (HAQ) 21 disability index (DI), and the EuroQol 5‐domain (EQ‐5D) instrument 22, respectively. Blood samples were collected for routine measurements of hematologic features, biochemical features, erythrocyte sedimentation rate, CRP, rheumatoid factor, and/or anti–cyclic citrullinated peptide antibodies.

The protocol did not require measurement of lipid levels at baseline. The results of any lipid measurements that had been requested routinely in primary or secondary care over the previous 12 months were recorded in the clinical trial record. If there was more than 1 lipid measurement, the most recent was used. General practitioners (GPs) were informed if patients were found to have hypertension, DM, or an existing indication for statins (e.g., known hyperlipidemia, DM, or previously known high CVD risk [according to standard guidelines] requiring a statin for primary or secondary prevention).

Randomized patients were followed up at 3 and 6 months and every 6 months thereafter in person or by telephone. Information on trial efficacy and safety end points, disease activity, severity, and concomitant medication was collected at each visit. Patients were asked if they had taken “most,” “some,” or “none” of their tablets. Patients were considered compliant if they reported taking “most” of their study tablets since their last visit. Study drug administration could be paused, if necessary, for up to 4 weeks without violating the protocol. Patients who experienced a validated primary end point had no further trial visits but were followed up for mortality via linkage with national death registers. Secondary prevention in these patients was decided by the GP and/or treating physician. Patients who were withdrawn from the study for reasons other than a primary end point continued to attend follow‐up visits to facilitate adverse event and clinical end point data collection. Patients who developed a clinical need for a statin, other than a primary end point, after randomization could be prescribed up to 40 mg of atorvastatin in addition to the randomized trial medication and remain in the trial.

#### Outcome measures

The prespecified primary end point was “major vascular events,” defined as nonfatal MI, nonfatal presumed ischemic stroke, transient ischemic attack (TIA), any coronary or non‐coronary revascularization, or cardiovascular death, excluding both confirmed cerebral hemorrhage (International Statistical Classification of Diseases and Related Health Problems, Tenth Revision [ICD‐10] codes I64–I99) 23 and non‐coronary cardiac death (ICD‐10 codes I00–I15 and I26–I52), occurring during the scheduled treatment period. Secondary end points were the separate components of the primary end point. Tertiary end points included total and cause‐specific mortality (coronary, other vascular, and nonvascular death separately); hospitalizations; statin safety‐related outcomes (persistent elevation of alanine transaminase [ALT] or aspartate transaminase [AST] or myopathy [muscle symptoms plus CK >10× the upper limit of normal (ULN)]; and between‐group differences at study end in lipid levels and health‐related outcomes (physical function and quality of life).

Additional information about all potential primary end points was collected from medical records, death certificates, and postmortem examinations (where available). An independent trial end points committee reviewed such information on all potential CVEs and deaths and classified them according to prespecified criteria (see Supplementary Methods 1). Information about hospital admissions was ascertained via linkage, using each patient's unique National Health Service number, with the Health and Social Care Information Centre (HSCIC) for England and Wales and the Scottish Office's Information and Statistics Division (ISD), and the local hospital medical records departments. Information on mortality and cause of death was obtained via linkage with the HSCIC and ISD. Patients were asked at each visit about adverse events including muscle pain, and ALT, AST, and CK were measured at 3 months. Liver function tests were also performed regularly (usually every 2–3 months) as part of routine DMARD monitoring. At the final study visit, patients were asked to provide a blood sample for lipid and CRP analysis. These samples were shipped to a single laboratory and measured, blinded with regard to treatment group, on an automated validated platform (c311; Roche Diagnostics) using the manufacturer's calibrators and quality control material. Between‐run coefficients of variation were all <5.2%. LDL cholesterol level was estimated using the Friedewald equation 24.

#### Statistical analysis

The original protocol (Supplementary Methods 2, available on the *Arthritis & Rheumatology* web site at http://onlinelibrary.wiley.com/doi/10.1002/art.40892/abstract) anticipated that a trial of 3,800 patients followed up for 5 years would have sufficient statistical power to detect plausible risk reduction with atorvastatin. However, a lower than expected event rate led to a protocol amendment (Supplementary Methods 3, available on the *Arthritis & Rheumatology* web site at http://onlinelibrary.wiley.com/doi/10.1002/art.40892/abstract). The final protocol specified a sample size of 5,400, which would have had 80% power to detect a 32% relative risk reduction in the primary end point in the atorvastatin versus placebo arms based on 434 primary events (Supplementary Methods 1 and Supplementary Figure 1, available on the *Arthritis & Rheumatology* web site at http://onlinelibrary.wiley.com/doi/10.1002/art.40892/abstract). However, the ongoing much lower‐than‐anticipated CVE rate led to premature termination of the trial.

All randomized patients were included in the analysis up to December 20, 2012 or the end of trial visit, whichever was earlier, irrespective of whether the study drug was continued (i.e., intent‐to‐treat analyses). Cox regression models were developed for time to occurrence of a first CVE using treatment allocation as the independent variable. The models were stratified by center and adjusted for baseline imbalances, compliance, and nonstudy statin use (the latter two as time‐dependent variables using a previously described method 25). All adjustments were prespecified in the protocol and the statistical analysis plan (Supplementary Methods 4, available on the *Arthritis & Rheumatology* web site at http://onlinelibrary.wiley.com/doi/10.1002/art.40892/abstract). Treatment differences were expressed as hazard ratios (HRs) with 95% confidence intervals (95% CIs). *P* values less than 0.05 (2‐sided) were considered significant. Kaplan‐Meier product‐limit estimates of the survival curves were calculated. Lipid levels and levels of blood tests monitoring statin safety were compared between groups using Mann‐Whitney tests. All analyses were performed with SPSS Statistics for Windows, version 22.0 (IBM).

## Results

#### Trial progress

**Figure 1:**
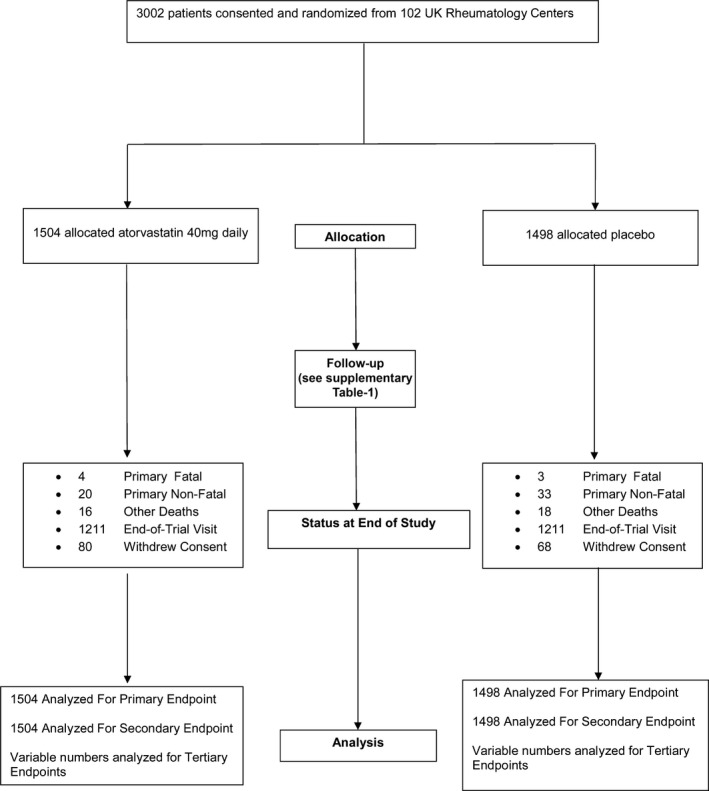
Trial profile. A total of 3,002 patients from 102 UK rheumatology centers were randomized in the Trial of Atorvastatin for the Primary Prevention of Cardiovascular Events in Patients with Rheumatoid Arthritis (TRACE RA). Of those, 1,504 were randomized to receive atorvastatin 40 mg daily and 1,498 were randomized to receive matching placebo. A detailed breakdown of follow‐up during the course of the trial is shown in Supplementary Table 1, available on the *Arthritis & Rheumatology* web site at http://onlinelibrary.wiley.com/doi/10.1002/art.40892/abstract. All randomized patients were included in the intent‐to‐treat analysis for the primary and secondary end points. Variable numbers of patients, based on data availability, were used for analyses of other outcomes.

Between August 7, 2007 and November 21, 2011, 3,002 patients with RA from 102 centers were randomized (1,504 to receive atorvastatin and 1,498 to receive placebo). Their mean age was 61 years (228 [7.6%] of 3,002 patients were <50 years of age), and 74% were women. They were followed up for a median of 2.51 years (interquartile range [IQR] 1.90, 3.49 years), providing 7,827 person‐years of follow‐up. At the time of trial closure (December 31, 2011), the observed event rate in the 2 arms combined was 0.70% per annum compared with the expected 1.6% per annum. Trial progress is shown in Figure 1 and Supplementary Table 1, available on the *Arthritis & Rheumatology* web site at http://onlinelibrary.wiley.com/doi/10.1002/art.40892/abstract.

#### Baseline characteristics of the patients

At baseline, the randomized groups were well balanced for all demographic, anthropometric, and RA characteristics, antirheumatic and other therapies, and CVD risk factors, except for current smoking and nonsteroidal antiinflammatory drug (NSAID) or cyclooxygenase 2 inhibitor (coxib) treatment, which were higher in the atorvastatin group than in the placebo group (18.4% versus 14.5% for current smoking and 42.9% versus 38.0% for NSAID or coxib treatment). A total of 40.3% of the patients had low disease activity according to the DAS28 or were in remission (DAS28 ≤3.2), 86% were receiving stable doses of conventional synthetic DMARDs, 16% were receiving biologic DMARDs, and 17% were receiving steroid therapy (Table 1 and Supplementary Table 2, available on the *Arthritis & Rheumatology* web site at http://onlinelibrary.wiley.com/doi/10.1002/art.40892/abstract).

**Table 1:** Baseline characteristics of the patients randomized to receive atorvastatin or placebo^a^

	Atorvastatin 40 mg (n = 1,504)	Placebo (n = 1,498)
Demographic/anthropometric characteristics		
Sex, female	1,107/1,504 (74)	1,120/1,498 (75)
Age, mean ± SD years (n)	61.1 ± 8.3 (1,500)	60.9 (8.5) (1491)
Race, white	1,394/1,421 (98)	1,407/1,430 (98)
BMI, median (IQR) (n)	26.4 (23.7, 30.1) (1,466)	26.8 (24.0, 30.1) (1,432)
RA characteristics		
Time since symptom onset, median (IQR) years (n)	13 (6, 21) (1,471)	13 (6, 21) (1,460)
Time since diagnosis, median (IQR) years (n)	11 (4, 18) (1,499)	11 (5, 20) (1,489)
RF and/or ACPA positive	737/1,177 (63)	709/1,153 (62)
DAS28, median (IQR) (n)	3.7 (2.6, 4.7) (1,471)	3.5 (2.5, 4.6) (1,471)
HAQ DI score, median (IQR) (n)	1.25 (0.50, 1.88) (1,473)	1.25 (0.38, 1.88) (1,464)
EQ‐5D, median (IQR) (n)	0.62 (0.52, 0.80) (1,422)	0.689 (0.52, 0.80) (1,408)
Treatment		
Biologic DMARDs	229/1,466 (16)	232/1,458 (16)
Conventional synthetic DMARDs	1,264/1,466 (86)	1,241/1,458 (85)
Steroids	253/1,466 (17)	241/1,458 (17)
NSAIDs/coxibs	629/1,466 (43)	554/1,458 (38)
Cardiovascular characteristics		
Smoking status		
Current smoker	260/1,422 (18)	209/1,431 (15)
Ex‐smoker	606/1,422 (43)	637/1,431 (45)
Never smoked	556/1,422 (39)	585/1,431 (41)
Hypertension	322/1,456 (22)	335/1,437 (23)
First degree relative with premature CVD	285/1,321 (22)	263/1,304 (20)
Total cholesterol, median (IQR) mmoles/liter (n)	5.4 (4.8, 6.1) (845)	5.3 (4.8, 6.0) (832)
Triglycerides, median (IQR) mmoles/liter (n)	1.26 (0.90, 1.80) (673)	1.30 (0.90, 1.80) (652)
HDL cholesterol, median (IQR) mmoles/liter (n)	1.56 (1.2, 1.90) (719)	1.52 (1.25, 1.85) (700)
LDL cholesterol, median (IQR) mmoles/liter (n)	3.2 (2.7, 3.8) (544)	3.2 (2.7, 3.8) (530)
CRP, median (IQR) mg/liter (n)	5 (3, 11) (780)	5 (3, 12) (776)
Estimated GFR, median (IQR) ml/minute/1.73 m^2^ (n)	79 (59, 110) (1,124)	79 (58, 111) (1,109)
Treatment		
Aspirin	3/116 (3)	3/126 (2)
ACE inhibitors	10/113 (9)	10/127 (8)
Other cardiac drugs	10/113 (9)	10/123 (8)

a The variable number of patients for each characteristic is due to missing data from incomplete case report forms. The low number of baseline lipid measurements is because, due to budgetary constraints, the protocol did not require measurement of lipid levels at baseline. In the UK, it is the responsibility of primary care physicians to assess their patients for cardiovascular risk and to prescribe statins for primary prevention where indicated according to national guidelines. The Trial of Atorvastatin for the Primary Prevention of Cardiovascular Events in Patients with Rheumatoid Arthritis (TRACE RA) aimed to recruit patients who did not have cardiovascular disease (CVD) at baseline and who were not already taking a statin for primary prevention. If lipid levels had been measured routinely in the 12 months prior to recruitment (in primary or secondary care), the results were recorded in the trial case report form. Except where indicated otherwise, values are the number of patients/number for whom data were available (%). BMI = body mass index; IQR = interquartile range; RA = rheumatoid arthritis; RF = rheumatoid factor; ACPA = anti–citrullinated protein antibody; DAS28 = Disease Activity Score in 28 joints; HAQ DI = Health Assessment Questionnaire disability index; EQ‐5D = EuroQol 5‐domain; DMARDs = disease‐modifying antirheumatic drugs; NSAIDs = nonsteroidal antiinflammatory drugs; HDL = high‐density lipoprotein; LDL = low‐density lipoprotein; CRP = C‐reactive protein; GFR = glomerular filtration rate; ACE = angiotensin‐converting enzyme.

#### Compliance and nonstudy statin use

In the atorvastatin group, reported compliance fell from 89% at the 3‐month visit to 39% by 60 months of follow‐up, while nonstudy statin use increased from 0.5% to 5.6%. In the placebo group, compliance fell from 89% to 25% and nonstudy statin use increased from 0.7% to 7.8% (Supplementary Table 3, available on the *Arthritis & Rheumatology* web site at http://onlinelibrary.wiley.com/doi/10.1002/art.40892/abstract). Time‐weighted average compliance was 66% in the atorvastatin arm and 65% in the placebo arm. Time‐weighted nonstudy statin use was 1.6% in the atorvastatin arm and 3.3% in the placebo arm.

#### Primary end point

**Figure 2:**
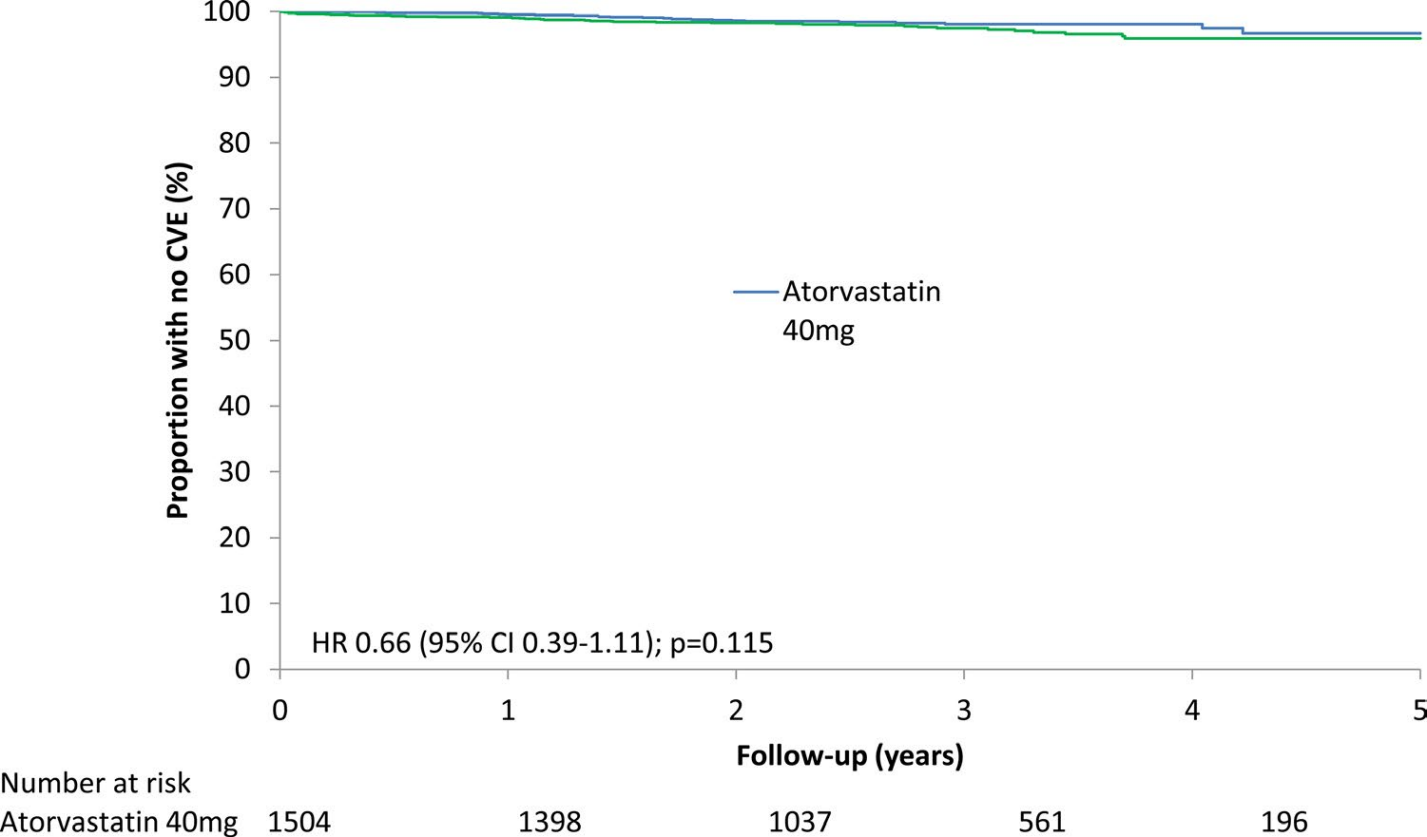
Kaplan‐Meier plots of time to first cardiovascular event (CVE) for patients in the atorvastatin and placebo groups. HR = hazard ratio; 95% CI = 95% confidence interval.

**Figure 3:**
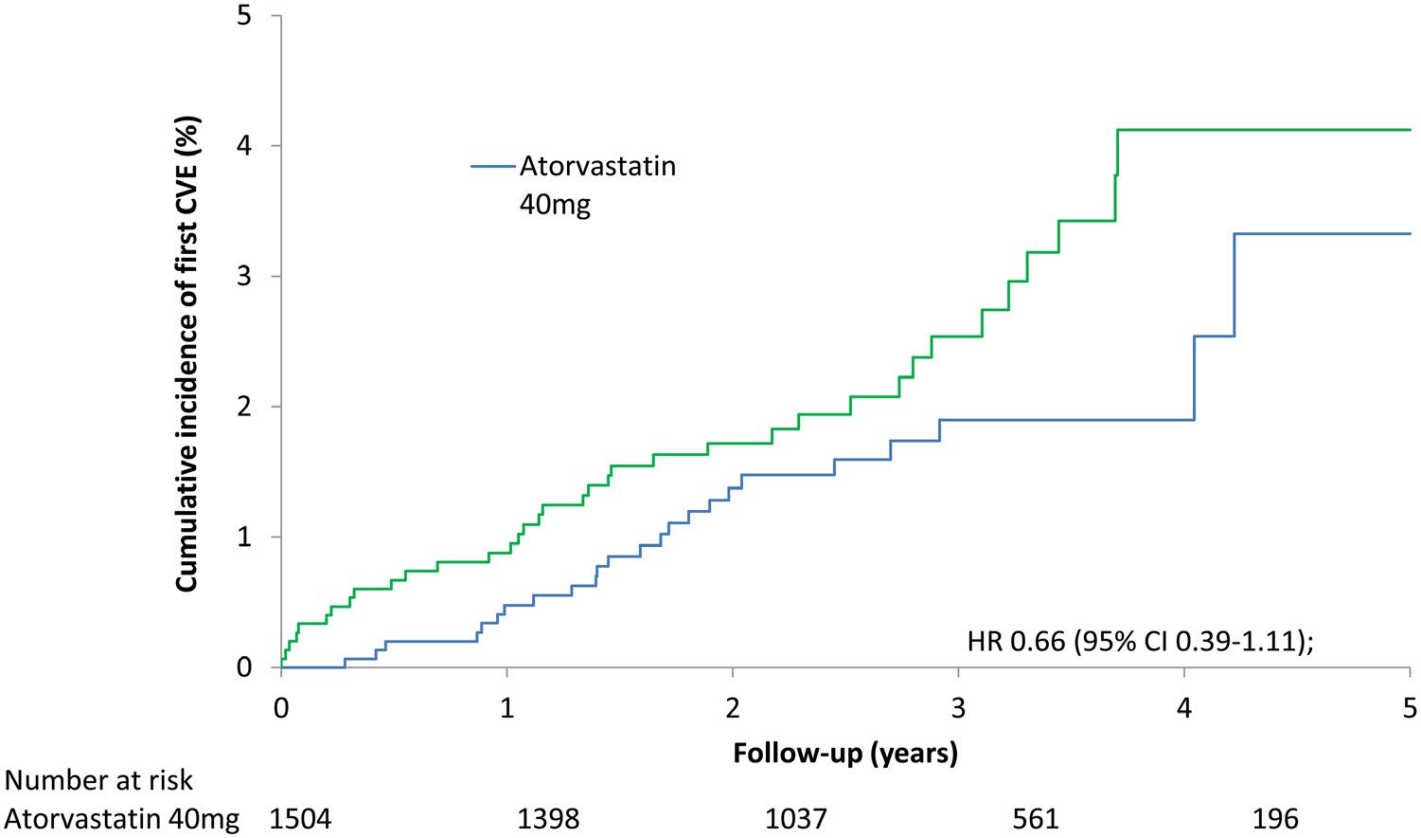
Cumulative incidence of first cardiovascular event (CVE) for patients in the atorvastatin and placebo groups. HR = hazard ratio; 95% CI = 95% confidence interval.

Twenty‐four patients allocated to receive atorvastatin (1.6%) had a confirmed CVE, compared to 36 (2.4%) of the patients allocated to receive placebo (HR 0.66 [95% CI 0.39, 1.11]; *P* = 0.115). After adjustment for baseline differences, compliance, and nonstudy statin use, the HR was 0.60 (95% CI 0.32, 1.15) (*P* = 0.127). Based on the number of events, the numbers of patients, and the mean follow‐up time in each arm, the number needed to treat to prevent 1 CVE during the trial was 121. Kaplan‐Meier analysis of time to primary end point in the 2 groups is shown in Figure 2, and the cumulative incidence of first CVE in the 2 groups is shown in Figure 3. The estimated reduction in CVE risk per 1 mmole/liter reduction in LDL cholesterol level was 42% (95% CI −14%, 70%) (Supplementary Figure 2, available on the *Arthritis & Rheumatology* web site at http://onlinelibrary.wiley.com/doi/10.1002/art.40892/abstract). This was calculated by extrapolating the HR of 0.66 for a 0.77 mmoles/liter reduction to an HR of 0.66 to the power of (1/0.77) for a 1 mmole/liter reduction, i.e., an HR of 0.58 or a CVE risk reduction of 42%.

#### Secondary end points

##### Individual components of the primary end point

Coronary events (nonfatal MI, coronary death, or coronary revascularization) occurred in 13 (0.9%) of the patients in the atorvastatin group versus 23 (1.5%) of the patients in the placebo group. Presumed ischemic stroke or TIA occurred in 6 (0.4%) of the patients in the atorvastatin group versus 12 (0.8%) of the patients in the placebo group, and any non‐coronary arterial revascularization occurred in 3 (0.2%) of the patients in the atorvastatin group versus 1 (0.1%) of the patients in the placebo group. No other cardiovascular death occurred in either group. A peripheral atherosclerotic event occurred in 1 (0.1%) of the patients in the atorvastatin group and none of the patients in the placebo group, and suspected coronary heart disease death occurred in 2 (0.1%) of the patients in the atorvastatin group versus 1 (0.1%) of the patients in the placebo group (Supplementary Table 4, available on the *Arthritis & Rheumatology* web site at http://onlinelibrary.wiley.com/doi/10.1002/art.40892/abstract).

##### Total and cause‐specific mortality

Total and cause‐specific mortality (coronary, other vascular, and nonvascular deaths separately) did not differ between the 2 arms (25 deaths in the atorvastatin arm [1.7%] and 27 deaths in the placebo arm [1.8%]) (Supplementary Table 4).

#### Safety outcomes

There were no suspected unexpected serious adverse reactions. There were 298 reported adverse events in the atorvastatin arm (19.8%) and 292 in the placebo arm (19.5%) (*P* = 0.854) (Table 2).

**Table 2:** Adverse events according to ICD‐10 chapter by treatment arm^a^

	Atorvastatin 40 mg (n = 1,504)	Placebo (n = 1,498)
Infectious and parasitic disease	16 (1.1)	15 (1.0)
Neoplasms	28 (1.9)	30 (2.0)
Blood and blood‐forming organs and immune system disease	5 (0.3)	2 (0.1)
Endocrine, nutritional, and metabolic disease	1 (0.1)	1 (0.1)
Mental and behavioral disorder	2 (0.1)	1 (0.1)
Nervous system	4 (0.3)	10 (0.7)
Eye and adnexa	8 (0.5)	5 (0.3)
Ear and mastoid disease	2 (0.1)	0 (0.0)
Circulatory disease	40 (2.7)	45 (3.0)
Respiratory disease	33 (2.2)	38 (2.5)
Digestive system disease	37 (2.5)	28 (1.9)
Skin and subcutaneous system disease	12 (0.8)	8 (0.5)
Musculoskeletal and connective tissue disease	20 (1.3)	22 (1.5)
Genitourinary system disease	13 (0.9)	11 (0.7)
Symptoms, signs, and abnormal clinical and laboratory findings not classified elsewhere	8 (0.5)	10 (0.7)
Injury, poisoning	18 (1.2)	16 (1.1)
External causes of morbidity and mortality	23 (1.5)	19 (1.3)
None	111 (7.4)	97 (6.5)
Missing	14 (0.9)	14 (0.9)
Any adverse event	298 (19.8)	292 (19.5)

a Values are the number (%) of patients. ICD‐10 = International Statistical Classification of Diseases and Related Health Problems, Tenth Revision.

Two hundred fourteen (14.2%) of the patients in the atorvastatin group versus 223 (14.9%) of the patients in the placebo group had ≥1 hospitalization, with an identical median stay of 3 days (IQR 1, 6 days). There were no differences in the number of hospitalizations per patient (*P* = 0.710 by Kendall's tau‐b) or in the proportion of patients with ≥1 hospitalization (*P* = 0.641 by Fisher's exact test) (Supplementary Table 5, available on the *Arthritis & Rheumatology* web site at http://onlinelibrary.wiley.com/doi/10.1002/art.40892/abstract).

Life‐threatening but nonfatal serious adverse events (SAEs) occurred in 22 patients in the atorvastatin group versus 24 patients in the placebo group, while SAEs resulting in death occurred in 19 and 18 patients, respectively. None were considered to be related to trial medication. The randomization code was broken at the site in 3 cases in the atorvastatin arm (due to liver cysts with elevated ALT level, acute hepatitis, and abnormal findings on liver function tests) and 2 cases in the placebo arm (due to high grade lymphoma and chest infection), none of which was attributed to trial medication.

There were 64 reports of “RA flare” (significant worsening of RA symptoms), 29 in the atorvastatin group and 35 in the placebo group. There were 249 reports of “new or significant muscle pain,” 132 in the atorvastatin group versus 117 in the placebo group (*P* = 0.354). Of these, 13 (9 patients in the atorvastatin group versus 4 patients in the placebo group) had concurrent ALT or AST elevation of >2× the ULN, which was neither sustained nor considered to be related to the trial medication. Three of these patients (2 in the atorvastatin group and 1 in the placebo group) were withdrawn from the trial by the local principal investigator. None of the patients had a CK elevation of >10× the ULN. Two patients (1 in each group) had unsustained CK elevations of 3–10× the ULN; neither was considered to be due to trial medication. No asymptomatic cases of CK elevation were detected either on monitoring per protocol or during routine DMARD monitoring. There were no cases of ALT or AST elevation of >5× the ULN on per protocol testing, but there were 6 cases outside protocol testing (all unsustained and considered unrelated to trial medication). Overall, there were 159 cases of ALT or AST elevation of 2–5× the ULN (90 in the atorvastatin group and 69 in the placebo group; *P* = 0.103), none of which was sustained or attributed to trial medication.

#### Biochemical and arthritis outcomes at the end of the trial

At the end of the trial, mean LDL cholesterol levels were 0.77 mmoles/liter lower among those allocated to receive atorvastatin compared to those allocated to receive placebo. In the atorvastatin group, 54% of the patients were classified as compliant at the end‐of‐trial visit. There were no significant differences between groups in RA disease activity (DAS28), severity (HAQ DI), or quality of life (EQ‐5D). However, CRP levels were significantly lower in the atorvastatin group (median 2.59 mg/liter [IQR 0.94, 6.08]) than in the placebo group (median 3.60 mg/liter [IQR 1.47, 7.49]) (*P* < 0.0001). Although levels of CK and ALT (but not AST) were statistically significantly higher (by ~12–15%) in the atorvastatin group (Table 3), these differences are not considered clinically significant. The number of cases of myopathy and elevations of liver enzyme levels above the normal range were similar in the 2 groups. In the end‐of‐trial analysis of the atorvastatin group, lipid, ALT, and CRP levels were significantly associated with compliance (data not shown).

**Table 3:** Lipid levels and other outcomes at trial end by treatment arm^a^

	Atorvastatin 40 mg (n = 1,504)	Placebo (n = 1,498)	Difference (atorvastatin minus placebo)	*P* b
Lipid variable, mean ± SEM (n)				
Total cholesterol, mmoles/liter	4.13 ± 0.04 (987)	4.86 ± 0.04 (973)	−0.72 ± 0.05	<0.0001
Triglycerides, mmoles/liter	1.10 ± 0.02 (987)	1.26 ± 0.03 (973)	−0.16 ± 0.03	<0.0001
HDL cholesterol, mmoles/liter	1.41 ± 0.01 (987)	1.30 ± 0.01 (972)	0.11 ± 0.02	<0.0001
LDL cholesterol, mmoles/liter	2.21 ± 0.03 (985)	2.98 ± 0.03 (965)	−0.77 ± 0.04	<0.0001
Other variables, median (IQR) (n)				
CK, units/liter	94 (69, 135) (986)	84 (60, 118) (971)	–	<0.0001
CRP, mg/liter	2.59 (0.94, 6.08) (987)	3.60 (1.47, 7.49) (972)	–	<0.0001
ALT, units/liter	24.0 (17.4, 33.0) (987)	20.8 (15.5, 27.7) (973)	–	<0.0001
AST, units/liter	36.2 (28.5, 46.7) (987)	35.6 (27.5, 46.6) (973)	–	0.185
Clinical outcomes, median (IQR) (n)				
EQ‐5D score	0.66 (0.52, 0.80) (1,062)	0.70 (0.52, 0.80) (1,079)	–	0.301
HAQ DI score	1.25 (0.38, 1.88) (1,105)	1.25 (0.38, 1.97) (1,124)	–	0.644
DAS28 score	3.3 (2.3, 4.4) (997)	3.3 (2.4, 4.4) (1,023)	–	0.515
DAS28 category, no./no. available (%)				0.368
High (>5.1)	133/997 (13.3)	129/1,023 (12.6)	–	
Moderate (>3.2, ≤5.1)	391/997 (39.2)	428/1,023 (41.8)	–	
Low (>2.6, ≤3.2)	153/997 (15.3)	171/1,023 (16.7)	–	
Remission (≤2.6)	320/997 (32.1)	295/1,023 (28.8)	–	

a All patients attending the end‐of‐trial visit (1,211 per arm) were invited to provide blood samples for measurement of lipid levels and other variables. Of these patients, ~83% in each study arm agreed. HDL = high‐density lipoprotein; LDL = low‐density lipoprotein; IQR = interquartile range; CK = creatine kinase; CRP = C‐reactive protein; ALT = alanine transaminase; AST = aspartate transaminase; EQ‐5D = EuroQol 5‐domain; HAQ DI = Health Assessment Questionnaire disability index.

b By *t*‐test for lipid levels, by Kendall's tau‐b for Disease Activity Score in 28 joints (DAS28) category, and by Mann‐Whitney tests for all other comparisons.

## Discussion

TRACE RA was designed to assess whether patients with RA who were not already receiving statin therapy would benefit from atorvastatin 40 mg daily for the primary prevention of CVEs. In this study, the largest ever academically‐led clinical trial in RA, >3,000 RA patients were recruited and followed up for a median of 2.5 years. The unexpectedly low event rate and resulting limited statistical power to detect an effect during the planned 5 years of follow up led to premature termination of the trial. The best estimate of the “true” reduction in CVEs in the atorvastatin versus placebo arm is 34%. Using a 95% confidence level we cannot rule out any effect size between a 61% reduction and an 11% increase. Thus, our results were not statistically significant. The observed 34% reduction is consistent with the Cholesterol Treatment Trialists’ Collaboration meta‐analysis of the effect of statins in other populations 12. Furthermore, in this potentially vulnerable population, atorvastatin was safe, with no excess reports of muscle pain or other significant symptoms among those allocated atorvastatin compared to those receiving placebo.

There were several reasons such a trial was needed. CVD remains a major cause of death 5 and is significantly increased in people with RA compared to the general population 26, a fact recognized by the addition of RA as an independent risk factor in CVD risk algorithms such as QRISK2 27 and QRISK3 28. The relative contribution of classic CVD risk factors and novel mechanisms related to systemic inflammation to the excess CVD mortality of RA is still debated 4, 29, 30, and there have been no clinical end point trials assessing the effect of statins, or any other primary prevention strategy, in this population. Some small studies have shown that statins reduce surrogate measures of atherosclerotic events, for example, arterial stiffness 31 or carotid plaque 32, while a few cohort studies have suggested that statin use is associated with survival gains 33 and statin discontinuation with poorer survival 34 in RA. Finally, post hoc analyses of two trials of more intensive versus standard statin doses have suggested that the effect of statins, in terms of both LDL cholesterol reduction and CVE prevention, is similar in subjects with “inflammatory joint disease,” including RA, and those without joint inflammation; however these findings were based on a very small number of patients and events 18.

Randomization in TRACE RA was stratified only by study site in the expectation that, given the large numbers, baseline variables would distribute evenly between the treatment arms. However, baseline current smoking and NSAID/coxib usage, both well‐established risk factors for CVEs 35, 36, were higher in the atorvastatin group. Although every effort was made to maximize adherence to the trial medication during the trial, adherence rates in TRACE RA were relatively low. Adherence to trial medication in statin trials varies widely 37. This appears to depend on many factors, including the population studied, whether it is for primary or secondary prevention, trial design (e.g. inclusion of a “run‐in” period), trial duration, and method of assessing adherence, among others. Adherence to statin treatment in real‐world use is generally accepted to be <50% 37, 38. In this context, the adherence observed in TRACE RA, although disappointing, is probably not particularly poor. Prespecified adjusted analyses for baseline differences, compliance, and nonstudy statin use resulted in an HR of 0.60 (95% CI 0.32, 1.15) (*P* = 0.127).

From a clinical perspective, the safety outcomes are as important as CVE reduction. RA patients typically have multiple comorbidities 39 and polypharmacy 40, often with potentially hepatotoxic drugs. Virtually all participants in TRACE RA were receiving potentially hepatotoxic therapies such as methotrexate, but all patients receiving methotrexate were also prescribed folic acid. The 40 mg daily dose of atorvastatin is also of interest, as there are few randomized data on safety for this dose. Reassuringly, the type and severity of adverse events, the rate of hospitalizations, elevations of liver or muscle enzyme levels, incidence of myalgia, and worsening of RA were all similar in the 2 arms. These results suggest that atorvastatin 40 mg (and lower doses) is safe to use in patients with RA who are already receiving DMARD therapy.

Clinically assessed RA disease activity, severity, and quality of life were not significantly different between the 2 groups at the end of the trial. However, consistent with data from other studies 16, 17, levels of CRP were significantly lower, by ~1 mg/liter, in the atorvastatin group than in the placebo group. This difference is unlikely to be clinically significant in the context of RA disease activity.

Since TRACE RA was terminated early because the CVE rate was much lower than expected, it is not surprising that the HR for the primary end point was not significant. The observed number of primary outcomes provides <20% power to detect the relative risk reduction of 32% specified in the final protocol and provides adequate power (>80%) only to detect a relative risk reduction of >68%. The results for the primary outcome are therefore best represented as the estimated HR and its associated confidence interval. When the trial was designed (2002–2004), the assumption of a 1.6–1.8% annual event rate seemed, if anything, conservative. A meta‐analysis of mortality studies in RA published prior to 2005 demonstrated a meta–standardized mortality ratio of 1.5 1. Annual CVE rates ranged from 2.5–5% 26. Possible explanations for the lower‐than‐expected observed event rate in TRACE RA include: 1) event rates in randomized trials are always lower than in observational studies and the “healthy volunteer” effect may have been more pronounced than usual; 2) TRACE RA, by design, excluded patients with the highest baseline CVE risk since these patients were already being treated or had a recommendation for a statin; 3) TRACE RA participants were younger than in other statin trials and were predominantly female (as expected from RA disease demographics); and 4) <20% of participants had high disease activity at baseline.

There is increasing evidence that good disease control reduces the progression of subclinical atherosclerosis in RA patients 41, 42 and is associated with better cardiovascular outcomes. Therefore, an additional explanation for the low event rate observed in TRACE RA might have been a significant increase in the use of DMARDs, particularly biologic DMARDs, during the course of the trial. However, this was not the case. The use of prednisolone (in terms of frequency and average daily dosage), conventional synthetic DMARDs, and biologic DMARDs at baseline was balanced between the atorvastatin and placebo groups and remained so during the trial. There was not any significant increase in the use of biologic DMARDs during the trial in either group (Supplementary Section 1, available on the *Arthritis & Rheumatology* web site at http://onlinelibrary.wiley.com/doi/10.1002/art.40892/abstract). Some recent studies suggest a modest decline in CVE rates, mirroring those observed in the general population 43, 44, while others demonstrate a very substantial decline in CVE rates in RA 45. Although it is possible that CVEs may have been missed in both the atorvastatin and placebo arms, we believe this is unlikely due to the robust CVE capture system including regular patient review (80% of the patients attended the end‐of‐trial visit in both arms) and linkage with several national electronic data sources. Information from data linkage was available for all patients.

Overall, the findings of TRACE RA have important implications for clinical practice and research. The large randomized statin trials have shown that statin therapy reduces CVE risk by approximately one‐third, regardless of the level of background risk. Nevertheless, most guidelines recommend therapy only for those whose estimated individual 10‐year or lifetime risk falls above a certain threshold, for reasons of cost and risk/benefit ratio 46. TRACE RA suggests that contemporary RA patients are likely to derive the same level of benefit from statins as other populations. However, the low event rate shows that there is a sizeable population of RA patients (even among those older than 50 years or with >10 years of disease duration) who have a relatively low CVD risk. This finding does not support prescribing statins to all RA patients, one of the main questions addressed by this trial. Instead, the decision to prescribe should be based on assessment of the individual RA patient's risk using, at present, the relevant national or international recommendations and risk assessment tools 9, while disease‐specific algorithms are developed and validated 47. In terms of future research, TRACE RA provides information about effect and sample sizes that may be helpful in the design of future trials investigating CVD prevention strategies in RA, whether these are based on cardiovascular interventions, intensive inflammatory disease control, or both.

In conclusion, TRACE RA suggests that atorvastatin 40 mg daily is safe for the primary prevention of CVEs in patients with RA and appears to confer a similar degree of risk reduction in these patients as in other populations. CVE rates are decreasing in this population. This finding requires further investigation and does not support a primary prevention strategy involving statin use in all RA patients.

## Author contributions

All authors were involved in drafting the article or revising it critically for important intellectual content, and all authors approved the final version to be published. Dr. Kitas had full access to all of the data in the study and takes responsibility for the integrity of the data and the accuracy of the data analysis.

### Study conception and design

Kitas, Nightingale, Armitage, Sattar, Belch, Symmons.

### Acquisition of data

Kitas, Sattar, Belch, Symmons.

### Analysis and interpretation of data

Kitas, Nightingale, Armitage, Sattar, Belch, Symmons.

## Appendix A: The Trace RA Consortium

For the TRACE RA Consortium, members of the Trial Management Committee were as follows: chief investigator, Dr. George D. Kitas (University of Manchester and Dudley Group NHS Foundation Trust); lead investigators, Dr. Jill J. F. Belch (University of Dundee) and Dr. Deborah P. M. Symmons (University of Manchester); trial managers, Dr. Hawys Williams (University of Manchester), Mrs. Shobna Vasishta (University of Dundee), and Mrs. Rebecca Storey (Dudley Group NHS Foundation Trust); trial statistician, Dr. Peter Nightingale (University Hospitals Birmingham); senior investigators, Dr. Ian Bruce (University of Manchester), Dr. Paul Durrington (University of Manchester), Dr. Iain McInnes (University of Glasgow), Dr. Naveed Sattar (University of Glasgow), Dr. Deva Situnayake (City Hospital, Birmingham), and Dr. Allan Struthers (University of Dundee).

Members of the Trial Steering Committee were as follows: chairman, Dr. Gordon Lowe (University of Glasgow); independent members, Dr. Jane Armitage (University of Oxford), Dr. Keith Fox (University of Edinburgh Centre for Cardiovascular Science), and Dr. Dorian Haskard (Eric Bywaters Centre for Vascular Inflammation, Imperial College London); Arthritis Research UK member, Ms Caroline Dore; lay member, Ms Ailsa Bosworth (National Rheumatoid Arthritis Society), investigators, Dr. George D. Kitas (University of Manchester and Dudley Group NHS Foundation Trust), Dr. Jill J. F. Belch (University of Dundee), Dr. Deborah P. M. Symmons (University of Manchester), and Dr. Hawys Williams (University of Manchester).

Members of the Data Monitoring Committee were as follows: chairman, Dr. Michael Frenneaux (University of Aberdeen); Dr. Christopher Edwards (University of Southampton), Dr. Jonathan Emberson (University of Oxford Clinical Service Unit), and Dr. Deborah Bax (University of Sheffield).

Members of the Endpoints Committee were as follows: chairman, Dr. Stuart Cobbe (University of Glasgow); Dr. David Stott (University of Glasgow), Dr. Roger Sturrock (University of Glasgow), and Dr. Peter Macfarlane (University of Glasgow).

TRACE RA recruiting centres and principal investigators were as follows: Dudley Group of Hospitals NHS Trust (Dr. Rainer Klocke), NHS Tayside–Tayside University Hospitals NHS Trust (Dr. Tom Pullar and Dr. Su Tan), East Cheshire NHS Trust (Dr. Susan Knight), Worcestershire Acute Hospitals NHS Trust (Dr. Iain Rowe), NHS Grampian (Dr. Pradeep Kumar), Aintree Hospitals NHS Trust (Dr. Nicky Goodson), Mid Staffordshire General Hospitals NHS Trust (Dr. Diarmuid Mulherin), NHS Forth Valley (Dr. Micheal Brzeski), Western Health and Social Care Trust (Dr. Philip Gardiner), Sandwell and West Birmingham NHS Trust (Dr. Deva Situnayake), Newcastle Upon Tyne Hospitals NHS Foundation Trust (Dr. David Walker), Aneurin Bevan Health Board (Dr. Rob Callaghan), University Hospitals of Coventry and Warwickshire NHS Trust (Dr. Margaret Allen), NHS Greater Glasgow & Clyde–North Glasgow University Hospital Division (Dr. David McCarey), Wirral Hospital NHS Trust (Dr. Emmanuel George), Derby Hospitals NHS Foundation Trust (Dr. Chris Deighton), Guy's & St. Thomas’ NHS Foundation Trust (Dr. Bruce Kirkham), East Lancashire Hospitals NHS Trust (Dr. Lee‐Suan Teh), Nuffield Orthopaedic Centre NHS Trust (Dr. Raashid Luqmani), Barking, Havering and Redbridge Hospitals NHS Trust (Dr. Kuntal Chakravarty and Dr. Euthalia Roussou), Countess of Chester Hospital NHS Foundation Trust (Dr. Jenny Nixon), Poole Hospital NHS Trust (Dr. Selwyn Richards), King's College Hospital NHS Trust (Dr. David Scott), Royal Cornwall Hospitals NHS Trust (Dr. Tony Woolf), Hampshire Private Hospitals (Dr. Peter Prouse), University Hospital of North Staffordshire NHS Trust (Dr. Jonathan Packham), Pontypridd & Rhondda NHS Trust (Dr. Martin Davies), East Kent Hospitals NHS Foundation Trust (Dr. Denise DeLord), Salford Royal NHS Foundation Trust (Dr. Terence O'Neill), Nottingham University Hospitals NHS Trust (Dr. Ira Pande), NHS Highland (Dr. John Harvie), Ipswich Hospital NHS Trust (Dr. Richard Watts), University Hospital Birmingham NHS Foundation Trust (Dr. Elizabeth Rankin), Brighton and Sussex University Hospitals NHS Trust (Dr. George Papasavvas), Leeds Teaching Hospitals NHS Trust (Dr. Paul Emery and Dr. Ann Morgan), Heart of England NHS Foundation Trust (Dr. Arvind Sinha), Southend Hospital NHS Trust (Dr. Bhaskar Dasgupta), Central Manchester & Manchester Children's University Hospitals NHS Trust (Dr. Ian Bruce), North Bristol NHS Trust (Dr. Paul Creamer), NHS Lanarkshire–Acute Services Division (Dr. Asad Zoma), Sherwood Forest Hospitals NHS Trust (Dr. David Walsh), South Tees Hospitals NHS Foundation Trust (Dr. Jaap Van‐Laar), Shrewsbury and Telford Hospital NHS Trust (Dr. Nigel Capps), Belfast Health and Social Care Trust (Dr. Andrew Cairns), South Warwickshire General Hospitals NHS Trust (Dr. Christopher Marguerie), County Durham and Darlington Acute Hospitals NHS Trust (Dr. Namita Kumar), St. Helens & Knowsley Teaching Hospital NHS Trust (Dr. Rikki Abernethy), Hinchingbrooke Healthcare NHS Trust (Dr. Mark Lillicrap), NHS Lothian–Lothian University Hospitals Division (Dr. Stuart Ralston), Surrey & Sussex Healthcare (Dr. Raad Makadsi), The Royal Bournemouth and Christchurch Hospitals NHS Foundation Trust (Dr. Neil Hopkinson), Sheffield Teaching Hospitals NHS Foundation Trust (Dr. Mohammed Akil), North West Wales NHS Trust (Dr. Yasmeen Ahmad), Heatherwood and Wexham Park Hospitals NHS Trust (Dr. Matthew Adler), University Hospitals of Morecambe Bay NHS Trust (Dr. Marwan Bukhari), University Hospital of South Manchester NHS Foundation Trust (Dr. Paul Sanders), Southport and Ormskirk Hospital NHS Trust (Dr. Khalid Binymin), North Cumbria University Hospitals NHS Trust (Dr. Alaa Hassan), Ashford and St. Peters Hospital NHS Trust (Dr. Rod Hughes and Dr. Mike Irani), West Suffolk Hospital (Dr. David O'Reilly), Bradford Teaching Hospitals NHS Foundation Trust (Dr. Paul Sainsbury), NHS Borders (Dr. Ruth Richmond), Buckinghamshire Hospitals NHS Trust (Dr. Magliano Malgorzata), Burton Hospitals NHS Trust (Dr. Mohammed Nisar), NHS Greater Glasgow and Clyde (Dr. Ann McEntergart), Tameside Hospital NHS Foundation Trust (Dr. Dipak Roy), Stockport NHS Foundation Trust (Dr. Jeffrey Marks), Maidstone & Tunbridge Wells NHS Trust (Dr. Michael Batley and Dr. Taher Mahmud), Trafford Healthcare NHS Trust (Dr. Frank McKenna), NHS Fife (Dr. Helen Harris), South Eastern Health and Social Care Trust (Dr. Anita Smyth), Royal Liverpool and Broadgreen University Hospitals NHS Trust (Dr. Eddie Tunn), West Hertfordshire Hospitals NHS Trust (Dr. Adam Young and Dr. Krishnan Baburaj), James Paget University Hospitals NHS Foundation Trust (Dr. Joegi Thomas), Cambridge University Hospitals NHS Foundation Trust (Dr. Frances Hall), Norfolk and Norwich University Hospitals NHS Foundation Trust (Dr. Tarnya Marshall), Blackpool, Fylde and Wyre Hospitals NHS Foundation Trust (Dr. Chandini Rao), The Royal Wolverhampton Hospitals NHS Trust (Dr. Josh Dixey), Basildon and Thurrock University Hospitals NHS Foundation Trust (Dr. Nagui Gendi), Northumbria Healthcare NHS Foundation Trust (Dr. Fraser Birrell and Dr. David Walker), Wrightington, Wigan & Leigh NHS Trust (Dr. Gladstone Chelliah), Luton and Dunstable Hospitals NHS Foundation Trust (Dr. Daniel Fishman), Yeovil District Hospital NHS Foundation Trust (Dr. Sally Knights), City Hospitals Sunderland NHS Foundation Trust (Dr. David Coady), Surrey and Sussex Healthcare NHS Trust (Dr. Raad Makadsi), Milton Keynes Hospital NHS Foundation Trust (Dr. Bill Smith), The Pennine Acute Hospitals NHS Trust (Dr. Beverley Harrison and Dr. Sophia Naz), Abertawe Bro Morgannwg University NHS Trust (Dr. Stefan Siebert), Royal Berkshire NHS Foundation Trust (Dr Anthony Chan), Mid Cheshire Hospitals NHS Foundation Trust (Dr Kiran Putchakayala), Weston Area Health NHS Trust (Dr. Atheer Al‐Ansari), Harrogate and District NHS Foundation Trust (Dr. Andrew Gough), County Durham and Darlington NHS Foundation Trust (Dr. Namita Kumar), Barts & London School of Medicine & Dentistry (Dr. Dev Pyne), Hull and East Yorkshire Hospitals NHS Trust (Dr. Yusaf Patel), and York Hospital NHS Foundation Trust (Dr. Amanda Isdale).
