# Supplementary material for: A Multicenter, Randomized, Placebo‐Controlled Trial of Atorvastatin for the Primary Prevention of Cardiovascular Events in Patients With Rheumatoid Arthritis
Source: Arthritis Rheumatol. 2019 Jul 22;71(9):1437–49. doi: 10.1002/art.40892 (PMC6771601; doi:10.1002/art.40892)
Supplement: Supplementary file 1 [file ART-71-1437-s001.docx]

**Supplementary Files**

**CONTENTS**

Supplementary_Methods_1 FINAL PROTOCOL 2

Supplementary_Methods_2 ORIGINAL PROTOCOL 58

Supplementary_Methods_3 TRIAL HISTORY 104

Supplementary_Methods_4 TSC APPROVED STATISTICAL ANALYSIS PLAN 105

Supplementary_Figure_1 POWER CALCULATIONS 115

Supplementary_Figure_2 TRACE RA RESULTS IN THE CONTEXT OF THE 116

CHOLESTEROL TREATMENT TRIALISTS’

META-ANALYSIS OF STATIN TRIALS

Supplementary_Table_1 DETAILED TRIAL PROFILE DURING FOLLOW UP 117

Supplementary_Table_2 ADDITIONAL BASELINE CHARACTERISTICS 118

Supplementary_Table_3 COMPLIANCE AND NON-STATIN USE DURING 119

THE FOLLOW-UP PERIOD

Supplementary_Table_4 SECONDARY AND TERTIARY ENDPOINTS 120

Supplementary_Table_5 HOSPITALIZATIONS 121

Supplementary_Section_1 ANTIRHEUMATIC DRUG THERAPY AT

BASELINE, DURING THE TRIAL AND

END OF TRIAL 122

**Supplementary_Methods_1 – FINAL PROTOCOL**


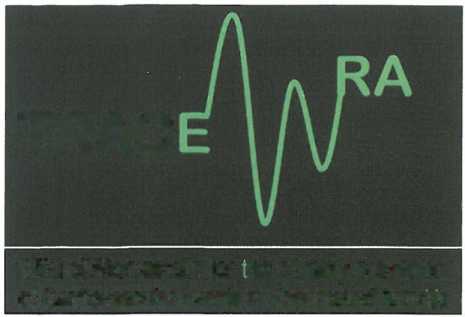


TRACE RA is supported by Arthritis Research UK and the British Heart Foundation The trial is co-sponsored by The University of Manchester and Dudley Group of Hospitals NHS Foundation Trust

PROTOCOL APPROVAL - VERSION 5.0

TRACE RA trial: TRial of Atorvastatin for the primary prevention of Cardiovascular Events in Rheumatoid Arthritis

By signing this document I am confirming that I have read, understood and approve this protocol for the above study.

Lead Investigator Professor Deborah Symmons

*&%}cT2j*

Signature Date

[Trial Statistician _0_ . , ,](#bookmark4)

[Dr Peter Nightingale 1 . C. ^!^ S / o2_ / (\ Signature Date](#bookmark5)

TO BE SIGNED BY THE LOCAL PRINCIPAL INVESTIGATOR:

‘By signing this document I am confirming that I have read this protocol and agree to abide by all provisions set forth therein. I agree to comply with the Medicines for Human Use (Clinical Trials) Regulations 2004.’

Principal Investigator Signature Date

(PRINT NAME)

MREC ref no.: 06/Q.1704/171 EudraCT no.: 2006-006032-22 ISRCTN: 41829447

TRIAL CONTACTS

Chief Investigator:

Professor George Kitas Dudley Group of Hospitals NHS Trust, Russells Hall Hospital, Dudley, DY1 2HQ Tel: 01384 244842 Fax: 01384 244808 Email: [kitas@dgoh.nhs.uk](mailto:kitas@dgoh.nhs.uk)

Lead Investigators:

| Professor Deborah Symmons |  | Professor Jill Belch |
| --- | --- | --- |
| AR UK Epidemiology Unit University of Manchester Stopford Building, Oxford Road Manchester, M13 9PL |  | Institute of Cardiovascular Research Ninewells Hospital & Medical School Dundee, DD1 9SY |
| Tel: 0161 275 5044 Fax: 0161 275 5043 Email: [deborah.symmons@manchester.ac.uk](mailto:deborah.symmons@manchester.ac.uk) |  | Tel: 01382 632457 Fax: 01382 632333 Email: [j.j.f.belch@dundee.ac.uk](mailto:j.j.f.belch@dundee.ac.uk) |
| Clinical Trial Manager: | | Clinical Trials Assistant: |
| Dr Hawys Williams |  |  |
| AR UK Epidemiology Unit University of Manchester Stopford Building, Oxford Road Manchester, M13 9PL |  | AR UK Epidemiology Unit University of Manchester Stopford Building, Oxford Road Manchester, M13 9PL |
| Tel: 0161 275 5639 Fax: 0161 275 5043 Email: [hawys.williams@manchester.ac.uk](mailto:hawys.williams@manchester.ac.uk) |  | Tel: 0161 275 1678 Fax: 0161 275 5043 Email: |

Regional Trial Co-ordinator, Dudley: Regional Trial Co-ordinator, Dundee:

| Ms Rebecca Storey |  | Mrs Shobna Vasishta |
| --- | --- | --- |
| D udley Group of Hospitals NHS Foundation Trust, Russells Hall Hospital,  Dudley, DY1 2HQ |  | Institute of Cardiovascular Research Ninewells Hospital& Medical School Dundee, DD1 9SY |
| Tel: 01384 456111 x 3733 Fax: 01384 244698 Email: [rebecca.storey@dgoh.nhs.uk](mailto:rebecca.storey@dgoh.nhs.uk) mobile: 07823 552 334 |  | Tel 01382 633954 Fax: 01382 632333 Email: [S.Vasishta@dundee.ac.uk](mailto:S.Vasishta@dundee.ac.uk) Mobile: 07921 740324 |
| Regional Trial Co-ordinator, Manchester: | | Statistician: |
| Dr Emma Knox |  | Dr Peter Nightingale |
| AR UK Epidemiology Unit University of Manchester Stopford Building, Oxford Road Manchester, M13 9PL |  | University of Birmingham The Wellcome Trust Clinical Research Facility First Floor, Blue Zone The Queen Elizabeth Hospital Edgbaston, Birmingham, B15 2TH |
| Tel: 0161 3060543 Fax: 0161 275 5043 Email: [Emma.Knox@Manchester.ac.uk](mailto:Emma.Knox@Manchester.ac.uk) |  | Tel: 0121 4721311(Ext.2586) Email: [p.g.nightingale@bham.ac.uk](mailto:p.g.nightingale@bham.ac.uk) |

SENIOR INVESTIGATORS

Prof. Ian Bruce, Professor of Rheumatology, Central Manchester NHS Foundation Trust

Prof. Paul Durrington, Professor of Medicine, Central Manchester & Manchester Children’s University

Hospital NHS Trust

Prof. Iain McInnes, Professor of Experimental Medicine, Glasgow Royal Infirmary

Dr Peter Nightingale, Trial Statistician, Wellcome Trust Clinical Research Facility, Birmingham

Prof. Naveed Sattar, Professor of Metabolic Medicine, Glasgow Royal Infirmary

Dr Deva Situnayake, Consultant Rheumatologist, City Hospital, Birmingham

Prof. Allan Struthers, Professor of Clinical Pharmacology, University of Dundee

TRIAL STEERING COMMITTEE

Chairman: Professor Gordon Lowe, Emeritus Professor, Glasgow University Independent

Members:

Professor Jane Armitage, Professor of Clinical Trials and Epidemiology & Honorary Consultant, CTSU, University of Oxford

Professor Keith Fox, Professor of Cardiology, University of Edinburgh Centre for Cardiovascular Science Professor Dorian Haskard, Director, Eric Bywaters Centre for Vascular Inflammation, Imperial College London

Members:

AR UK member:

Ms Caroline Dore, Senior Clinical Trial Statistician, AR UK Lay member:

Ms Ailsa Bosworth, Chief Executive, National Rheumatoid Arthritis Society Investigators:

Professor George Kitas, Consultant Rheumatologist, Dudley Group of Hospitals NHS Foundation Trust Professor Jill Belch, Professor of Cardiovascular and Inflammation Medicine & Honorary Consultant Physician, Institute of Cardiovascular Research, Ninewells Hospital

Professor Deborah Symmons, Professor of Rheumatology and Musculoskeletal Epidemiology, University of Manchester

Secretary: Dr Hawys Williams, Clinical Trial Manager, University of Manchester

DATA MONITORING COMMITTEE

Chairman: Professor Michael Frenneaux, Regius Professor of Medicine, University of Aberdeen

Members:

Dr Christopher Edwards, Consultant Rheumatologist, University of Southampton Dr Jonathan Emberson, Senior Statistician, University of Oxford Clinical Service Unit Professor Deborah Bax, Consultant Physician in Rheumatology, Clinical Director of Specialised Medicine, Royal Hallamshire Hospital, Honorary Professor of Rheumatology (University of Sheffield)

ENDPOINTS COMMITTEE

Chairman: Professor Stuart Cobbe, Walton Professor of Cardiology, University of Glasgow

Members:

Professor David Stott, Professor of Geriatric Medicine and Honorary Consultant, Glasgow Royal Infirmary Professor Roger Sturrock, Professor of Rheumatology, University of Glasgow Professor Peter Macfarlane, Professor of Electrocardiology, University of Glasgow

| **TABLE OF CONTENTS** | Page |
| --- | --- |
| Protocol Approval | 1 |
| Trial Contacts | 2 |
| Senior Investigators and Committee Membership | 3 |
| Trial Schema | 6 |
|  |  |
| 1. Introduction | 7 |
|  |  |
| 2. Background & Rationale |  |
| 2.1 Trial Background | 8 |
| 2.2 Rationale | 10 |
|  |  |
| 3. Trial Design |  |
| 3.1 Primary Endpoints | 11 |
| 3.2 Secondary Endpoints | 11 |
| 3.3 Trial Intervention | 11 |
|  |  |
| 4. Study Organisation | 12 |
|  |  |
| 5. Eligibility | 13 |
|  |  |
| 6. Trial Procedures |  |
| 6.1 Patient selection & Informed Consent | 15 |
| 6.2 Trial Investigations | 15 |
| 6.3 Procedure for unblinding | 19 |
| 6.4 End of Trial | 19 |
|  |  |
| 7. Discontinuation of Trial Medication | 20 |
|  |  |
| 8. Drug Supplies & Labelling |  |
| 8.1 Packaging & labelling of study medication | 20 |
| 8.2 Supply of study medication to centres | 20 |
|  |  |
| 9. Pharmacovigilance | 21 |
| 9.1 Cardiovascular outcomes and reporting Cardiovascular outcomes | 21 |
| 9.2 Adverse events | 21 |
| 9.3 Serious Adverse Reactions | 22 |
| 9.4 Suspected Unexpected Serious Adverse Reactions (SUSAR) | 22 |
| 9.5 Serious Adverse Events/Reactions (SAEs/SARs) | 23 |
| 9.6 Recording & Reporting of all SAEs/SARs | 23 |
| 9.7 Follow-Up of SAEs/SUSARs |  |
|  |  |
| 10. Statistical Considerations |  |
| 10.1 Sample size | 24 |
| 10.2 Planned recruitment rate | 24 |
| 10.3 Compliance to trial drug | 24 |
| 10.4 Planned analyses | 24 |
| 10.5 Procedure for accounting for missing data | 25 |
| 10.6 Planned sub-group analysis | 25 |
| 10.7 Interim analysis & its frequency | 25 |
| 10.8 Economic analyses | 25 |
|  | 25 |
|  |  |
| 11. Assessment of Efficacy & Safety |  |
| 11.1 Assessment of Efficacy | 26 |
| 11.2 Assessment of Safety | 26 |

| 12. Research Governance   1. Trial administration and logistics 2. Compliance to Protocol 3. Good Clinical Practice 4. Data acquisition & Monitoring 5. Data handling and record keeping 6. Archiving 7. Financial matters 8. Ethical considerations 9. Publication Policy | 27   1. 29 29 29   29  30   1. 31 |
| --- | --- |
|  |  |
| 13. Dissemination of Results |  |
| 13.1 Informing trial participants | 32 |
| 13.2 Expected value of results | 32 |
|  |  |
| 14. Confidentiality & Liability |  |
| 14.1 Confidentiality | 33 |
| 14.2 Liability/Indemnity/Insurance | 33 |
|  |  |
| References | 34 |
|  |  |
| Appendix 1: Sub-study 1: TRACE RA DAS sub-study | 37 |
| Appendix 2: Sub-study 2: TRACE RA BioBank sub-study | 39 |
| Appendix 3: Patient information Leaflets, consent forms & GP letters | 44 |
| Appendix 4: Patient lifestyle questionnaire, HAQ & EQ5D | 45 |
| Appendix 5: Definition of Endpoints | 46 |
| Appendix 6: Safety Outcomes | 52 |
| Appendix 7: Information about Atorvastatin | 53 |
| Appendix 8: Power Calculations & other statistical considerations | 55 |
| Appendix 9: Composition & Role of the TSC & DMC | 56 |
| Appendix 10: Trial Evaluations Schema | 58 |
| Appendix 11: Amendments to the study protocol | 59 |

TRACE RA TRIAL SCHEMA

|  | REVIEW OF MEDICAL RECORDS TO ASSESS ELIGIBILITY | | |
| --- | --- | --- | --- |
| No indication for a statin to be prescribed | |  |  |
|  | INFORMED CONSENT | | |
|  |  | | |

Existing indication for a statin to be prescribed

BASELINE

VISIT

BASELINE BLOOD TESTS BP, SMOKING STATUS,

HAQ, EQ5D HEIGHT, WEIGHT LIFESTYLE FACTORS CONCOMITANT MEDICATION 28 JOINT COUNT OTHER RELEVANT INFORMATION

INFORMED CONSENT, TRACE RA BIOBANK: ADDITIONAL BLOODS TAKEN


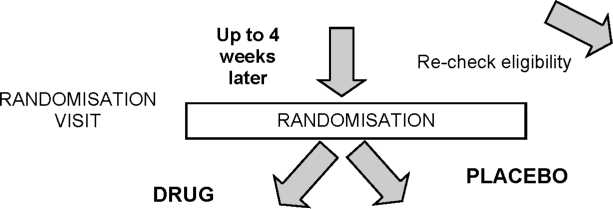
DAS 28 SCORE >3.2, INFORMED CONSENT, TRACE RA DAS SUBSTUDY

Drug Compliance/Safety Visit (3 MONTHS AFTER BASELINE VISIT)


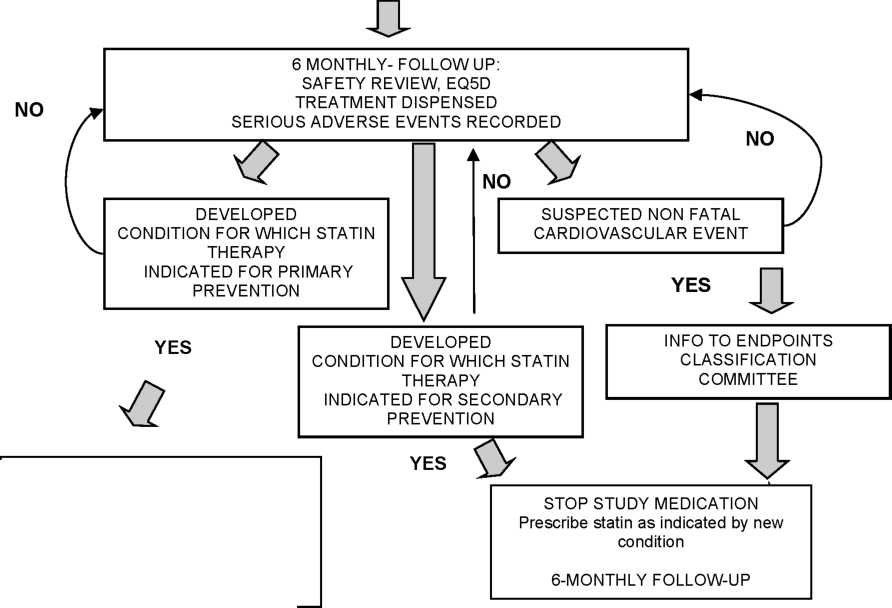


STUDY TREATMENT AND NON-STUDY STATIN (equivalent or less potent than atorvastatin 40mg) PRESCRIBED.

6-MONTHLY FOLLOW-UP CONTINUED

1. INTRODUCTION

Rheumatoid arthritis (RA) is associated with increased mortality from cardiovascular disease (CVD). This is thought to be due to accelerated atherosclerotic coronary heart disease (CHD), possibly occurring due to a combination of systemic inflammation augmenting an adverse cardiovascular risk profile.

Statins have a proven beneficial effect in reducing CVD events and mortality in at-risk populations, mostly due to their cholesterol-lowering properties, but also possibly through anti-inflammatory and immunomodulatory effects. Whether such benefit occurs in a high-grade inflammatory condition such as RA remains unknown, because such patients have been systematically excluded from previous statin trials.

This prospective, 5-year, multi-centre, randomised, double blind, placebo-controlled trial assesses the hypothesis that atorvastatin is more effective than placebo in the primary prevention of cardiovascular events in patients with RA*. All patients receive lifestyle modification advice. Patients already receiving or requiring statin therapy for secondary prevention or whose managing physician thinks they should be on a statin for an existing indication will be excluded.

A nested sub-study (TRACE RA-DAS) investigates the hypothesis that atorvastatin is more effective than placebo as adjunctive therapy in reducing RA disease activity. Patients enrolled in TRACE RA-DAS have moderately or severely active disease at the time that they are recruited to the main study.

TRACE RA and TRACE RA DAS provide a unique opportunity to create a bio bank of appropriately timed DNA, RNA, plasma and serum specimens (TRACE RA-BioBank) that would allow assessment of important supplementary hypotheses in smaller sub-studies. These will, for example, investigate the hypothesis that environmental risk factors for RA and cardiovascular disease overlap and it is likely that there are shared genetic risk factors for the onset and progression of both conditions. A detailed analysis of the molecular markers associated with atorvastatin response in RA and in particular for investigation of inflammatory markers in RA at the level of the genome will also be possible. Whereas the creation of the bio-bank forms part of the current proposal, such sub-studies will be subject to detailed protocol formation and separate ethics approval in the future.

1. Background and Rationale
   1. Trial Background
      1. Cardiovascular disease mortality (CVD) and co-morbidity in RA

Rheumatoid arthritis (RA) affects about 0.8% of the adult population in the UK [1]. It is associated with significant disability and most of the efforts of the scientific community have concentrated on controlling inflammatory symptoms, minimising joint damage and improving function. It is less well appreciated that RA is associated with increased and premature cardiovascular (CV) mortality. This has not improved much over the last 3 decades, despite significant treatment advances. Almost half of all deaths in RA (and about 35-40% of the excess deaths) are due to cardiovascular disease (CVD) [2, 3].

The excess CV deaths in RA may be due to either an increased prevalence or increased case fatality of CVD in RA compared to the general population, and there is now evidence for both of these. Overall CVD (including individual risk factors such as hypertension and dyslipidaemia) is the commonest co­morbidity in patients with RA [4, 5]. Although rheumatoid heart disease is common on echocardiography or autopsy it rarely causes haemodynamic upset, so is an unlikely cause of death [6]. Instead, the evidence suggests that the main cause of increased CV death in RA is ischaemic heart disease (IHD). Mortality studies show that most CV deaths in RA are due to ischaemic pathologies such as myocardial infarction (MI) or congestive heart failure (CHF); probably due to accelerated atherosclerotic coronary heart disease (CHD) [reviewed in 2, 3]. Indeed, functional and phenotypic surrogate markers for atherosclerotic CHD, such as endothelial dysfunction [7], increased carotid intima-media thickness [8, 9] and changes in arterial elasticity [10] are commoner or more pronounced in RA patients than in controls, as are serological surrogates such as coagulation abnormalities [11-14]. The outcome of acute coronary syndromes is also worse in RA than matched controls [15, 16], probably due to increased instability of atherosclerotic plaques and enhanced stress responses [17] associated with high-grade systemic inflammation characteristic of RA.

It is now accepted that atherosclerosis, like RA, is a chronic inflammatory condition [18]. Immunohistochemical studies suggest significant similarities between the mechanisms responsible for chronic synovitis and damage in the rheumatoid joint and the generation and rupture of the atherosclerotic plaque in the vasculature, including the cellular infiltrates adhesion molecule expression, cytokine millieux, free radical and degradative enzyme release [reviewed in 19-21]. This is further supported by epidemiological work in the general population showing that several serological markers of systemic inflammation may associate with cardiovascular outcomes. The best studied is CRP, the level of which, even within the normal range, is a good predictor of future MI or ischaemic stroke in the general population, whether there is pre-existing CVD or not [22, 23]. Baseline CRP is also a predictor of CVD death in patients with early inflammatory arthritis, even within 5 years from the onset of arthritis [24]. RA disease activity as assessed by the erythrocyte sedimentation rate, raised fibrinogen levels [25], joint swelling [26] or a composite score [27] has been shown to associate with CV events, CV death and overall mortality respectively.

- - 1. The effect of current treatments on CVD risk and outcome in RA

Thrombotic variables may be positively or negatively affected by commonly used medications, particularly non-selective non-steroidal anti-inflammatory drugs (nsNSAIDs), cyclo-oxygenase 2 inhibitors (Coxibs) and antimalarials. The effects of other treatments for RA are less clearly defined: disease-modifying anti­rheumatic drugs (DMARDs) do not appear to increase overall mortality; in contrast, effective control of inflammatory activity appears to confer survival benefits [28-31]. However, it remains to be proven whether such benefits are due to improved CV outcomes, and although suppression of inflammation in RA makes good theoretical sense, we still do not know how to achieve it - and in which patients - without compromising the vasculature. For example, compared with other DMARDs, the use of methotrexate (MTX) in RA has been reported in one study to associate with reduced overall and CVD mortality in unselected patients [28], probably due to superior control of inflammation. In another study however, use of MTX in RA patients with pre-existing CVD led to a significant increase in mortality compared with other DMARDs [32]; this may be due to its anti-folate, thus potentially hyperhomocystinaemic effects [33]. Also, use of anti-TNF or potent anti-inflammatory/immunosuppressive therapy, although effective at reducing systemic inflammation, may [34] or may not [35, 36] associate with improvements in vascular function and surrogates of atherosclerosis. This emphasises the need for prospective, randomised, controlled trials to confirm any theoretical predictions.

- - 1. Classical cardiovascular risk factors in RA

Inflammation in RA may affect classical CVD risk factors, including lipid metabolism. Dyslipidaemia has been well documented in RA and appears to associate with the acute phase response [37]. Most studies suggest that during active RA, total and LDL cholesterol may be reduced, but HDL is consistently found to be even further reduced leading to an unfavourable lipid profile. Control of disease activity with several drugs or use of ciclosporin may lead to elevation of all lipid levels with the lipid profile remaining unfavourable.

Generally, RA patients have a higher prevalence of vascular risk factors [38] and signs of asymptomatic arterial disease [39] than matched controls, which may be present even before the onset of inflammatory polyarthritis [40]. About a third of unselected hospital RA patients have documented CVD and/or CVD risk factors and are on treatment for these. Of the remaining two thirds, 90% have at least one modifiable risk factor. 70% and 60% have above the recommended levels of total cholesterol and systolic blood pressure respectively, and 40% have a >15% 10-year risk of a CHD event [13, 41]. Smoking, obesity and sedentary lifestyle may be important but are also more difficult to modify in RA than in the general population, due to the physical and psychosocial consequences of the disease [42]. As occurs in other high-risk groups (e.g. diabetes), pharmacological interventions targeted at hypertension and/or dyslipidaemia may be the most practical approach to this problem. Hypertension in RA may associate with the use of NSAIDs and Cox-2 inhibotors. Theoretically, ACE inhibitors (ACE-I) may have particularly beneficial effects in RA, since they have been shown to confer significant benefits in other high-risk populations [43], may have anti-oxidant properties and improve endothelial dysfunction [44]. However, other co-morbidities, polypharmacy, frequent treatment changes and the age groups characteristic of RA make both the routine use of such drugs problematic: for example, the combination of NSAIDs and ACE-I is commonly nephrotoxic, particularly in the elderly [45].

- - 1. Statins

Statins have a combination of properties that makes them particularly attractive for CV outcome studies in RA: they can be delivered in single daily dosing (an important factor in patients receiving multiple medications); they do not require much monitoring over and above that usually necessary for RA patients on DMARDs, and they do not interact adversely with most of the treatments commonly used in RA.

The efficacy of statins in the primary and secondary prevention of CHD events in the ‘at risk population’ has been demonstrated in several trials, with a reduction of major coronary events of 35% or above [45­51]. This is related almost exclusively to their lipid-lowering effects. There is however evidence from human in vivo studies, animal models and in vitro work, which suggest that statins have several other, so called “pleiotropic” effects. These include: reduced oxidative stress, anti-inflammatory and immunomodulatory actions, improved endothelial function, beneficial effects on vascular smooth muscle cells, antithrombotic effects and even antihypertensive properties. Several of these pleiotropic effects of statins are relevant to the atherosclerotic and chronic inflammatory pathologies of RA [52-57]. This opens up the possibility that statins may be beneficial to the rheumatoid component of RA [58, 59]. Indeed, the TARA trial [60], a ‘proof of principle’ trial demonstrated that atorvastatin 40mg daily, as an adjunct to DMARD therapy, provided additional benefit for inflammatory control of RA in at least a subgroup of patients.

With the exception of ciclosporin (which is metabolised through cytochrome P450 and can thus increase the risk of myopathy when used with some statins) there are no known significant interactions between anti-rheumatic drugs and statins. Statins are useful for secondary and primary prevention, in patients with overt CHD or those with a 10-year CVD (CHD+stroke) risk of >20%. Recent work shows that this is the case also for low risk patients, e.g. patients with diabetes with a <15% CHD risk [61]. Whereas many statin-effects may be class effects, there is evidence to suggest individual differences. Atorvastatin appears suitable for this pragmatic study. Several studies have shown a good safety profile over a wide dose range with no need to adjust dose for renal impairment. Improvements in cardiovascular end-points have been shown in many different settings and clinical populations such as ACS, stable CHD, diabetes and hypertension, including low CV risk diabetics. Early benefits seen in some atorvastatin studies have not been seen with other statins, particularly simvastatin, whereas newer statins (e.g. rosuvastatin) currently lack a large body of endpoint data. Atorvastatin may also have better effects on plaque progression and inflammatory marker reduction than either pravastatin or simvastatin [62-68]. Atorvastatin is also the only statin that has been used in an RA population with some “proof of concept” that, at a dose of 40mg daily, it can reduce systemic inflammation and improve lipid profile in RA [60], and has been shown to reduce arterial stiffness in patients with RA [69].

- 1. Rationale
     1. Need for the trial

Cardiovascular morbidity and mortality in RA are common, severe and have not received adequate attention until very recently. No trials to date have addressed whether any intervention(s) can reduce the rate of CV events in patients with RA. Articles in leading medical journals, such as the Lancet [70] clearly identify the need for sufficiently powered and specifically designed trials addressing this problem.

The cause of the CV morbidity and mortality in RA is probably multifactorial, and may be best treated by a drug with pleiotropic effects in addition to standard lipid lowering. The proposed intervention is based on sound basic science and extensive clinical trial data in other populations. However, the efficacy and safety of such an intervention remains to be proven in the RA population which, due to its major morbidity, polypharmacy and concomitant muscle pathology has been systematically excluded from all major statin trials.

- - 1. Aim of the trial

TRACE RA aims to establish whether treatment with atorvastatin will protect patients with RA aged >50 years or with >10 years’ duration of RA from fatal and non-fatal atherosclerotic events.

A nested sub-study (TRACE RA DAS) has been designed to determine whether atorvastatin, used in conjunction with standard DMARD therapy, will provide added arthritis control in patients with moderately or severely active RA.

A further sub-study, (TRACE RA BioBank) has also been incorporated into the main study. The aim of this sub-study is as follows:

1. To develop a DNA, plasma and serum repository from all patients consenting to the biobank from within those enrolled in the main TRACE RA study in centres where such blood preparation is possible, and the centres agree to provide such samples.
2. To develop an RNA, serum and plasma repository for gene expression profiling from TRACE RA DAS subjects with sampling at baseline and at 6 months.
   - 1. How the results will be used

If effective, the implementation of this intervention in the routine rheumatology clinic would be easy and, apart from the drug costs, would have no resource implications.

The trial will increase awareness of CV morbidity and mortality in RA and, in the process, identify patients who need to be properly risk-assessed and treated.

If atorvastatin also shows evidence of being a disease-modifying drug this will add to the choice of such drugs, which is at present limited. Current evidence suggests that the goal in RA treatment should be to minimise the inflammatory response - ideally aiming for remission. This is likely to slow or halt radiographic progression and to improve cardiovascular co-morbidity and mortality. All additions to the family of drugs which reduce inflammation are welcome.

1. Trial Design

Trial type

Multi-centre, randomised, double blind, placebo-controlled trial Primary hypotheses

TRACE RA - All patients: Atorvastatin is more effective than placebo in the primary prevention of cardiovascular events in RA patients.

TRACE RA DAS patients only: Atorvastatin is more effective than placebo as adjunctive therapy for the reduction of disease activity in RA patients

- 1. Primary endpoints

TRACE RA (all patients): Primary endpoints

The primary comparison will involve Cox regression analyses of “major vascular events” (defined as: coronary events [i.e. non-fatal myocardial infarction, coronary death or coronary revascularisation]; presumed ischaemic stroke or transient ischaemic attack; any non-coronary revascularisation or any other cardiovascular death excluding both confirmed cerebral haemorrhage [ICD I64-99 in the 10^th^ International Classification of Diseases]) and non-coronary cardiac death [ICD I00-I15 and I26-I52] during the scheduled treatment period among all those allocated atorvastatin tablets versus all those allocated placebo tablets (i.e. “intention-to-treat” comparisons).

TRACE RA-DAS (sub-study only):

EULAR moderate or good response based on DAS 28 at 6 months

- 1. Secondary and tertiary endpoints TRACE RA secondary endpoints

Components of the primary endpoint separately:

Coronary events;

Presumed ischaemic stroke or TIA;

Any non-coronary arterial revascularisation;

Any other cardiovascular death excluding both confirmed cerebral haemorrhage [ICD I64-99 in the 10^t^ International Classification of Diseases]) and non-coronary cardiac death [ICD I00-I15 and I26-I52]

TRACE RA tertiary endpoints

Total and cause-specific mortality (coronary, other vascular and non-vascular death separately) Hospitalisations for various other causes

Statin safety-related outcomes (persistent elevation of ALT or AST; myopathy (defined as muscle symptoms plus CK>10 x upper limit normal)

Differences in lipid levels during follow-up in a random sample Functional outcome assessed by HAQ and EQ5D

Allowance for multiple hypothesis testing in these analyses will be made using the “Bonferroni” correction.

TRACE RA-DAS secondary endpoints

DMARD (Disease Modifying and Anti-Rheumatic Drugs) changes DAS 28 at month 12 and 24.

Functional outcome assessed by HAQ and EQ5D at month 6, 12 and 24.

- 1. Trial intervention

Active treatment: Atorvastatin 40mg once daily with Placebo consisting of dummy atorvastatin once daily (both provided by Pfizer UK Ltd). All patients are counselled about modifiable cardiovascular risk factors at screening visit.

1. Study Organisation

TRACE RA aims to randomise up to 5350 RA patients, from up to 120 centres in the United Kingdom (UK). The aim is to continue recruitment until the required number of participants has been recruited to accrue sufficient person years of follow-up to achieve study power (see Section 10). The study commenced in August 2007, and recruitment is currently planned to continue until March 2014, with completion of follow up currently planned for March 2016.

The Chief and Lead Investigators plus the Trial Manager form an executive trial core management committee (TCMC). There are trials units based in Dudley, Dundee and Manchester, which oversee the management of this trial according to ICH GCP. The trials units support their affiliated recruiting centres and monitor data management for the trial. Each recruiting centre agrees affiliation to one of the trials units prior to participating in the study. However, it may be necessary to make some changes during the trial to balance the workload between trial units.

Data from all recruiting centres are being collected at the AR UK Epidemiology Unit, University of Manchester, who maintain overall responsibility for all trial data, and for the Standard Operating Procedures (SOPs) that describe how the trial is to be conducted within participating trials centres and units. All data are handled, computerised and stored in accordance with the Data Protection Act 1998. Quality control of data is maintained by the University of Manchester through regular meetings to discuss data management with the other trials units. The trials units (Dudley, Dundee and Manchester) are responsible for checking case report forms (CRFs) for compliance with the protocol, inconsistent and missing data, and for resolving data queries.

Data and statistical analysis is overseen by the Trial Statistician, Dr Peter Nightingale, who is based at the Wellcome Trust Clinical Research Facility in Birmingham.

1. Eligibility Criteria

Inclusion criteria:

- Patients who satisfy 1987 ACR classification criteria for RA applied cumulatively [71]
- Age >50 years OR RA disease duration >10 years
- Written informed consent

Exclusion criteria:

- Already taking a statin. Known cardiovascular disease deemed to require statin therapy i.e. previous episodes of confirmed Acute Coronary Syndrome (ACS), unstable angina; myocardial infarction with or without ST elevation; or stable CHD/CVD deemed to require statin therapy on clinical grounds, including:
  1. - Previous amputation due to severe peripheral vascular disease or current peripheral arterial disease -
  2. - Previous central or peripheral revascularisation procedure (including angioplasty or stent, artery bypass
  3. graft surgery)
  4. - Accelerated hypertension, severe heart failure (class III or IV), significant dysrhythmia or angina requiring
  5. hospitalisation in the 6 months preceding potential study entry
  6. - Uncontrolled hypertension (treated or untreated) defined as systolic blood pressure > 200 mmHg and/or
  7. diastolic blood pressure > 110 mmHg (identified as the disappearance of all sound (Korotkoff Phase V)
  8. after sitting quietly for at least 3 min)
  9. - Previous cerebrovascular accident
  10. - Other accepted indication for statin therapy according to the investigator’s current clinical practice
- Diabetes
- Regular use of contra-indicated drugs - see below:

Contra-indicated drugs:

Statins:

Atorvastatin (Lipitor), Fluvastatin (Lescol), Lovastatin (Mevacor), Pitavastatin (Livalo), Pravastatin (Lipostat), Rosuvastatin (Crestor), Simvastatin (Zocor),

Other contra-indicated drugs:

e.g. amiodarone, azole anti-fungals (fluconazole, ketoconazole, itraconazole), ciclosporin, fibrates, HIV protease inhibitors, macrolide antibiotics (erythromycin, telithromycin, clarithromycin), niacin, veramapil

Drugs known to affect lipid levels

e.g. colestipol, ezetimibe

Other exclusions:

- Primary muscle disease or CK >3 x ULN
- Known familial hyperlipidaemia
- Acute liver disease
- Severe renal dysfunction (Stage 3 or 4) or creatinine > 200 micromol/l or receiving renal replacement
- Uncontrolled hypothyroidism
- Hypersensitivity or intolerance to statins
- Pregnant, breast feeding or of child bearing potential not using adequate contraception*
- Alcohol abuse
- Participating in another Clinical Trial of Investigational Medicinal Product (CTIMP)
- Drinking more than 240ml of grapefruit juice per day
- Any other serious illness that may compromise safety or trial compliance

*Adequate contraception for the purpose of this trial will include the following:

Barrier Method:

Male condom Female condom Contraceptive Diaphragm Cervical cap Contraceptive Sponge Diaphragm cleaning Vaginal Spermicide Intrauterine Devices

Endocrine Method:

Combined oral contraceptives Minipills

Injectable progestins Implantable progestins

Surgical Sterilization

Female sterilization Male sterilization

1. Trial Procedures
   1. Patient selection and informed consent

Once a patient has been selected based on the eligibility criteria, sufficient time is provided for the patient to decide on trial entry, but the time which elapses between randomisation and start of treatment should be minimised (ideally no longer than four weeks).

Potentially eligible RA patients are identified from their medical records based on the presence of inclusion/exclusion criteria using the Trial Screening Form. Information on the trial is given to potentially eligible patients and they are asked to give their written informed consent. Patients are checked to ensure they are not already on a statin and do not have an existing indication for a statin according to standard clinical practice.

The Registration Form is faxed to the Manchester TRACE RA Office within 24 hours of registering a patient to the trial. A schedule of the patient’s visits (‘PCA’ - Patient Clinical Assessments) is sent to the recruiting centre to confirm receipt of the registration form.

All other documents (copy of consent forms, baseline data (‘baseline/randomisation’ form’), questionnaires, eligibility checklists) should be posted to the Manchester TRACE RA office within 7 working days.

Trial ID numbers are provided on pre-printed Patient details logs - a trial number from the CRF used for each patient is the patient’s trial number for the duration of the trial.

- 1. Trial investigations:

(Please see Appendix 10 for Trial Evaluation Schema)

Local routine practice is followed with regards to screening for 10 year cardiovascular risk. Recruiting centres that routinely screen for CVD risk continue to do so and those that do not continue to not screen. Therefore there is no requirement to measure the lipid profile of patients prior to trial entry in centres that do not routinely measure patients’ CVD risk. Where a centre routinely measures CVD risk, we recommend that the JBS2 risk calculator is used

(<http://www.bhsoc.org/Cardiovascular_Risk_Charts_and_Calculators.stm>)

- - 1. Baseline/randomisation visit

Patients are given the opportunity to ask any questions they may have, and then asked to provide their written informed consent to enter the study.

- Following this the patient has the following recorded:

^ Medical history ^ Smoking status ^ Record of concomitant medication ^ DAS 28 tender and swollen joint counts ^ Height, weight and blood pressure

- Participants are asked to complete the following questionnaires:

^ Patient global assessment (VAS)

^ HAQ [72] and EQ5D [73] questionnaires ^ Lifestyle factor questionnaire

- And asked to give a blood sample:

^ ESR and/or CRP tests: if results are available from within the last 6 weeks while their treatment has remained unchanged and the patient is stable then these results may be used instead;

^ RhF (rheumatoid factor) and/or anti-CCP (anti-cyclic citrullinated peptide antibody): if results are available from previous points during the course of their RA, then these results may be used instead.

All patients are also asked to consent to the optional TRACE RA BioBank sub study (Appendix 2).

- For TRACE RA-BioBank sub study participants (Optional)

^2 x 10mls plasma blood sample ^ 2 x 10mls serum (clotted) blood sample

^2 x 10mls EDTA blood sample (for DNA) (immediately stored at -80°C)

^ 1 x 4.5mls plasma (citrate) blood sample

Patients who have a DAS28 score of >3.2 may be asked to consent, in addition, to the TRACE RA DAS sub study (Appendix 1). The DAS28 (Disease Activity Score) [74] will be calculated using the most recent ESR/CRP. Outcome measures will be recorded in the CRF and patient hospital notes (See Appendix 6). This sub study will only be run at selected centres.

- For TRACE RA DAS sub study participants (Optional)

^ Nurse global assessment (VAS)

^ Bloods taken as for the Biobank study (as outlined above) PLUS

^4 x 2.5mls blood sample (for RNA testing) on two occasions (baseline and 6 months)

All patients are counselled by the study nurse or doctor about modifiable cardiovascular risk factors, e.g. smoking or obesity (BMI>30kg/m^2^) and given a copy of a leaflet prepared for the TRACE RA trial. GPs are informed of all significant clinical issues found at the screening visit using a standard letter.

Patients are then randomised to either the atorvastatin arm (40mg of atorvastatin oral tablet taken once daily) or placebo arm (placebo atorvastatin oral tablet taken once daily) of the trial. The patient is also registered with the trials units in order to obtain confirmation of the trial number for analysis of their medical information and subsequent dispatch of the trial drug.

- - 1. Allocation of patients to trial treatment arm

Allocation of patients to trial treatment arm: The study is double blind with matching placebo. Neither patients, investigators nor trial units are aware of the treatment allocation. The medication is provided by Pfizer UK Ltd., bottled by an independent pharmaceutical company (Catalent Pharma Solutions UK Limited) to GMP standards and dispensed by the local study pharmacist. The randomisation process is incorporated into the drug labelling. Each patient is allocated a filled and labelled bottle coded with a unique drug number when entering the trial. All future supplies for this patient will be coded with the same unique drug number. Patients will continue on the same treatment (i.e. active or placebo) throughout the duration of the study. Scratch cards are supplied to the local pharmacy so that the code can be broken if necessary. Catalent Pharma Solutions provides the independent trial statistician with information about treatment allocation by unique drug number to enable the interim analyses to be conducted. A TRACE RA Standard Operating Procedure is in the study site file for further information on providing treatment to patients.

- - 1. Follow-up visits for TRACE RA participants

Patients are asked to attend visits at 3 months, and then 6-monthly from randomisation. When attendance is not possible the CRF may be completed by telephone interview, however, every effort will be made to have an actual attendance to clinic at least once a year - in many cases this may coincide with the patient's routine clinic visit appointment. If patients are to continue study treatment, preferably ALT (and if ALT not available then AST) measurements are mandatory within 6 weeks of the follow-up visit - the overwhelming majority should be available through the patient's routine DMARD monitoring.

- Month 3 from first administration of trial drug

Every effort will be made to conduct this review appointment by clinic visit, but in exceptional circumstances it could be done by telephone interview. During this 3-month review appointment: (a) Actively seek whether muscle symptoms have occurred: if NEW and SIGNIFICANT muscle pain or weakness have occurred then measure contemporary ALT^[[1]](#footnote-1)^/AST and CK; (b) Record any serious adverse event (including any possible cardiovascular event - please see Appendix 5 for definition of cardiovascular event and Section 9 for detailed definitions of SAEs) and record length of any in-patient stay, whether for elective/planned procedures or not; (c) Review ALT/AST result: if ALT/AST available from within the last 6 weeks while patient was taking study treatment, other treatment was stable, and no new and significant muscle symptoms have been reported, then this value may be accepted and recorded in CRF with the date measurement was done; if ALT/AST not available within the last 6 weeks then request test and review result before advising patient whether they should / should not continue on study medication.

(d) Record current relevant medication.

- 6- monthly from randomization

Follow-up may be conducted either by attendance at clinic or by telephone interview, but every effort will be made to have one clinic review appointment per year. During these 6-monthly reviews act as above (3-month review) but also record HAQ and EQ5D annually since randomisation.

In the presence of new and significant muscle symptoms:

1. if CK>10xULN, then stop trial medication - not to be restarted. As indicative of myopathy, report as an SAR (Serious Adverse Reaction).
2. if CK<3xULN, trial medication can be continued with clinical follow-up. Further measurements of ALT/AST and CK can be requested if thought to be necessary by managing rheumatologist.
3. if CK between 3-10xULN retest in 1-2 weeks: (c1) if CK<3xULN then act as per option (b) above; (c2) if CK>10xULN stop trial medication and reporet as an SAR(c3) if CK between 3-10xULN retest in 1 week: if elevation persists then stop trial medication. If CK >5 x ULN on > 2 consecutive occasions, discontinue meds. If persistent elevation (> 5 x ULN) consider stopping permanently.

In the absence of any new and significant muscle symptoms:

In general, if abnormalities of LFTs with atorvastatin are to occur, they are more likely to occur within the first 3­6 months of treatment. After that, any DMARD or non-steroidal therapy (or other medications) would be a more likely cause of LFT abnormalities and the managing rheumatologists should consider stopping these drugs prior to stopping the trial medication. Attribution of LFT abnormalities to the trial medication or anti-rheumatic drug is left to the managing rheumatologist, but the TRACE RA team, including the Chief and Lead Investigators or delegated person could advise, if necessary.

If LFT abnormalities are thought to be attributable to anti-rheumatic medication rather than trial drug:

then follow usual rheumatology practice guidelines (e.g. BSR monitoring guidelines).

If LFT abnormalities are thought to be attributable to trial drug rather than other reasons:

1. If ALT/AST within normal range or <2xULN, then continue trial medication and arrange next follow-up.
2. If ALT/AST between 2-5XULN then continue trial medication and retest in 1 week. (b1) if normalisation to <2xULN has occurred, then act as per option (a) above. (b2) if ALT/AST persists at between 2-5xULN or more, then temporarily stop trial medication and act as per option (c) below.
3. If ALT/AST>5xULN continue trial medication and retest one week later: (c1) if ALT/AST has recovered to <2xULN then continue trial medication and act as per options (a) above; (c2) if ALT/AST between 2-5xULN then retest in 1 week and if ALT/AST persists at between 2-5xULN or more, temporarily stop trial medication and act as per option (c3); (c3) if ALT/AST elevation persists at >5xULN then temporarily stop trial medication and retest in 3 weeks: (c3i) if normalisation has occurred, then act as per options (a) or (b), whichever applies; (c3ii) if abnormality persists at >5xULN then stop trial medication - not to be restarted.

^2 x 10mls plasma blood sample ^ 2 x 10mls serum (clotted) blood sample ^ 1 x 4.5mls plasma (citrate) blood sample

^ Month 12 from first drug administration of trial drug (TRACE RA DAS sub-study only)

Patient Global Assessment (VAS)

Nurse Global Assessment (VAS)

28 tender and swollen joint counts ESR and/or CRP

- Month 24 from first administration of trial drug (TRACE RA DAS sub-study only)

Patient Global Assessment (VAS)

Nurse Global Assessment (VAS)

28 tender and swollen joint counts ESR and/or CRP

Patients in the TRACE RA DAS sub-study have a mandatory visit at 6 months at which the components of the DAS28 score are checked. Consultants are asked not to change the DMARD therapy of patients enrolled in the disease activity sub-study for the first 6 months of the clinical trial.

Additional visits to the rheumatology clinic and changes in medication will be decided based on clinical need. Patients will be given the telephone number of their consultant or Rheumatology department/helpline in case they have any concerns whilst enrolled on the trial.

Patients will continue the intervention for the entire period of their participation in the trial, unless the trial steering committee decides to discontinue the study for any reason. Any patients who discontinue the intervention will remain under follow up.

- 1. Procedure for unblinding

Each hospital pharmacy has a nominated person who can break the randomisation code for an individual patient if required in a medical or other emergency. The code (scratch card) for each individual patient is kept securely in the pharmacy. The local and main co-ordinating centre is informed each time the code is broken and is given the reason for the unblinding.

- 1. End of trial

The trial is currently planned to continue recruiting until March 2014 with the last follow-up appointment being in March 2016, or until a sufficient number of events has accrued to provide statistical power (currently calculated to be around 370 confirmed primary events) whichever is the earlier. All patients will be followed-up (by phone or attendance) 6-monthly until the trial has been completed and will stop their medication on the day of their final assessment. All events which occur prior to this final assessment will be included in the analysis whether or not the patient has continued to take the trial medication. Because of the ‘intention-to-treat’ (ITT) analysis, it is essential to follow all patients (including those that have reached a trial endpoint) up to this ‘final assessment’ date.

1. Discontinuation of Study Medication

Patients who experience a cardiovascular event will stop their study medication and take statin prescribed by their managing doctor. They will continue to be followed-up. Patients who, during the course of the trial, develop other indications for statin therapy (e.g. diabetes) can continue on the trial medication AND be prescribed unblinded statin, preferably atorvastatin up to a maximum dose of 40mg daily, or an alternative statin up to a maximum dose equivalent to atorvastatin 40mg daily. If a higher dose than this is required then the trial medication will be stopped and an unblinded statin prescribed. A record of any concomitant statin prescription will be made on the CRF. All patients will also continue to be followed-up in the trial.

If the trial is stopped early for safety reasons, all patients will stop the study medication. If it is stopped early due to efficacy of the statin arm, all patients will be offered the opportunity to continue/commence statin therapy. If the trial goes its full length, all patients will stop the trial medication and be managed according to their physician’s preference pending full analysis and publication of the trial findings.

Patients are free to withdraw from the trial treatment at any point. This will have no implications on their future care.

Breaks in study medication: Should patients take a break in their study medication, they may re­commence at any time provided /ALT/AST is within the screening limits within the last 6 weeks and other treatment has remained stable. ALT (or AST if ALT not available) should be checked 3 months after restarting study drug.

1. Drug Supplies and Labelling

Atorvastatin and the placebo atorvastatin are supplied free of charge by Pfizer UK Ltd.

- 1. Packaging and labelling of study medication

The drug is packaged in bottles and labelled by Catalent Pharma Solutions according to GMP standards. Bottles are labelled with a minimum of the following information:

- Unique drug number (number allocated to drug bottle)
- Packaging lot number
- Expiry date
- Number of tablets in bottle
- Dosage instructions
- Storage conditions

Bottling and labelling will be done in several runs spaced throughout the trial. All bottles contain extra tablets to allow for delays in patient visits.

- 1. Supply of study medication to centres

Catalent Pharma Solutions supplies the packaged drugs to the pharmacies of individual hospitals at regular intervals throughout the trial.

Patients are asked to return any unused medication to pharmacy which will be destroyed on site.

Patients are provided with a 6 monthly supply of the study medication. At the first visit they are advised that they must attend for a safety visit at 3 months and should only continue the medication beyond this point if advised to do so. Each time that the trial drug is dispensed, the pharmacy will be asked to affix the supplementary drug label to the prescription sheet so that a ‘dispensing record’ can be maintained.

1. Pharmacovigilance
   1. Cardiovascular Outcomes and reporting of cardiovascular outcomes

Any cardiovascular event or endpoint experienced by a patient should be reported on the serious adverse events form that is provided in the site file, as these events will be classified as part of the endpoints of the trial. All information regarding a cardiovascular event should be faxed to the AR UK Epidemiology Unit at the University of Manchester on 0161 275 5043 within 24 hours of knowledge of the event. The local principal investigator or research nurse will be contacted by the trials unit if further information is required.

- 1. Serious Adverse Events (SAEs)

A Serious Adverse Event (SAE) is defined as any untoward medical occurrence that:

- results in death
- is life-threatening (i.e. with an immediate, not hypothetical, risk of death at the time of the event),
- requires hospitalisation or prolongs existing hospitalisation
- results in persistent or significant disability or incapacity,
- is a congenital anomaly or birth defect (i.e. the outcome of pregnancy involving the patient)
- any other important medical condition which, though not included in the above, may jeopardise the patient and may require medical or surgical intervention to prevent one of the outcomes listed (e.g. allergic bronchospasm requiring intensive emergency treatment, seizures or blood dyscrasias which do not result in hospitalisation, or development of drug dependency).

Medical judgement should be exercised in deciding whether an adverse event/reaction is serious in other situations. Hospitalisations include planned admissions for elective surgery.

All serious adverse events must be reported on the serious adverse event form. The SAE form should be faxed to the AR UK Epidemiology Unit at the University of Manchester on 0161 275 5043 within 7 days of knowledge of the event. The local principal investigator or research nurse will be contacted by the trials unit if further information is required.

- 1. Adverse Events

An adverse event is the development of an undesirable medical condition or the deterioration of a pre-existing medical condition following or during exposure to a pharmaceutical product, whether or not considered causally related to the product. An undesirable medical condition can be symptoms (eg, nausea, chest pain), signs (eg, tachycardia, enlarged liver) or the abnormal results of an investigation (eg, laboratory findings, electrocardiogram).

Adverse events (either serious or non-serious) which lead to discontinuation of study treatment should be routinely recorded on the CRF. Other adverse events need not be recorded in the CRF.

in addition, new significant muscle symptoms should be sought at each study visit and recorded. If present AND if ALT is greater than the upper limit of normal then a blood CK should be measured (see section 6.2.3)

- 1. Serious Adverse Reactions (SAR)

SERIOUS (as defined in Section 9.2) adverse events, judged by the reporting investigator as having a reasonable causal relationship to atorvastatin, qualify as serious adverse reactions. This judgement of causality should be made without breaking the randomisation code - on the assumption that the patient has been exposed to atorvastatin.

SARs should be reported on the serious adverse event report form and faxed to the AR UK Epidemiology Unit within 24 hours of knowledge of the event.

- 1. Suspected Unexpected Serious Adverse Reactions (SUSARs)

For any reported SAR, the assessment of ‘expectedness’ will be made by the Chief Investigator (or their delegated deputy) based on the current Summary of Product Characteristics (SmPC)/Package Insert for atorvastatin. If confirmed as a SUSAR will be subject to expedited reporting by the trial sponsor (i.e. Chief Investigator and University of Manchester) to MHRA, therefore every effort should be made to notify the regional trials office within the timeframe shown below (Section 9.7). As a general rule, the treatment code for the specific patient should be broken before reporting a SUSAR to MHRA. Events associated with placebo will usually not satisfy the criteria for a SUSAR.

- 1. Reporting of SAEs, SARs and SUSARs to Pfizer

Although this is an investigator led clinical trial in which Pfizer (the Market Authorisation Holder for atorvastatin) is not acting as sponsor, there is a regulatory requirement that Pfizer be notified of all SAEs, SARs and SUSARs in a timely fashion. This reporting will be carried out by the co-ordinating centre at the University of Manchester and will be separately financed by Pfizer.


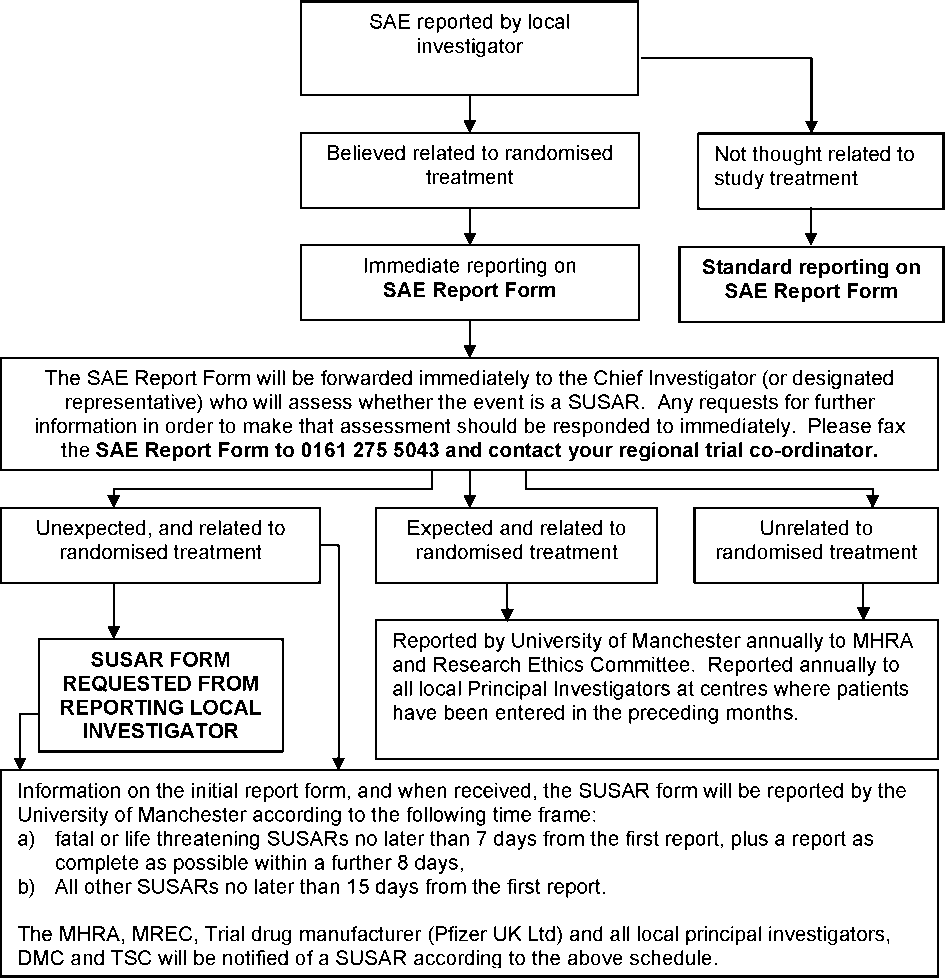


The patient should be followed-up until clinical recovery is complete and laboratory results have returned to normal, or until disease has stabilised. Information on final diagnosis and outcome of SAEs which may not be available at the time the SAE is initially reported should be forwarded on a copy of the SAE initial report form as soon as this information is available. Follow-up may continue after completion of protocol treatment if necessary.

9.7 Recording and reporting of all SAE/SARs

Flow diagram of SAE reporting and action taken following the report:

All information regarding an SAE should be faxed to the University of Manchester on 0161 275 5043, and information required by the drug manufacturer will be passed on by that trials unit. Centres are free to volunteer information to the drug manufacturer if they wish, but are under no obligation to do so.

1. Statistical Considerations
   1. Sample size

The aim of the trial is to detect (with 80% power at the 5% significance level) a 32% relative risk reduction (RRR) in the primary endpoint attributable to treatment with atorvastatin and anticipating an average of 25% non-compliance. If the true effect of taking the treatment is a 32% RRR but only 75% of patients are compliant, then the effect seen in this trial should be about 24% (ie, 75% of 32%). The required number of first events (i.e. patients with events) to be able to detect such a reduction with 80% power at the 5% significance level is 434 (Appendix 8). The estimated average blinded event rate in all patients is expected to be 1.6-1.8% per annum. A maximum of around 27000 patient-years of follow-up is required for these event rates to produce the required number of first events (Appendix 8). Based on recruitment rates achieved between August 2007 and November 2010 and using a logarithmic decay model to reflect the decline in recruitment with time, it is estimated that 5342 patients recruited by March 2014 would provide between 27000 and 28000 patient-years of follow-up. If the blinded event rate is somewhat less than expected then the trial will continue (if necessary) until the required number of events has been accrued and all the available follow-up data will be used in the analysis.

- 1. Planned recruitment rate

With the anticipated support of up to 120 centres/sites within the UK, the logarithmic decay model predicts that the required number of patients will be recruited by March 2014 (assuming follow-up until March 2016).

The trial manager and trial co-ordinators will continue to provide support to their respective recruitment centres and the relevant research nurses employed through the CLRN and other mechanisms.

The trial will continue to be publicised through the AR UK’s “Arthritis Today” magazine and through the National Rheumatoid Arthritis Society (NRAS) as well as local resources. A British Society for Rheumatology (BSR) special interest group has been set up to act as a forum for the study investigators and TRACE RA news are regularly discussed in the quarterly BSR leaflet. A TRACE RA website has also been established.

- 1. Compliance

Compliance will be estimated based on patient reports on Case Report Forms and patients will be considered compliant if they report taking ‘most’ of their study tablets since their last visit. In addition, the difference in LDL cholesterol will be measured in a random sample of 400 participants in June 2011 and at intervals during the trial to allow the average difference in LDL to be estimated.

- 1. Planned analyses

All patients randomised will be included in the analysis, irrespective of whether the study drug is continued (ITT analysis). Cox regression models will be developed for time to occurrence of a first cardiovascular event or serious adverse event using treatment allocation as the independent variable.

The models will be adjusted for factors used in the stratification (centre) and for any baseline imbalances. However, the trial is of sufficient size to expect that most potential confounders (e.g. smoking, aspirin, NSAID/Coxib, corticosteroid, anti-TNF usage etc) are likely to be balanced between the groups. Treatment differences will be expressed as hazard ratios with 95% confidence intervals. Two-sided p values of <0.05 will be considered significant.

Kaplan-Meier Product Limit (PL) estimates of the survival curves with 95% confidence intervals will be calculated.

Analysis of rheumatology outcomes

All patients randomised in the TRACE RA DAS substudy trial will be included in the analysis, irrespective of whether the study drug is continued (intention-to-treat analysis). The dependent variable will be the dichotomous variable of EULAR response. Any further baseline variables can be incorporated into the model if necessary to adjust for baseline imbalances. Treatment differences will be expressed as odds ratios with 95% confidence intervals. Two-sided p values of <0.05 will be considered significant.

- 1. Procedure for accounting for missing data

Tests will be conducted to explore whether missing data are missing at random and appropriate procedures then deployed to impute the missing data.

- 1. Planned sub-group analyses

Sub-group analyses will be carried out with respect to: sex; age (<65 vs. >65 years); RA disease duration; rheumatoid factor status and anti-TNF therapy (if numbers are sufficient). It is recognised that these sub­analyses will have less statistical power than those based on the whole study sample and this will be reported appropriately.

- 1. Interim analyses and its frequency

Interim analyses will be performed when the number of events reaches 25%, 50% and 75% of the total expected events. No formal stopping rule will be set but the DMC will advise the Chairman of the Trial Steering Committee that the trial should be stopped if, in DMC’s view, the randomised comparison in the trial has provided both (a) proof beyond reasonable doubt that for all, or for some, types of patients the trial treatment is clearly indicated or clearly contraindicated in terms of a net difference in major morbidity and mortality, and/or (b) evidence that might reasonably be expected to influence the patient management of clinicians aware of the results of any other studies. Safety data will be continuously monitored by the independent DMC.

- 1. Economic analyses

The trial will not address any economic issues directly but, since EQ5D can be used for health utility assessment [75], the results can subsequently be used for economic modelling and cost-effectiveness based on calculation of QALYs gained.

The trial will also monitor the number of hospital admissions and length of stay that occur via information collated from the CRFs.

1. Assessment of Efficacy and Safety
   1. Assessment of efficacy outcomes

The records of English and Welsh randomised patients’ will be electronically tagged for mortality with the Office for National Statistics (ONS) and Scottish patients at the Scottish Office’s Information and Statistics Division (ISD) as well as the local hospital Trusts’ Medical Information departments. Underlying cause of death will be ascertained from death certificates provided by ONS and ISD (which also supply details of times and causes of all hospital admissions in Scotland) supplemented by information from hospital records, including post-mortem examinations, if performed.

Hospital admissions will be ascertained at each centre by matching the patient details with national NHS Hospital Episode Statistics. In addition, non-fatal events will be ascertained regularly by contact with each patient. If necessary additional information will be sought from household members, GP or hospital departments.

- 1. Assessment of safety

Statin safety-related outcomes, as stated in Appendix 6, will be monitored by the independent DMC. The only 2 recognised adverse effects of atorvastatin are:

- Myopathy (muscle pain or weakness in association with a raised CK>10x ULN); and
- Liver function abnormalities

These will be monitored at each follow-up by reviewing ALT or AST results and asking about new and significant muscle symptoms.

In the presence of new and significant muscle symptoms:

1. If CK>10xULN, then stop trial medication - not to be restarted.
2. If ALT/AST<2xULN and CK are <3xULN, trial medication can be continued with clinical follow-up. Further measurements of ALT/AST and CK can be requested if thought to be necessary by managing rheumatologist.
3. If ALT/AST >2xULN and/or CK between 3-10xULN retest in 1-2 weeks: (c1) if normalisation has occurred to ALT/AST<2xULN and CK<3xULN then act as per option (b) above; (c2) if CK>10xULN stop trial medication; (c3) if ALT/AST>2xULN and CK again between 3-10xULN retest in 1 week: if elevation persists then stop trial medication.

In the absence of any new and significant muscle symptoms:

In general, if abnormalities of LFTs with atorvastatin are to occur, they are more likely to occur within the first 3-6 months of treatment. After that, any DMARD or non-steroidal therapy (or other medications) would be a more likely cause of LFT abnormalities and the managing rheumatologists should consider stopping these drugs prior to stopping the trial medication. Attribution of LFT abnormalities to the trial medication or anti-rheumatic drug is left to the managing rheumatologist, but the TRACE RA team, including the Chief and Lead Investigators or delegated person could advise, if necessary.

If LFT abnormalities are thought to be attributable to anti-rheumatic medication rather than trial drug: then follow usual rheumatology practice guidelines (e.g. BSR monitoring guidelines).

If LFT abnormalities are thought to be attributable to trial drug rather than other reasons:

1. If ALT/AST within normal range or <2xULN, then continue trial medication and arrange next follow-up.
2. If ALT/AST between 2-3xULN then continue trial medication and retest in 1 week. (b1) if normalisation to <2xULN has occurred, then act as per option (a) above. (b2) if ALT/AST persists at between 2-3xULN or more, then temporarily stop trial medication and act as per option (c) below.
3. If ALT/AST>3xULN continue trial medication and retest one week later: (c1) if ALT/AST has recovered to <2xULN then continue trial medication and act as per options (a) above; (c2) if ALT/AST between 2- 3xULN then retest in 1 week and if ALT/AST persists at between 2-3xULN or more, temporarily stop trial medication and act as per option (c3); (c3) if ALT/AST elevation persists at >3xULN then temporarily stop trial medication and retest in 3 weeks: (c3i) if normalisation has occurred, then act as per options (a) or
4. , whichever applies; (c3ii) if abnormality persists at >3xULN then stop trial medication - not to be restarted.
5. Research Governance
   1. Trial administration and logistics

Dudley Group of Hospitals NHS Foundation Trust and the University of Manchester are co-sponsors of the TRACE RA trial. Sponsorship activities and delegated responsibilities are shared between Dudley Group of Hospitals NHS Foundation Trust, the employer of the Chief Investigator (CI); and the University of Manchester, in accordance with the UK Medicines for Human Use (Clinical Trials) Regulations 2004 and in line with the Research Governance Framework for Health and Social Care, April 2005 2^nd^ Edition, and according to ICH GCP. Both parties agree to allow inspection of sponsors’ premises by the competent authorities.

Dudley Group of Hospitals NHS Foundation Trust (DGOH) & Chief Investigator responsibilities:

- Put and keep in place arrangements to adhere to ICH GCP
- Ensure that Investigational Medicinal Products (IMPs) are made available to subjects free of charge
- Take appropriate urgent safety measures
- Ensure that Pharmacovigilance is maintained throughout the duration of the trial - please note that the administration of Pharmacovigilance for the trial has been delegated to the University of Manchester by the Chief Investigator.
- Ensure that PIs conduct the study in accordance with ICH GCP, the DOH Research Governance Framework and laws and statutes that relate to the study and any local requirements as may be specified by their host institution.
- Responsibility for putting and keeping in place arrangements to conduct the study according to Good Clinical Practice, the DOH Research Governance Framework and the laws and statutes that relate to the study
- Responsibility to use all reasonable efforts to ensure that the data collected and reported are accurate, complete and identifiable at source; and that record keeping and data transfer procedures adhere to the Data Protection Act 1998.
- Responsibility for monitoring the study in accordance with the arrangements outlined in the submission to the Sponsor.
- Responsibility to supply documentation and reports as deemed necessary by the Sponsor to fulfil its obligations.
- Responsibility to co-operate with audits or inspections undertaken by the host institution, the Sponsor and regulatory authorities, including the MHRA, as required.
- Responsibility to assist investigations into any alleged research misconduct undertaken by or on behalf of the Sponsor.
- Responsibility to make the necessary provision for archiving essential documents.

AR UK Epidemiology Unit, University of Manchester responsibilities:

Trial Administration Responsibilities:

- Request Clinical Trial Authorisation (CTA) and make any amendments that are required for the authorisation
- Undertake to allow inspection of co-sponsors premises
- Gain appropriate authorisations prior to starting the trial, including authorisation from both the NHS Trust research offices and the University of Manchester, ethical approval.
- Give notice of the following events to the appropriate regulatory bodies:

o amendments to CTA, make representations and amendments o amendments to the protocol

o the termination of the trial

- Maintain a Master File containing essential trial documents and to make the file available for statutory inspections by bodies such as the MHRA

Financial Responsibilities:

- Administer funding and co-ordinate any required legal agreements and investigator statements or agreements.

Responsibilities that have been delegated by DGOH & CI:

- Keep records of all adverse events reported by investigators
- Ensure recording and prompt reporting of suspected unexpected serious adverse reactions (SUSARs) to the Chief Investigator
- Ensure investigators are informed of SUSARs
- Provide an annual list of suspected adverse reactions and a safety report to the relevant authorities and committees.

The following responsibilities are retained by the Chief Investigator, or in his absence, a named deputy(s):

- Prompt decision making as to which serious adverse events are SUSARs, and prompt reporting of that to the University of Manchester for onward reporting to the licensing authority.

The following responsibilities are delegated by the Chief Investigator to the local Principal Investigators at each trial centre:

- Obtain Management (R&D/ Research Governance) approval
- Responsibility for putting and keeping in place arrangements to conduct the study according to Good Clinical Practice, the DH Research Governance Framework and the laws and statutes that relate to the study
- Responsibility to liaise with Pharmacy to document the supply, handling and accountability of all trial drugs
- Responsibility to ensure that all members of the study team have sufficient knowledge, training and experience to undertake the roles assigned to them and to comply with requirements as specified by the host organisation
- Responsibility to maintain a Site File (containing the essential documents) and to make the site file available for inspection if requested by the CI (on behalf of the Sponsors)
- Responsibility to conduct the study in accordance with the agreed research protocol except where necessary to eliminate (an) immediate hazard(s) - These circumstances must be reported to the CI who will be responsible for reporting these events on behalf of the sponsor organisations, to the research ethics committee and the MHRA
- Responsibility to use all reasonable efforts to ensure that the data collected and reported are accurate, complete and identifiable at source; and that record keeping and data transfer procedures adhere to the Data Protection Act 1998
- Responsibility to supply documentation and reports as deemed necessary by the Sponsor
- Responsibility to cooperate with audits or inspections undertaken by the host institution, the Sponsors and regulatory authorities, including the MHRA as required.
- Responsibility to assist investigations into any alleged research misconduct undertaken by or on behalf of the Sponsors
- Responsibility to make the necessary local provision for archiving essential documents

The delegation of sponsorship responsibilities does not impact on or alter standard NHS indemnity cover. The agreement of delegated responsibilities is viewed as a partnership and as such it is necessary to share pertinent information between the University of Manchester and the Dudley Group of Hospitals NHS Trust/Chief Investigator, including proposed inspections by the MHRA and/or other regulatory bodies.

- 1. Compliance with Protocol

TRACE RA is being conducted in accordance with the professional and regulatory standards required for

non commercial research in the NHS under the UK Medicines for Human Use (Clinical Trials) Regulations

2004. Before activating the trial, participating centres are required to sign an agreement accepting delegated responsibilities for all trial activity which takes place within their centre.

- 1. Good Clinical Practice

This trial will be conducted in accordance with the protocol, the conditions and principles stipulated in the Medicines for Human Use (Clinical Trials) Regulations 2004 and all other applicable regulatory requirements.

- 1. Data acquisition and monitoring

Trials unit staff will visit the participating centres to confirm that agreements are being adhered to, specifically to carry out source data verification and confirm compliance with the protocol and the protection of patients’ rights as detailed in the Declaration of Helsinki. Copies of the Declaration may be obtained from the designated regional trials unit. By participating in the TRACE RA trial, Principal Investigators at each centre are confirming agreement with his/her local NHS Trust to ensure the following:

- Sufficient data is recorded for all participating patients to enable accurate linkage between patient hospital records and trial case report forms.
- Source data and all trial related documentation are accurate, complete, maintained and accessible for monitoring and audit visits
- All staff at their individual centres who are involved with the trial will meet the requirements of working within the statutory provisions of UK law.
- Original consent forms are dated and signed by both patient and investigator and are kept together in a central log together with a copy of the specific patient information sheet(s) given to the patient at the time of consent. The original consent form must be kept in the centre site file and copies of the consent form should be given to the patient, filed in the patient hospital notes and also forwarded to the regional trials unit.
- Copies of CRFs are retained for 15 years at the NHS Trusts and the University of Manchester to comply with international and organisational regulations
- Staff will comply with the Standard Operating Procedures for TRACE RA

The affiliated trials units will monitor receipt of CRFs, evaluate incoming CRFs for compliance with the protocol and resolve inconsistencies and missing data queries.

Participating centres will be monitored by their allocated trials unit and also possibly by the relevant regulatory authorities. Monitoring by the units will confirm compliance with the protocol and source data verification (SDV). The Trial Manager (based in Manchester) will establish quality assurance proformas to be completed by the trial co-ordinators when visiting the participating NHS Trusts. Some combined visits between the trials units will be conducted to ensure consistency of approach.

The frequency of monitoring will be determined according to a risk assessment model approved by the TSC. If any problems are detected in the course of the monitoring/auditing visits, then the Principal Investigator and the trials unit will work together to resolve queries.

- 1. Data handling and record keeping

All data will be entered on to a computerised database at the trials units. All data will be identified via a unique trial number and data tables will be linked using this number. The names and addresses of patients matched to their trial number will be stored in a separate secure database. All databases will be password protected and stored according to the requirements of the Data Protection Act 1998.

- 1. Archiving

All source and study documentation must be securely retained by the local Principal Investigator for 15 years after the trial has ended. An end of study visit may be performed by the trials units to resolve any data queries and outstanding trial documentation before the documentation can be archived by the participating centre. Source data (including data on any patients who die) must be retained for the duration of the recruitment, treatment and follow up phases of the trial for inspection by representatives of trials units.

- 1. Financial matters

TRACE RA is investigator-designed and led, and is jointly funded by Arthritis Research UK and the British Heart Foundation. Pfizer UK Limited has provided free active trial drug and placebo for the whole of TRACE RA, as well as an unrestricted educational grant for the TRACE RA-DAS and the TRACE RA BIOBANK sub-studies. If additional financial support is received from any other source, this will be made apparent to the approving MREC but will not require a protocol amendment.

Cost implications to the NHS Trusts:

Support for local set-up costs (£300 per centre), local pharmacy set-up and running costs (£200 per centre) and local research nurse time (£70 per recruited patient) will be made available to collaborating NHS Trusts. Invoicing should be raised by the participating trial centres to their respective trials units. The trials centres will be contacted at the end of trial recruitment to ensure that payment has been received by the centre for the services provided. Additional support in terms of human resources will be provided to the trial centres by the trial units, through the trial co-ordinators (for the duration of the trial) and centrally-appointed trial nurses (for the initial 2 years of the trial), subject to satisfactory honorary contracts and reciprocal agreements.

The trial and its substudies have all been adopted by the UKCRN Clinical Research Network and are therefore eligible for additional support through this funding stream via each Comprehensive Local Research Network (CLRN),

- 1. Ethical considerations

The original trial protocol was submitted for ethical review to COREC. The MREC ref no is 06/Q1704/171. The trial did NOT commence recruitment until central (MREC & MHRA) and individual site (SSI & R&D) regulatory approvals were in place at each recruiting centre. Any subsequent amendments (including this revised protocol) will be submitted to the same MREC and will not be implemented until approval has been received and notified to all participating sites.

The main ethical consideration pertaining to the TRACE RA trial is whether the trial should be terminated early if efficacy of atorvastatin is proven. The planned interim analyses should ensure that the trial continues only as long as is needed to establish, beyond reasonable doubt, that treatment with atorvastatin is either beneficial or harmful to patients with RA.

- 1. Publication Policy

The main trial results will be published in the name of the trial in a peer-reviewed journal, on behalf of all collaborators. The trials units and all participating centres and clinicians will be acknowledged in this publication.

All presentation and publications relating to the trial must be authorised by the TRACE RA Trial Steering Committee.

No investigator may, at any time, present or attempt to publish data relating to the TRACE RA trial and its sub studies without prior permission from the Trial Steering Committee.

1. Dissemination of Results
   1. Informing trial participants

Participating centres will be sent a list of their patients along with their allocated treatment arm at the end of the trial. They will then be able to inform those patients who wish to know which treatment arm they were allocated.

- 1. Expected value of the results

The proposed trial will add to the evidence base of how to reduce the cardiovascular risk of patients with RA. It may identify an intervention that will reduce the incidence of the most common cause of death in the most common form of chronic inflammatory arthritis. The trial will also be used to define RA specific CV risk profiles, which can be utilised to identify patients at high risk and inform the need for further investigation and treatment.

Indirectly, this trial may provide some insight into the link between inflammation (in this case, high-grade inflammation) and CVD and provide mechanistic clues that can be investigated specifically in subsequent studies. TRACE RA BioBank will be an excellent resource to address such questions at the basic scientific level.

The TRACE RA-DAS sub-study will add to the evidence base of compounds which have disease modifying properties in RA. If benefit is shown, it is likely that atorvastatin might then be used in combination with other DMARDs to improve overall disease control. This information would be of most benefit in patients with a known high CVD risk.

1. Confidentiality and Liability
   1. Confidentiality

The trials units (Dudley, Dundee & Manchester) will comply with all aspects of the Data Protection Act 1998. All information collected during the course of the trial will be kept strictly confidential.

Patients NHS numbers will be collected on a patient eligibility and registration form (this will be returned to Manchester Trials Unit) at the beginning of the trial. All other data collection forms, which are faxed/posted to the trials unit, will be coded with 3 patient identifiers (Patient's Initials, Centre numbers and Patient Identification Number). Information will be held securely on paper and electronically at the trials units, including appropriate storage, restricted access and disposal arrangements of patients' personal and clinical details. Participants will also not be identified in the results of the study.

Patient information recorded in their medical records will be accessed during identification of potential patients and when site monitoring visits occur to ensure that the trial is being carried out according to ICH GCP guidelines.

Stored patient information will be kept on NHS and University computers so as to be able to track the number of patients on the trial. Data from patients’ medical records will be transcribed onto case report forms.

All clinical information about patients will be stored on a central database at the University of Manchester. The main database and any sub-databases will be password protected and stored according to the requirements of the Data Protection Act 1998.

On occasion, if quality of life questionnaires are not completed in clinics due to time constraints or patients not attending clinic, the questionnaire may be posted by the research nurse for patients to complete and return to the University of Manchester Trials Unit (a SAE will be provided to facilitate this).

The local principal investigators must keep a separate log of patients’ trial numbers, names, addresses and hospital numbers. The local principal investigator must maintain in strict confidence trial documents, which are to be held in the local hospital (e.g. patients’ written informed consent forms). The local principal investigator must also ensure that patient confidentiality is maintained.

The trials units will maintain confidentiality of all subject data and will not reproduce or disclose any information by which subjects could be identified, other than reporting of serious adverse events. Representatives of the trials units will be required to have access to patients medical records for quality assurance purposes but patients should be assured that their confidentiality will be respected at all times. This will be stated in the patient information sheet.

- 1. Liability/ Indemnity/ Insurance

The individual NHS trusts have a duty of care to patients treated, whether or not the patient is taking part in a clinical trial and the NHS trusts remain liable for clinical negligence and other negligent harm to patients under this duty of care. Indemnity for participating hospitals is provided by the usual NHS indemnity agreements.

References

1. Symmons DP, Turner G, Webb R et al. The prevalence of rheumatoid arthritis in the United Kingdom: new estimates for a new century. Rheumatology 2002; 41: 793-800
2. Goodson N. Coronary artery disease and rheumatoid arthritis. Curr. Opin Rheumatol. 2002; 14: 115-120
3. Kitas GD, Erb N. Tackling ischaemic heart disease in rheumatoid arthritis. Rheumatology 2003; 42: 607-13
4. Kroot EJJA, van Gestel AM, Swinkels HL, et al. Chronic comorbidity in patients with early rheumatoid arthritis: a descriptive study. J Rheumatol 2001; 28: 1511-7
5. Kitas GD, Banks MJ, Bacon PA. Cardiac involvement in rheumatoid disease. Clin Med JRCPL 2001; 1: 18-21
6. Goodson NJ, Solomon DH. The cardiovascular manifestations of rheumatic diseases. Curr. Opin. Rheumatol. 2006; 18(2): 135-40
7. Bacon PA, Raza K, Banks MJ, Townend J, Kitas GD. The role of endothelial cell dysfunction in the cardiovascular mortality of RA. Int Rev Immunol 2002; 21(1):1-17
8. Park YB, Ahn CW, Choi HK, Lee SH, In BH, Lee HC et al. Atherosclerosis in rheumatoid arthritis: morphologic evidence obtained by carotid ultrasound. Arthritis Rheum 2002; 46(7):1714-1719.
9. Alkaabi JK, Ho M, Levison R, Pullar T, Belch JJF. Rheumatoid arthritis and macrovascular disease. Rheumatology 2003; 42: 292-7
10. Klocke R, Cockcroft JR, Taylor GJ, Hall IR, Blake DR. Arterial stiffness and central blood pressure, as determined by pulse wave analysis, in rheumatoid arthritis. Ann Rheum Dis 2003; 62(5):414-418
11. Belch, JJF, McArdle B, Madhok R, McLaughlin K, Forbes CD, Sturrock RD. Decreased plasma fibrinolysis in patients with rheumatoid arthritis. Annals of Rheumatic Diseases 1984; 43 (6): 774-779.
12. Lau CS, McLaren M, Hanslip J, Kerr M, Belch JJF. Abnormal plasma fibrinolysis in patients with rheumatoid arthritis and impaired endothelial fibrinolytic response in those complicated by vasculitis. Annals of Rheumatic Diseases 1993; 52 (9): 643-649
13. McEntegart A, Capell HA, Madhok R, Lowe GDO, Rumley A, Woodward M. Cardiovascular (CVS) Risk factors in an RA population. Rheumatology 2000; 39: 40
14. McLaren M, Alkaabi J, Connacher M, Belch JJ, Valenete E. Activated factor XII in rheumatoid arthritis. Rheumatol. Int. 2002; 22(5): 182-4
15. Douglas KM, Pace AV, Treharne GJ, Saratzis A, Nightingale P, Erb N, Banks MJ, Kitas GD. Excess recurrent cardiac events in rheumatoid arthritis patients with acute coronary syndrome. Ann Rheum Dis 2006; 65(3): 348-53
16. Van Doornum S, Brand C, King B, Sunfararajan V. Increased case fatality rates following a first cardiovascular event in patients with rheumatoid arthritis. Arthritis Rheum. 2006; 54 (7): 2061-8
17. Veldhuijzen van Zanten JJ, Ring C, Caroll D, Kitas GD. Increased CRP in response to acute stress in patients with rheumatoid arthritis. Ann Rheum Dis. 2005; 64(9): 1299-304
18. Ross R. Atherosclerosis - An inflammatory disease. N Eng J Med 1999; 340: 115-26
19. Kitas GD, Banks MJ, Bacon PA. Accelerated Atherosclerosis as a cause of cardiovascular death in RA. Pathogenesis 1998; 1 (2): 73-83
20. Sattar N, McCarey DW, Capell H, McInnes IB. Explaining how "high-grade" systemic inflammation accelerates vascular risk in rheumatoid arthritis. Circulation 2003; 108(24):2957-2963
21. Stevens R, Douglas KMJ, Saratzis A, Kitas GD. Inflammation and atherosclerosis in rheumatoid arthritis. Exp. Rev. Mol. Med. 2005; 7 (7): 1-24
22. Danesh J, Whincup P, Walker M, Lennon L et al. Low grade inflammation and coronary heart disease: prospective study and updated meta-analyses. Br Med J 2000; 321: 199-204
23. Morrow DA, Ridker PM. C-reactive protein, inflammation and coronary risk. Med Clin N Am 2000; 84: 149-61
24. Goodson NJ, Symmons DPM, Scott DG, Bunn D, Lunt M, Silman AJ. Baseline levels of CRP and prediction of death from cardiovascular disease in patients with inflammatory polyarthritis: a ten year follow up study of a primary care based cohort. Arthritis Rheum 2005; 52(8): 2293-9
25. Wallberg-Jonsson S, Johansson H, Ohman ML, Rantapaa-Dahlqvist S. Extent of inflammation predicts cardiovascular disease and overall mortality in seropositive rheumatoid arthritis: a retrospective cohort study from disease onset. J Rheumatol 1999; 26: 2562-71
26. Jacobsson LT, Turesson C, Hanson RL et al. Joint swelling as a predictor of death from cardiovascular disease in a population study of Pima Indians. Arthritis Rheum. 2001; 44: 1170-6
27. Chehata JC, Hassell AB, Clarke SA et al. Mortality in rheumatoid arthritis: relation to single and composite measures of disease activity. Rheumatology 2001; 40: 447-52
28. Choi HK, Herman MA, Seeger JD, Robins JM, Wolfe F. Methotrexate and mortality in patients with rheumatoid arthritis: a prospective study. Lancet 2002; 359: 1173-7
29. Mitchell DM, Spitz PW, Young DY, Bloch DA, McShane DJ, Fries JF. Survival, prognosis, and causes of death in rheumatoid arthritis. Arthritis Rheum 1986; 29: 706-14
30. Lehtinen K, Isomaki H. Intramuscular gold therapy is associated with long survival in patients with rheumatoid arthritis. J Rheumatol 1991; 18: 524-9
31. Krause D, Schleusser B, Herborn G, Rau R. Response to methotrexate treatment is associated with reduced mortality in patients with severe rheumatoid arthritis. Arthritis Rheum 2000; 43: 14-21
32. Landewe RBM, van den Borne BEEM, Breedveld FC, Dijkmans BAC. Methotrexate effects in patients with rheumatoid arthritis with cardiovascular comorbidity. Lancet 2000; 355: 1616-7
33. Erb N, Kitas GD. Homocysteine modulation as a reason for continuous folic acid supplementation in methotrexate-treated rheumatoid arthritis patients. Rheumatology 2001; 40: 715-6
34. Hurliman D. et al. Anti-tumor necrosis factor alpha treatment improves endothelial function in patients with rheumatoid arthritis. Circulation 2002; 106(17): 2184-7
35. Raza K, Banks M, Kitas GD. Reversing myocardial microvascular disease in a patient with rheumatoid arthritis. J Rheumatol 2005; 32(4): 754-6
36. Van Doornum S, McColl G, Wicks IP. Tumour necrosis factor antagonists improve disease activity but not arterial stiffness in rheumatoid arthritis. Rheumatology 2005; 44(11): 1428-32
37. Situnayake RD, Kitas GD. Dyslipidaemia and rheumatoid arthritis. Ann Rheum Dis 1997; 56: 341-2
38. Dessein PH, Joffe BI, Veller MG et al. Traditional and non-traditional cardiovascular risk factors are associated with atherosclerosis in rheumatoid arthritis. J Rheumatol 2005; 32(3): 435-42
39. Alkaabi JK, Ho M, Levison R, Pullar T, Morley KD, Belch JJF. The prevalence of macrovascular disease in Rheumatoid Arthritis (RA) Rheumatology 2000; 39: 39
40. Goodson NJ, Silman AJ, Pattison DL, Lunt M, Bunn D, Luben R, Day N, Khaw KT, Symmons DP. Traditional cardiovascular risk factors measured prior to the onset of inflammatory polyarthritis. Rheumatology 2004; 43(6): 731-6
41. Erb N, Pace AV, Douglas KJM, Banks M, Kitas GD. Risk assessment for coronary heart disease in rheumatoid arthritis and osteoarthritis. Scand. J. Rheumatol. 2004; 33: 293-299
42. Treharne GJ, Hale ED, Lyons AC, Booth DA, Banks M, Erb N, Douglas KM, Mitton DL, Kitas GD. Cardiovascular disease and psychological morbidity among rheumatoid arthritis patients. Rheumatology 2005; 44(2): 241-6
43. HOPE study investigators. Effects of an ACE inhibitor, ramipril, on cardiovascular events in high risk patients. N Eng J Med 2000; 342:145-53
44. Bacon PA, Kitas GD. Rheumatoid Arthritis, Vasculitis and Arteriosclerosis. In: Atherosclerosis and Autoimmunity; Shoenfeld Y, Harats D, Wick G (eds); Elsevier Science BV 2001; 301-13
45. Adhiyaman V, Asghar M, Oke A, White AD, Shah IU. Nephrotoxicity in the elderly due to co-prescription of angiotensin converting enzyme inhibitors and nonsteroidal anti-inflammatory drugs.J R Soc Med. 2001; 94(10):512-4
46. Downs JR, Clearfield M, Weis S et al for the AFCAPS/TexCAPS Research Group. Primary prevention of acute coronary events with lovastatin in men and women with average cholesterol levels. JAMA 1998; 279: 1612-22
47. Shepherd J, Cobbe SJ, Ford I et al for the West of Scotland Coronary Prevention Study Group. Prevention of coronary heart disease with pravastatin in men with hypercholesterolaemia. N Eng J Med 1995; 333: 1701-7
48. Scandinavian Simvastatin Survival Study Group. Randomised trial of cholesterol lowering in 4444 patients with coronary heart disease. The Scandinavian Simvastatin Survival Study (4S). Lancet 1994; 344: 1383-9
49. Sacks FM, Pfeffer MA, Moye LA et al. The effect of pravastatin on coronary events after myocardial infarction in patients with average cholesterol levels. N Eng J Med 1996; 335: 1001-9
50. Long-term Intervention with Pravastatin in Ischaemic Disease (LIPID) Study Group. Prevention of cardiovascular events and death with pravastatin in patients with coronary heart disease and a broad range of initial cholesterol levels. N Eng J Med 1998; 339: 1349-57
51. Plehn JF, Davis BR, Sacks FM et al. Reduction of stroke incidence after myocardial infarction with pravastatin. The cholesterol and recurrent events (CARE) study. Circulation 1999; 99: 216-23
52. Leung BP, Sattar N, Crilly A et al. A novel anti-inflammatory role for simvastatin in inflammatory arthritis. J. Immunol. 2003; 420: 78-84
53. Blake GJ, Ridker PM. Are statins anti-inflammatory? Curr Control Trials Cardiovasc Med 2000; 1: 161-5
54. Palinski W, Napoli C. Unravelling pleiotropic effects of statins on plaque rupture. Arterioscl Thromb Vasc Biol 2002; 22: 1745-50
55. Kwak B, Mulhaupt F, Myit S, Mach F. Statins as a newly recognised type of immunomodulator. Nat Med 2000; 6: 1399-1402
56. Weitz-Schmidt G. Statins as anti-inflammatory agents. TRENDS in Pharmacol Sci 2002; 23 (10): 482­486
57. Gotto AM Jr. Antioxidants, statins and atherosclerosis. J Am Coll Cardiol. 2003; 41 (7): 1205-10
58. Kitas GD, Sattar N. The potential role of statins in the treatment of rheumatoid arthritis. J. R. Coll. Physicians Edinb. 2005; 35: 309-16
59. Douglas KMJ, Sattar N, Kitas GD. Potential role of statins and PPARs in rheumatoid arthritis. Future Rheumatology 2006; 1(2): 259-274
60. McCarey DW, McInnes IB, Madhok R, Hampson R et al. Trial of atorvastatin in rheumatoid arthritis (TARA): double-blind, randomised, placebo-controlled trial. Lancet 2004; 363: 2015-21
61. Colhoun HM, Thomason MJ, Mackness MI, Maton SM et al. Design of the collaborative atorvastatin diabetes study (CARDS) in patients with type 2 diabetes. Diabetic Medicine 2002; 19: 201-11 (results just presented in abstract form)
62. Koren MJ, Hunninghake DB & the ALLIANCE Investigators. Clinical outcomes in managed care patients with coronary heart disease treated aggressively in lipid-lowering disease management clinics. The ALLIANCE study. J. Am. Coll. Cardiol. 2004; 44: 1772-9
63. Cannon CP, Braunwald E, McCabe BS, Rader DJ et al. Comparison of intensive and moderate lipid lowering with statins after acute coronary syndromes. N. Eng. J. Med. 2004; 350
64. Kinlay S, Timms T, Clark M, Karam C et al. Comparison of effect of Intensive lipid lowering with atorvastatin to less intensive lowering with lovastatin on C-reactive protein in patients with stable angina pectoris and inducible myocardial ischemia. Am. J. Cardiol. 2002; 89: 1205-7
65. van Wissen S, Trip MD, Smilde TJ, de Graaf J et al. Differential hs-CRP reduction in patients with familial hypercholesterolaemia treated with aggressive or conventional statin therapy. Atherosclerosis 2002; 165: 361-6
66. Topol EJ. Intensive statin therapy - A sea change in cardiovascular prevention. N. Eng. J. Med. 2004; 350
67. Williams B, Poulter NR, Brown MJ, Davis M et al. British Hypertension Society guidelines for hypertension management 2004 (BHS-IV): summary. Brit Med J 2004; 328: 634-40
68. Schwartz GG, Olsson AG, Ezekowitz MD, Ganz P et al. Effects of atorvastatin on early recurrent ischemic events in acute coronary syndromes. The MIRACL study: a randomized controlled trial. JAMA 2001; 285: 1711-8
69. Van Doornum S, McColl G, Wicks IP. Atorvastatin reduces arterial stiffness in patients with rheumatoid arthritis. Ann Rheum Dis. 2004; 63(12): 1571-5
70. Klareskog L, Hamsten A. Statins in rheumatoid arthritis: two birds with one stone? Lancet 2004; 363: 2011-1
71. The American Rheumatism Association 1987 revised criteria for the classification of rheumatoid arthritis. Arthritis Rheum 1988;31:315-24
72. Kirwan JR, Reeback J. Stanford health assessment questionnaire modified to assess disability in British patients with rheumatoid arthritis. Br J Rheumatol 1986;25:206-9
73. EuroQol Group. EuroQol—a new facility for the measurement of health-related quality of life. Health Policy 1990;16:199-208.
74. Prevoo MLL, Van’t Hof MA, Kuper HH et al. Modified disease activity scores that include twenty-eight joint counts Arthritis Rheum 1995; 38: 44-8
75. Witney AG, Treharne GJ, Tavakoli M, Lyons AC, Vincent K, Scott DL, Kitas GD. The relationship of medical, demographic and psychosocial factors to direct and indirect health utility instruments in rheumatoid arthritis. Rheumatology 2006; 45(8): 975-81
76. Shephard J, Blauw GJ, Murphy MB, Cobbe SJ et al. The design of a Prospective Study of Pravastatin in the Elderly at Risk (PROSPER)
77. Thygesen, K, Alpert, JS and White, HD. Universal Definition of Myocardial Infarction. Eur Heart

A1.1 Study Hypothesis

- Atorvastatin is more effective than placebo as adjuvant therapy for the control of disease activity in patients with RA
- Atorvastatin is more effective than placebo as adjuvant therapy in slowing radiographic damage and the long-term decline in physical function in patients with RA
- RNA samples will allow gene expression profiling studies in RA. Blood samples collected from this patient population would facilitate research in a broader array gene expression profiling approach and would allow assessment of differential expression of genes without a priori information.

A1.2 Study Design

A multicentre, randomised, double blind, placebo-controlled trial of atorvastatin 40mg once daily for control of disease activity in patients with RA aged >50 years or with >10 years of RA disease duration (if under 50 years of age). This is a sub-study of the main TRACE RA trial which explores the influence of atorvastatin on cardiovascular endpoints.

A total of 326 patients will be required and each patient will be followed for two years (with respect to the sub-study but will also be followed up until the end of the cardiovascular/main study).

The inclusion and exclusion criteria for the disease activity sub-study are similar to the main study with the express proviso that all patients entering the sub-study must have a baseline DAS28 score of >3.2.

A1.3 Proposed Duration of Treatment, Follow up and Frequency of Intervention

Patients participating in this substudy will have an additional nurse global assessment and be asked to provide a blood sample for mRNA, serum and plasma extraction at baseline and month 6 of their trial visit. (As outlined in Appendix 2)

Patients will also have the following assessment in Months 6, 12, and 24:

28 tender and swollen joint counts Patient global assessment Nurse global assessment ESR and/or CRP

A1.4 Proposed Sample Size

A sample size of 326 patients is required to provide 80% power at the 5% significance rate to detect a difference in EULAR response rate between statin treated and the placebo arms. This assumes a 20% response rate in the placebo arm and 20% loss to follow-up during the 2 years of the sub-study.

A1.5 Planned Analysis

All patients randomised in the trial will be included in the analysis, irrespective of whether the study drug was discontinued (ITT analysis). EULAR response rates in the two arms will be compared using odds ratios with 95% confidence intervals and adjusting for any baseline differences between the groups. Rheumatology efficacy outcomes will be recorded at 6, 12 and 24 months visits. For safety outcomes, logistic regression models will be used for analysis with treatment allocation together with the factors used for stratification. Any further baseline variables can be incorporated into the model if necessary to adjust for baseline imbalances. Two-sided p values of <0.05 will be considered significant.

A1.6 Planned Sub-group Analysis

Sub-group analyses will be carried out with respect to: gender, age group (<65 vs. >65 years), RA disease duration, rheumatoid factor status and anti TNF therapy (if number is sufficient).

A1.7 Proposed Frequency of Analysis

No interim analysis is planned for TRACE RA DAS. Safety data will be continuously monitored by the independent DMC.

A1.8 Proposed Collection and Use of blood samples

A repository committee will be created by the Trial Steering Committee (TSC) to oversee work relating to the blood samples and to consider requests from other academic and clinical research organisations for the blood samples. All requests from academic or pharmaceutical organisations to use the samples would be submitted to an ethics committee for regulatory approval.

A2.1 Background

Rheumatoid Arthritis (RA) was previously considered to be a benign, controllable disease with a reasonably good prognosis in the majority of patients. It is now known that it can be a severe, progressive disorder associated with a premature mortality. Patients with RA have a 5-year mortality rate similar to that of patients post myocardial infarction (MI), with triple coronary vessel disease or with neoplastic disease, RA should be viewed as an urgent medical problem requiring immediate intervention. Despite this, research targeted to cardiovascular disease (CVD), the main cause of death in RA, was ignored for many years. In RA life span is shortened by approximately 15-20% from the date of onset of the illness.

Between 34-40% of the excess deaths are from cardiovascular causes. Various studies have given Standard Mortality Ratios for CVD in RA of between 1.51 to 5.25, where the lower estimate is for MI only and the largest includes heart failure. The TRACE RA study addresses the clinical problem of enhanced CV mortality in this group of patients via a randomised controlled trial (RCT) of Atorvastatin versus placebo in 3,808 RA patients. This study is funded by the arc and the British Heart Foundation and will provide novel data on the beneficial, or otherwise, effects of a statin on CV events and mortality in RA. The aim of the trial is to establish whether atorvastatin will protect patients with RA aged >50 years or with >10 years’ duration of RA from fatal and non-fatal atherosclerotic events. 3,808 subjects are to be recruited from 60 centres over 18m, and undergo treatment with atorvastatin or placebo for 5 years after recruitment of the last patient. CV events and mortality is the primary endpoint. A nested sub-study (TRACE RA-DAS) has also been designed to determine whether atorvastatin, used in conjunction with standard DMARD therapy, will provide added arthritis control in patients with moderately or severely active RA. TRACE RA-DAS will recruit 326 patients and will run for 2 years. The primary endpoint is EULAR ‘moderate’ or ‘good’ response using the DAS 28.

However, such a trial, where the study population is large and well characterised, also provides unique opportunities for major studies, particularly those where DNA/RNA and biomarkers can be used to shed light on disease susceptibility, outcome and drug responsiveness. Familial, twin, admixture and migration studies all indicate a substantial contribution of genetic factors in the aetiology of this common complex disorder. Substantial advances in gene identification are now being made: linkage scans have highlighted consistently replicated regions harbouring susceptibility genes and a growing number of substantiated susceptibility variants have been defined. Further progress in definition and characterisation of novel genes will require a large community-based RA DNA resource, enriched by the availability of detailed long-term clinical follow-up.

This resource will allow: study of the modest relative risks expected of many susceptibility variants (by comparison with ‘normal’ populations), replication of other resources, measurement of the population attributable risk of susceptibility variants, evaluation of potential gene/gene and gene/environment interactions, definition of complication-related susceptibility variants, and exploration of genetic effects on treatment response. There is, therefore, a clear case to be made for establishing such a resource on the basis of genetic studies alone.

In addition the study of inflammation and CV disorders is evolving, in particular biomarkers for disease progression and events are becoming available, and more will be uncovered. A resource of banked serum and plasma from a well-phenotyped large population has the potential to answer questions relating to these novel mechanisms in a rapid, powered and convincing fashion. These resources will also provide a substrate for the ‘omic’ disciplines. Mircroarray analysis of the transcriptome is now a mature field and continues to provide insight into prognosis in many disease areas, and is likely to be highly informative in the study of drug response in clinical trials. The collection of plasma and serum ion the proposed Biobank will also provide a substrate for the emerging proteomic and metabonomic studies.

A2.2 Study Aims

- To develop a DNA repository for consenting patients with rheumatoid arthritis (RA) enrolled in the TRACE RA study, in centres that agree to provide such samples.
- To develop an RNA repository for gene expression profiling from consenting patients in the TRACE RA­DAS substudy at 2 time points (baseline and 6months).
- To develop a plasma and serum repository for the above patients from centres where such blood preparation is possible, and the centres agree to provide such samples.
- To devise and complete peer-reviewed projects on the above samples using the phenotypic and outcome data collected in the TRACE RA trial.
- To take advantage of established population-based record-linkage capability, to allow longitudinal tracking of the subjects, thereby enabling investigation of the relationship between susceptibility genotypes and pertinent clinical features including characteristics at diagnosis, response to treatment, development of RA complications, vascular disease and survival.

To develop an infrastructure surrounding the collection, storage and use of these samples which concerns itself with ethics, consent, privacy and collaboration, to protect the rights of the patients whilst maintaining the highest standards of clinical research

A2.3 Methodology:

Informed consent and blood will be collected from 5,350 patients with RA that are participating in the main TRACE RA study.

1. Subjects:

The subjects will be those enrolled into the TRACE RA study (inclusion and exclusion criteria described in TRACE RA protocol). More than 4000 RA patients will be enrolled in up to 120 centres. The RNA (see below) will be sampled from the 326 subjects in the TRACE RA-DAS substudy, who give consent for this sampling. As it may not be possible to collect serum and plasma from all centres due to centrifuge and freezer availability, only DNA samples may be collected from all TRACE RA trial participants, with as many as possible of these giving also plasma and serum. Although this protocol defines a 3,808 population this caveat applies throughout.

1. Sample handling and DNA extraction:

The following blood samples will be taken from each subject who gives consent. DNA will be collected from all consenting subjects, and serum and plasma where possible from as many subjects as can be managed: One 4.5mls citrate sample (for plasma), two 10mls EDTA (for plasma), two 10mls clotted (for serum), two 10mls EDTA (for DNA, immediately stored, without spinning, at -20/-80C OR sent on ice within 48hrs). The same samples will be taken from patients in the DAS substudy at baseline, with the addition of 4 x 2.5 mls samples for RNA sampling. Serum and plasma (same volumes as above) and RNA will also be taken at 6months from patients in the TRACE RA-DAS substudy who have given consent for sampling. Serum and plasma will be prepared, aliquoted and frozen at the collection site in an appropriately timely fashion where possible, as this is optimum for sample preparation and costs. Where whole blood is to be stored the Biobank co-ordinator will work with the centre to obtain ratification of temporary sample storage at the centre under the Tissue Act. Those samples which are frozen will be transported in batches on dry ice via Courier in specially prepared packages routinely used by the group for sample transfer. Transfer to Ninewells Hospital will be followed by appropriate further sample preparation, and storage in alarmed secure freezers. DNA will be prepared from 10mls of blood using Promega Wizard blood DNA preparation procedure (with one sample remaining as back up) and all samples will be quantified by fluorescence dye techniques to maximise standardisation of concentrations, an essential prerequisite to robust, quality high throughput genotyping. Finally, each sample will be tested in a multiplex PCR reaction that will include an SRY marker (a Y-specific amplicon, as a check on identity integrity) and one autosomal marker (such as beta-actin) (to confirm DNA quality). DNA quantification, normalisation, dilution and aliquoting will be performed using an Xiril X100 liquid handling robot (available in Dundee), and stored at -30^0^c in bar coded replicate 96 and 384 well plates. Importantly, DNA samples will be bar-coded and the robot will confirm the identity of each sample and track the progress of each sample to all daughter plates. Normalized DNA will be distributed to Pfizer and to Manchester in 96 well format.

1. RNA extraction: Whole blood samples will be taken in four 2.5mls PAXgene RNA stabilization vacutainers (BD/Qiagen) from each of the 326 subjects in the TRACE RA-DAS substudy on two occasions ie time zero and at 6m, to correlate with clinical disease activity measures. RNA will be prepared in a 96 well format using QiAMP PAXgene 96 blood RNA system. This will be run as a semi-automated process using a Xiril X100 liquid handling robot. RNA will be assessed for integrity by lab-on-a-chip technology using an Agilent 2100 Bioanalyzer.

A2.4 Projects and Statistics:

A Repository Committee will be set up to oversee the work relating to these samples, and to consider requests from other centres for DNA. This committee will consist of the Chief and Lead Investigators, the AR UK selected Trial Steering Committee chairman (Prof Gordon Lowe, University of Glasgow), amongst others, including patient representation. The potential for study of these samples is huge and 3 exemplar projects have been appended. These are merely examples of many possible projects which will be developed by the Repository Steering Committee, in collaboration with others. All requests from academic or pharmaceutical organisations to use the samples would be submitted to an ethics committee for regulatory approval.

The mainstay of analyses based on this resource will be using conventional genetic/serum/plasma statistical methodology, encompassing a range of different analytical approaches and study objectives (including for example, single-locus and haplotype-based studies; biological and positional candidate analyses; replication studies for associations uncovered in other (smaller) populations; logistic regression analyses seeking to dissect LD structure and define aetiological variants; and gene-gene interactions). In addition, the associated longitudinal clinical data will permit analyses of the relationship between genotypes of interest and pertinent outcomes including complication rates; treatment response; survival/mortality; and disease progression. These data will be analysed using multivariate regression and Cox regression methods, including algorithms for repeated measures when appropriate. The trial statistician is Dr Peter Nightingale, (University of Birmingham, The Wellcome Trust Clinical Research Facility), who has ensured the appropriate powering of TRACE RA, and who is involved in the day to day statistical issues of the Trial.

The mainstay of analyses based on this resource will be using conventional genetic/serum/plasma statistical methodology, encompassing a range of different analytical approaches and study objectives (including for example, single-locus and haplotype-based studies; biological and positional candidate analyses; replication studies for associations uncovered in other (smaller) populations; logistic regression analyses seeking to dissect LD structure and define aetiological variants; and gene-gene interactions). In addition, the associated longitudinal clinical data will permit analyses of the relationship between genotypes of interest and pertinent outcomes including complication rates; treatment response; survival/mortality; and disease progression. These data will be analysed using multivariate regression and Cox regression methods, including algorithms for repeated measures when appropriate.

A2.5 Facilities and Experience available for the study:

Dundee’s Ninewells Hospital is uniquely placed to act as the physical Repository; due to its experience in such studies to date. Dr Colin Palmer and Professor Jill Belch have extensive experience in sample preparation for these types of studies.

These include grants from TENOVUS to pilot endothelial studies in a large population of patients with diabetes, the Raynaud’s and Scleroderma Association to link the development of carotid vascular disease to certain genotypes in Systemic sclerosis, from the Wellcome Trust UK Case/control study of type 2 diabetes, where all subjects (n=15000) are from Tayside. Tayside is also the principal centre for the recruitment and sample handling for Generation Scotland, with 10,000 individuals to be targeted within the next 2 years, growing to 50,000 Scotland wide by the end of 2011. The robotics and software developed for these large studies will easily accommodate the currently proposed sample throughput.

POPADAD, a Scotland wide 8 year CV mortality trial (n= 1,320) also has Ninewells (and the Dundee applicants) as its core laboratory for sample collection. In addition we have recently developed a “High Volume” genotyping workflow to complement our “high throughput” affymetrix and Illumina systems, where we can cheaply and efficiently genotype 10,000 patient samples for 1 SNP in 2 hours. This system uses both KASPAR indirect allelic discrimination and TAQMAN based allelic discrimination. KASPAR is simpler and much cheaper, but assays that fail on KASPAR will be performed on TAQMAN. Both genotyping techniques are homogenous fluorescent assays that can be performed easily in an automated fashion. Sample reformatting will be performed to 384 well formats on a Xiril liquid handling robot. Reaction

mixtures will be dispensed to 384 using a DEERAC high speed nanodispenser (1-2ul total reaction volumes). PCR will be performed in a H2OBIT ultra-high-throughput thermal cycler (10,000 samples per 2 hour run) and the final genotypes scored using an Applied Biosystem SDS9700. The genotyping project will be analysed, managed and quality controlled using a dedicated genotyping database system (KLUSTERCALLER, KBiosciences).

Standard analytical techniques, in addition to genetic studies, include HPLC-MS, GC-MSMS, ELISA, Spectrophotometry, Affymetrix micro-array, MALDI, proteomics (Prof Mike Ferguson), flow cytometry, (Luminex Bead array) and Western Blotting.

A2.6 Ethical considerations

1. Confidentiality, security, ethics and database management

With the implementation of the new data protection act issues of data security and confidentiality are very much in the public eye, as they should be. The mechanisms employed to ensure appropriate security and confidentiality within Ninewells result from years of deliberation and debate and are continually reviewed and updated in parallel with legal and ethical requirements. The exemplar project for Ninewells is the DARTS project, (Diabetes Audit and Research Tayside). As an example of how TRACE RA samples will be treated using DARTS logistics will be explained below.

Clinical information on DARTS is stored on a Structured Query Language (SQL) database. Clinical information is accessed via a Web server and Web browser. The first level of security on clinical data is that imposed by the NHS-NET network. Although DARTS has a presence on the wider Internet, users from the Internet cannot access the DARTS Web Server located within the confines of NHS-NET. A login screen is presented and communications are all encrypted, and access determined by username and password. All attempts to log-on to DARTS and every action subsequently taken are logged producing audit trails. For DARTS anonymous Genetic research we use all the security and confidentiality mechanisms provided by the Microsoft SQL Server V7 platform.

The Tenovus grant award has allowed us to implement a secure system for anonymous genetic case/control studies. Every patient, GP, general practice and hospital clinic represented on the system is allocated a unique randomly generated identifier intended for the purposes of anonymous research. Researchers are then restricted to ‘views’ of data that use these anonymous identifiers and omit any identifying fields.

Thus, all demographic information is removed except sex, age (in months), and social class (Carstair’s Index). For genetic research, only the nurse performing the fieldwork has access to the patients identifying numbers. This nurse does not have access to any other patient-specific data. In addition, although the system administrator (through necessity) has access to all data on the server, the identity of individual genetic markers is not available to him.

The present information sheet is included in Appendix 3. It has been modified according to MRC guidelines to state that products derived from the sample may be used by other researchers and the commercial sector. It includes specific clauses that allow use of samples for RA and wider medical issues including drug response.

All record linkage studies and issues relating to confidentiality, anonymisation of data and dataset security will come under the aegis of the Health Informatics Centre at the University of Dundee. This has an external scientific Advisory Board (chair Professor Elizabeth Russell, Professor of Epidemiology, University of Aberdeen) that scrutinises all anonymised record linkage research in Tayside according to published standard operating procedures.

1. Administration of the Collection

To maximise the utility of the proposed DNA resource, the collection will be overseen, and access to the resource managed, by the TRACE RA Repository Committee. This steering group would become the custodian of the DNA resource to ensure that maximum benefit is derived from the collection whilst avoiding any unnecessary duplication of genotyping/assaying.

DNA aliquots will be made and master stocks stored in Dundee. Complete plated collections of the DNA (~15ug) will be distributed to the appropriate collaborating centres (see exemplars below ie Manchester). Requests for DNA from external collaborators will be considered. Written applications will be to test specific variants within a gene and standardized data will be required in these requests: data to establish the importance of the gene, the role of the specific variants to be typed eg coding variants, haplotype tagging SNPs etc, details of the assay used for SNPs, preliminary data to give allele frequencies and power calculation.

Permission will be given to exclusively (for one year after permission is granted) test the requested specific SNPS. DNA aliquots may be obtained from Dundee DNA will be given blinded and will include approximately 10% of samples with the most DNA as duplicates to provide a measure of genotyping accuracy. A condition of using the resource will be that all genotyping will be given to the core database so that these data are held centrally; the specific data field however will remain under the custodianship of the scientist performing the study until the data are published. The analysis of the ongoing longitudinal data will be obtained by users of the resource working with the epidemiological team in Dundee. A collaborative framework has been established in the recent SRIF-funded Health Informatics Centre at Ninewells Hospital.

The Health Informatics Centre thus has standard operating procedures that facilitate this collaborative inter-institutional research on anonymised datasets for approved projects. In order to ensure the continuation and the development of this resource, a cost recovery charge will be levied for the supply of DNA and subsequent data analysis to external centres from Dundee/Manchester. Pricing will be determined by the Repository committee based on cost recovery and will be aligned with other UK resources. We would use these funds to support the maintenance and development of the resource at Ninewells beyond the period of this award.

Note: the plasma and serum will be utilised by the TRACE RA Steering Committee as this is a more finite resource.

A2.7 Conclusion

We anticipate great demand both nationally and internationally because, to our knowledge there is no comparable publicly available resource.

It will be especially unique because of the longitudinal follow-up of phenotypic data. This Repository will form the basis of an RA Framingham and provide insight into disease mechanisms, complications and drug responsiveness for many years to come.

APPENDIX 3: SAMPLE PATIENT INFORMATION SHEET, CONSENT FORMS AND GP LETTERS

These are provided as separate documents and will be stored in the master trial file, regional trial unit site files and the centres’ site files.

Please see list of documents as follows (All documents will be approved by MREC):

1. Patient Information Leaflet - Main TRACE RA trial
2. Patient Information Leaflet - TRACE RA BioBank (Genetic sub-study)
3. Patient Information Leaflet & Consent - TRACE RA DAS and DAS Biobank (Disease Activity sub-study)
4. Consent form Main TRACE RA trial and TRACE RA Biobank (Genetic Substudy)
5. Patient Information Leaflet and Consent - DNA Collection Only
6. GP letter - for centres that routinely assess RA patients for 10 year cardiovascular disease risk
7. GP letter - for centres that do not routinely assess RA patients for 10 year cardiovascular disease risk
8. GP referral letter
9. Promotional Leaflet - TRACE RA information to patients
10. Promotional Leaflet - TRACE RA information to investigators

These are provided as separate documents and will be stored in the master trial file, regional trial unit site files and the centres’ site files.

Please see list of documents as follows (All documents will be approved by MREC):

1. Lifestyle questionnaire
2. Health Assessment Questionnaire (HAQ)
3. EQ5D - EuroQol 5 dimensional lifestyle questionnaire
4. Patient Global Assessment (Visual Analogue Score)
5. Nurse Global Assessment (Visual Analogue Score) - (TRACE RA DAS Substudy only)

An independent cardiovascular endpoint classification committee will review cardiovascular events and all deaths and will classify them according to the WHO MONICA method, or alternative of their choice.

Primary Endpoints

3.1 Primary endpoints

TRACE RA (all patients): Primary endpoints

The primary comparison will involve Cox regression analyses of “major vascular events” (defined as: coronary events [i.e. non-fatal myocardial infarction, coronary death or coronary revascularisation]; presumed ischaemic stroke or transient ischaemic attack; any non-coronary revascularisation or any other cardiovascular death excluding both confirmed cerebral haemorrhage [ICD I64-99 in the 10^th^ International Classification of Diseases]) and non-coronary cardiac death [ICD I00-I15 and I26-I52] during the scheduled treatment period among all those allocated atorvastatin tablets versus all those allocated placebo tablets (i.e. “intention-to-treat” comparisons).

TRACE RA-DAS (sub-study only):

EULAR moderate or good response based on DAS 28 at 6 months

1. Secondary and tertiary endpoints

- - 1. TRACE RA secondary endpoints

Components of the primary endpoint separately:

Coronary events;

Presumed ischaemic stroke or TIA;

Any non-coronary arterial revascularisation;

Any other cardiovascular death excluding both confirmed cerebral haemorrhage [ICD I64-99 in the 10^th^ International Classification of Diseases]) and non-coronary cardiac death [ICD I00-I15 and I26-I52]

TRACE RA tertiary endpoints

Total and cause-specific mortality (coronary, other vascular and non-vascular death separately) Hospitalisations for various other causes

Statin safety-related outcomes (persistent elevation of ALT or AST; myopathy (defined as muscle symptoms plus CK>10 x upper limit normal)

Differences in lipid levels during follow-up in a random sample Functional outcome assessed by HAQ and EQ5D

Allowance for multiple hypothesis testing in these analyses will be made using the “Bonferroni” correction.

TRACE RA-DAS secondary endpoints

DMARD (Disease Modifying and Anti-Rheumatic Drugs) changes DAS 28 at month 12 and 24.

Functional outcome assessed by HAQ and EQ5D at month 6, 12 and 24.

A5.1 FATAL ENDPOINTS

A5.1.1 Coronary heart disease death

Death certificate or equivalent documentation with consistent or underlying or immediate cause plus one of the following:

1. Preterminal hospitalisation with acute myocardial infarction (see definitions below).
2. Previous documented angina or myocardial infarction when no other cause other than atherosclerotic coronary heart disease death could be ascribed as the cause of death.
3. Sudden, unexpected cardiac death, involving cardiac arrest, often with symptoms of myocardial ischaemia, and accompanied by presumed new ST segment

elevation, or new LBBB, and/or evidence of fresh coronary artery thrombosis by coronary arteriography, but death occurring before blood samples could be obtained, or at a time before the appearance of cardiac biomarkers in the blood.

1. Autopsy evidence of acute coronary arterial thrombosis and/or acute myocardial infarction.

*A5.1.2 Suspect coronary heart disease death*

Death certificate or equivalent documentation with consistent underlying or immediate cause but neither adequate preterminal documentation of the event nor previous diagnosis of atherosclerotic coronary heart disease.

*A5.1.3 Fatal Presumed ischaemic Stroke*

Death certificate or equivalent documentation with consistent or underlying or immediate cause plus either:

1. Preterminal diagnosis of stroke (see definitions below).
2. Autopsy evidence of cerebral infarction

*A5.1.5 Other atherosclerotic cardiovascular death (e.g. ruptured aortic aneurysm, mesenteric infarction, peripheral arterial disease):*

Death certificate or equivalent documentation with consistent underlying or immediate cause and adequate preterminal documentation of the event.

Note: Preterminal documentation and/or information may include hospitalisation for, or diagnosis of, a vascular-related illness for a previous event other than the terminal event.

A5.1.6 Non-cardiovascular death:

Death certificate or equivalent documentation with diagnosis consistent with preterminal documentation and/or information.

Death certificate only

Note: When no formal written documentation is available, verbal information from relative and/or witness will be admissible and should be recorded on the appropriate study forms.

A5.2 NON-FATAL EVENTS

*A5.2.1 Acute myocardial infarction (Universal definition of myocardial infarction, Thygesen et al Eur Heart J 2007;28:2525-8)*

Detection of rise and/or fall of cardiac biomarkers (preferably troponin) with at least one value above the 99^th^ percentile of the upper reference limit (URL) together with evidence of myocardial ischaemia with at least one of the following:

Symptoms of ischaemia

ECG changes indicative of new ischaemia [new ST-T changes or new left bundle branch block (LBBB)] (see glossary)

Development of pathological Q waves in the ECG (see glossary)

Imaging evidence of new loss of viable myocardium or new regional wall motion abnormality.

Elevations of troponin for other causes, in the absence of evidence of myocardial ischaemia, will not be classified as myocardial infarction (see glossary).

For percutaneous coronary interventions (PCI) in patients with normal baseline troponin values, elevations of cardiac biomarkers above the 99^th^ percentile URL are indicative of peri- procedural myocardial necrosis. By convention, increases of biomarkers greater than 3 x 99^th^ percentile URL have been designated as defining PCI-related myocardial infarction. A subtype related to a documented stent thrombosis is recognised.

For coronary artery bypass grafting (CABG) in patients with normal baseline troponin values, elevations of cardiac biomarkers above the 99^th^ percentile URL are indicative of peri- procedural myocardial necrosis. By convention, increases of biomarkers greater than 5x99th percentile URL plus either new pathological Q waves or new LBBB, or angiographically documented new graft or native coronary artery occlusion, or imaging evidence of new loss of viable myocardium have been designated as defining CABG-related myocardial infarction.

*A5.2.3 Coronary Revascularisation*

Coronary artery bypass graft surgery (CABG) or percutaneous coronary intervention (angioplasty, atherectomy, laser ablation or stenting or any newly introduced invasive method for the management of coronary artery disease).

Note: Acceptable documentation will include hospital discharge letters, operation notes or other clinical correspondence documenting performance & date of procedure.

*A5.2.4 Stroke (any event that meets the criteria listed below for one of the following 3 categories of stroke):*

Ischaemic stroke (one of the following conditions must be met):

Rapid onset of focal neurological deficit lasting >24 hours or leading to death plus evidence from neuroimaging (CT or MRI) showing cerebral/cerebellar infarction or no abnormality, or post-mortem examination showing cerebral and/or cerebellar infarction. Rapid onset of global neurologic deficit (e.g. coma) lasting >24 hours or leading to death plus evidence from neuroimaging showing cerebral/cerebellar infarction, or post-mortem examination showing infarction.

Focal neurologic deficit (mode of onset uncertain) lasting >24 hours or leading to death plus evidence from neuroimaging showing cerebral/cerebellar infarction, or post-mortem examination showing infarction.

Primary intercerebral and/or cerebellar haemorrhage (one of the following conditions must be met):

Rapid onset of focal neurologic deficit lasting >24 hours or leading to death plus evidence from neuroimaging or post-mortem examination showing primary intracerebral and/or cerebellar haemorrhage.

Rapid onset of global neurologic deficit (e.g. coma) lasting >24 hours or leading to death, plus evidence from neuroimaging or post-mortem examination showing primary intracerebral and cerebellar haemorrhage.

Focal neurologic deficit (mode of onset uncertain) lasting >24 hours or leading to death, plus evidence from neuroimaging or post-mortem examination showing primary intracerebral and/or cerebellar haemorrhage.

Stroke- mechanism unknown (one of the following conditions must be met):

Rapid onset of focal neurologic deficit lasting >24 hours or leading to death, without neuroimaging or post-mortem data available.

Rapid onset of global neurologic deficit (e.g. coma) lasting >24 hours or leading to death, without neuroimaging or post-mortem data available.

Focal neurologic deficit (mode of onset uncertain) lasting >24 hours or leading to death, without neuroimaging or post-mortem data available.

Note: The following conditions will be excluded from the defined endpoint of stroke as outlined above:

Primary intracerebral haemorrhage Primary subarachnoid haemorrhage Subdural or extradural haematoma Traumatic intracerebral haemorrhage

Neurologic deficit due to major metabolic or haemodynamic disturbance Venous sinus thrombosis Cerebral tumour

Cerebral embolism secondary to infective endocarditis

Any other causes of neurological deficit which, in the opinion of the committee, are not primarily cardiovascular in origin

*A5.2.5 Carotid revascularisation*

Coronary endarterectomy or percutaneous carotid intervention (angioplasty, atherectomy, laser ablation or stenting or any newly introduced invasive method for the management of carotid artery disease).

Note: Acceptable documentation will include hospital discharge letters, operation notes or other clinical correspondence documenting performance of procedure & date.

*A5.2.6 Non-fatal peripheral atherosclerotic events*

Hospital-verified acute peripheral arterial ischaemic events. The diagnosis is based on symptoms, clinical findings and/or appropriate radiological investigations.

*A5.2.7 Peripheral arterial revascularisation*

Any one of :

Repair of aneurysm or peripheral arterial revascularisation by open surgical or percutaneous techniques (angioplasty, atherectomy, laser ablation or stenting or any newly introduced invasive method for the management of aortic or peripheral arterial disease).

Above-ankle amputation for critical limb ischaemia

Chemical or surgical sympathectomy for atherosclerotic peripheral arterial disease.

Note: Acceptable documentation will include hospital discharge letters, operation notes or other clinical correspondence documenting performance of procedure & date.

*A5.2.9 Emergency Hospitalisation for angina*

Evidence of emergency hospital admission with chest pain considered due to angina pectoris, plus previous history of documented atherosclerotic coronary artery disease, or documented ischaemic ECG changes during admission, but not meeting criteria for acute myocardial infarction.

A5.3 SECONDARY ENDPOINTS

*A5.3.1 All-cause mortality*

Sum of all cardiovascular plus non-cardiovascular deaths.

*A5.3.2 Fatal peripheral atherosclerotic events*

Death occurring within 28 days from the onset of hospital-verified acute peripheral arterial atherosclerotic events. The diagnosis is based on symptoms, clinical findings and/or appropriate radiological investigations.

The Endpoints Committee will be responsible for the classification of all possible study endpoints. The Committee will receive all baseline and end of^7^ trial electrocardiograms showing serial changes, information regarding domiciliary visits or hospitalisation associated with possible endpoints, and information on all deaths.

A5.4 GLOSSARY

*A5.4.1 Clinical classification of different types of myocardial infarction*

Type 1

Spontaneous myocardial infarction related to ischaemia due to a primary coronary event such as plaque erosion and/or rupture, fissuring, or dissection Type 2

Myocardial infarction secondary to ischaemia due to either increased oxygen demand or decreased supply, e.g. coronary artery spasm, coronary embolism, anaemia, arrhythmias, hypertension, or hypotension Type 3

Sudden unexpected cardiac death, including cardiac arrest, often with symptoms suggestive of myocardial ischaemia, accompanied by presumably new STelevation, or new LBBB, or evidence of fresh thrombus in a coronary artery by angiography and/or at autopsy, but death occurring before blood samples could be obtained, or at a time before the appearance of cardiac biomarkers in the blood Type 4a

Myocardial infarction associated with PCI Type 4b

Myocardial infarction associated with stent thrombosis as documented by angiography or at autopsy Type 5

Myocardial infarction associated with CABG

*A5.4.2 Causes of elevations of troponin in the absence of overt ischemic heart disease*

Cardiac contusion, or other trauma including surgery, ablation, pacing, etc.

Congestive heart failure—acute and chronic

Aortic dissection

Aortic valve disease

Hypertrophic cardiomyopathy

Tachy- or bradyarrhythmias, or heart block

Apical ballooning syndrome

Rhabdomyolysis with cardiac injury

Pulmonary embolism, severe pulmonary hypertension

Renal failure

Acute neurological disease, including stroke or subarachnoid haemorrhage Infiltrative diseases, e.g. amyloidosis, haemochromatosis, sarcoidosis, and scleroderma Inflammatory diseases, e.g. myocarditis or myocardial extension of endo-/pericarditis Drug toxicity or toxins

Critically ill patients, especially with respiratory failure or sepsis Burns, especially if affecting .30% of body surface area Extreme exertion

*A5.4.3 ECG manifestations of acute myocardial ischaemia (in absence of LVH and LBBB)*

ST elevation

New ST elevation at the J-point in two contiguous leads with the cut-off points: >0.2 mV in men or >0.15 mV in women in leads V2-V3 and/or >0.1 mV in other leads.

ST depression and T-wave changes

New horizontal or down-sloping ST depression >0.05 mV in two contiguous leads; and/or T inversion >0.1 mV in two contiguous leads with prominent R-wave or R/S ratio >1.

*A5.4.4 ECG changes associated with prior myocardial infarction*

Any Q-wave in leads V2-V3 >0.02 s or Qs complex in leads V2 and V3.

Q-wave >0.03 s and >0.1 mV deep or QS complex in leads I, II, aVL, aVF, or V4-V6 in any two leads of a contiguous lead grouping (I, aVL,V6; V4-V6; II, III, and aVF).

R-wave >0.04 s in V1-V2 and R/S >1 with a concordant positive T-wave in the absence of a conduction defect.

The same criteria are used for supplemental leads V7-V9, and for the Cabrera frontal plane lead grouping.

Definitions of non-coronary arterial revascularisations: Non-cardiac procedures

non coronary arterial surgery/intervention unspecified

arterial surgery unspecified

non coronary angioplasty +/- stent

fem-pop bypass/leg artery bypass

popliteal or femoral or iliac aneurysm repair

aortic aneurysm repair or stent

carotid surgery

cerebral artery aneurysm surgery or clipping

arterial graft reconstruction/excision (not dialysis access)

leg artery angioplasty +/- stent

renal artery angioplasty +/- stent

carotid angioplasty +/- stent

non coronary angiogram

angiogram of leg/femoral angiogram

renal artery angiogram

carotid angiogram or arch aortagram

Embolectomy

Amputations

amputation of leg

above knee amputation

below knee amputation

amputation of foot

amputation of toe

amputation of arm

Clinical laboratory safety parameters are shown below. Haematology and biochemistry will be performed at the local laboratories. All of the safety parameters are subject to routine regular monitoring for safety of the disease-modifying anti-rheumatic drugs (DMARDs). Such monitoring is required as part of routine clinical practice at intervals ranging between 2 weeks and 3 months for all but one DMARDs (the antimalarials). All patients will be questioned about muscle symptoms at each follow-up visit and if new and significant CK will be measured. Only serious adverse events will be recorded. Randomisation codes will be kept securely by participating pharmacists. If deemed necessary by the local investigator the randomisation code can be broken by contacting the local pharmacy.

Abnormalities of haematological and biochemical parameters in this population may be either due to DMARDs or the study drug. The decision of attribution will be left to the managing local investigator.

The study drug should be stopped if:

- Patient has new and significant muscle pains and CK>10xULN,Patient has new and significant muscle pains and ALT/AST >2xULN and CK between 3-10xULN and elevation persists upon re­testing.
- Myopathy is diagnosed (muscle pain or weakness AND CK >10 times ULN)
- Elevation of CK to >5 times ULN, persisting on retesting 1 week later, should also lead to discontinuation of study medication.

Atorvastatin is generally well-tolerated. Adverse reactions have usually been mild and transient. Less than 2% of patients were discontinued from clinical trials due to side effects attributed to Atorvastatin.

The ‘Summary of Product Characteristics’ about Atorvastatin will be filed in the TRACE RA site file. Information about Atorvastatin can be found on the electronics medicines compendium section of the ([www.emc.medicines.org.uk](http://www.emc.medicines.org.uk)) website and detailed information about the side effects of the drug is also available in the British National Formulary.

Clinical Adverse Experiences (Please see ‘Summary of Product Characteristics’ for more details)

The most frequent (1% or more) adverse effects associated with Atorvastatin therapy, in patients participating in controlled clinical studies were:

Psychiatric disorders - Insomnia.

Nervous System disorders - Headache

Gastrointestinal disorders - Abdominal pain, dyspepsia, nausea, flatulence, constipation, diarrhoea Musculoskeletal and Connective Tissue Disorders: Myalgia

General Disorders and Administration Site Conditions:

- Asthenia
- Elevated serum ALT levels have been reported in patients receiving Atorvastatin.
- Elevated serum CPK levels > 3 times upper normal (ULN) occurred in 2.5 % of patients on Atorvastatin compared with 3.1% with other HMG-CoA reductase inhibitors in clinical trials.

[Additional adverse events that have been reported in atorvastatin clinical trials are categorised below according to system organ class and frequency. Frequencies are defined as: very common (>10%), common (>1% and <10%), uncommon (>0.1% and <1%), rare (>0.01% and <0.1%) and very rare (0.01%)].

| **Organ/System Disorder** | **Event** | **Frequency** |
| --- | --- | --- |
| Metabolism & Nutrition | Anorexia  Hypoglycaemia  Hyperglycaemia | Uncommon  Very rare  Very rare |
| Nervous system | Dizziness  Paraesthesia  Peripheral neuropathy  Dysgeusia | Common  Uncommon  Uncommon  Very rare |
| Psychiatric | Amnesia  Depression | Uncommon  Uncommon |
| Blood and Lymphatic System | Thrombocytopenia | Uncommon |
| Immune System | Allergic reaction (including anaphylaxis | Common |
| Gastrointestinal | Anorexia  Vomiting  Pancreatitis | Uncommon  Uncommon  Uncommon |
| Hepatobiliary | Hepatitis  Cholestatic jaundice  Hepatic failure | Rare  Rare  Very rare |
| Skin & Subcutaneous Tissue | Alopecia  Urticaria  Pruritus  Rash | Uncommon  Uncommon  Common  Common |
| Musculoskeletal and Connective Tissue | Muscle cramps  Myositis  Rhabdomyolysis  Myopathy  Arthralgia  Tendon rupture | Uncommon  Rare  Rare  Uncommon  Common  Very rare |
| Eye Disorders | Visual disturbance | Very rare |
| Ear and Labyrinth Disorders | Tinnitus  Hearing Loss | Uncommon  Very rare |
| Reproductive system | Sexual dysfunction  Gynaecomastia | Uncommon  Very rare |
| General disorders | Chest pain  Back pain  Fatigue  Angina  Malaise  Weight gain  Peripheral oedema  Angioneurotic oedema  Interstitial lung disease | Common  Common  Common  Common  Uncommon  Uncommon  Rare  Very rare  Very rare |

APPENDIX 8: POWER CALCULATION AND OTHER STATISTICAL CONSIDERATIONS

Table 1

Total number of patients with events and total number of events required for Cox regression (80% power at 5% significance level)

Reduction in Annual Event Rate (after adjustment for non-compliance) Number of patients with events Number of events*

| 20% | 631 | 671 |
| --- | --- | --- |
| 21% | 565 | 597 |
| 22% | 509 | 534 |
| 23% | 460 | 481 |
| 24% | 41 7 | 434 |
| 25% | 379 | 393 |
| 26% | 346 | 358 |
| 27% | 31 7 | 327 |
| 28% | 291 | 299 |

*assuming independence of events

Table 2

Expected total number of events for various combinations of annual event rate and patient-years of follow-up

Patient-Years

Annual event rate^[[2]](#footnote-2)^

Follow-up

|  | 1.3% | 1.4% | 1.5% | 1.6% | 1.7% | 1.8% |
| --- | --- | --- | --- | --- | --- | --- |
| 25000 | 325 | 350 | 375 | 400 | 425 | 450 |
| 26000 | 338 | 364 | 390 | 416 | 442 | 468 |
| 27000 | 351 | 378 | 405 | 432 | 459 | 486 |
| 28000 | 364 | 392 | 420 | 448 | 476 | 504 |

APPENDIX 9: COMPOSITION AND ROLE OF TRIAL STEERING COMMITTEE AND DATA MONITORING COMMITTEE

A9.1 TRACE RA Trial Steering Committee (TSC) – Composition

The TSC membership should be limited and include an independent Chairman (not involved directly with the trial other than as a member of the TSC), two or more other independent expert members and the Chief Investigator. Where possible the membership should include a lay/consumer representative. Caroline Dore, the Arthritis Research UK’s Senior Statistician, who is based at the MRC Clinical Trials Unit, should be invited to all meetings, in the role of observer. The trial manager, trial statistician etc should attend meetings as appropriate. Observers from Arthritis Research UK and Host Institution should be invited to all meetings.

Chairman: Professor Gordon Lowe, Emeritus Professor, Glasgow University Independent Members:

Professor Jane Armitage, Professor of Clinical Trials and Epidemiology & Honorary Consultant, CTSU, University of Oxford

Professor Keith Fox, Professor of Cardiology, University of Edinburgh Centre for Cardiovascular Science Professor Dorian Haskard, Director, Eric Bywaters Centre for Vascular Inflammation, Imperial College London

AR UK member:

Ms Caroline Dore, Senior Clinical Trial Statistician, AR UK Lay member:

Ms Ailsa Bosworth, Chief Executive, National Rheumatoid Arthritis Society Investigators:

Professor George Kitas, Consultant Rheumatologist, Dudley Group of Hospitals NHS Trust Professor Jill Belch, Professor of Cardiovascular and Inflammation Medicine & Honorary Consultant Physician, Institute of Cardiovascular Research, Ninewells Hospital

Professor Deborah Symmons, Professor of Rheumatology and Musculoskeletal Epidemiology, University of Manchester

Secretary: Dr Hawys Williams, Clinical Trials Manager, University of Manchester

A9.2 TRACE RA Trial Steering Committee (TSC) -Role of the Trial Steering Committee:

It is Arthritis Research UK’s policy that a Trial Steering Committee (TSC) should be set up for each of its multi-centre trials with the following terms of reference:

Terms of Reference:

To monitor and supervise the progress of the trial towards its interim and overall objectives including viewing the unblinded lipid differences during the study.

1. To review at regular intervals relevant information from other sources (e.g. other related trials)
2. To consider the recommendations of the Data Monitoring Committee (DMC)
3. In light of 1, 2 & 3, to inform Arthritis Research UK on the progress of the trial
4. To advise Arthritis Research UK on publicity and the presentation of all aspects of the trial.

The TSC provides overall supervision for the trial, and advice to the CI, Arthritis Research UK and the Host Institution on all aspects. The first meeting of the TSC should be held before recruitment to the trial begins, in order for the TSC to approve the protocol. Thereafter, the TSC should meet at least annually. The TSC should ensure that there are no major deviations from the trial protocol. The CI should call meetings of the TSC when there are any matters arising from the conduct or management of the trial that might require their advice.

A9.3 Data Monitoring Committee (DMC) - Composition

It is the sponsors’ responsibility to decide whether a DMC is needed for a particular trial and if so to appoint one. The sponsors have agreed the composition of the DMC outlined below. The frequency with which the DMC meets will be dependent on the needs of the trial. The Chief Investigator (CI) should submit a detailed plan for the interim analysis before the trial commences. The plan must satisfy members of the DMC. Communication between the CI and the DMC chair is encouraged but should not bypass the TSC Chair.

The CI and the Chair of the TSC will agree with the DMC Chair a timely mechanism for reporting to the DMC. With the help of the trial statistician, the CI must provide blinded data, in strict confidence, to the DMCas frequently as the members of the sub-group request. Serious unexpected suspected adverse reactions must be reported to the lead clinician of the DMC and chairperson of the relevant multi-centre research ethics committee immediately. If appropriate, the MHRA (Medicines and Healthcare products Regulatory Agency) must also be informed.

Chairman: Professor Michael Frenneaux, Regius Professor of Medicine, University of Aberdeen

Members:

Dr Christopher Edwards, Consultant Rheumatologist, University of Southampton Dr Jonathan Emberson, Senior Statistician, University of Oxford Clinical Service Unit Professor Deborah Bax, Consultant Physician in Rheumatology, Clinical Director of Specialised Medicine, Royal Hallamshire Hospital, Honorary Professor of Rheumatology (University of Sheffield)

A9.4 Data Monitoring Committee (DMC) – Role

The DMC is the only body involved in the trial that has access to the unblinded comparative data. The role of the DMC is to monitor these data and make recommendations to the TSC on whether the trial should continue. Membership of the DMC should be completely independent of the CI, TSC and Host institution. The first meeting of the DMC should be held before recruitment to the trial begins, in order for the DMC to approve the protocol and ensure that appropriate arrangements have been made for review of the accumulating data and the results of any interim analyses.

Terms of Reference: DMC

1. To set up and maintain direct communication with the CI and Chair of the TSC. The Chair of the TSC should be made aware of all communications between the CI and DMC sub-group.
2. To receive a copy of the trial protocol and plans for interim analysis prior to commencement of the trial, or, in the case of the first wave of trials, as early as possible.
3. To receive reports (as per template in Appendix 3) during the trial at intervals agreed with the TSC and CI. It would be expected that these would be 6 monthly in the first year, and no less frequent than 12 monthly after that.
4. If interim analysis of the trial data is not planned in the protocol the sub-group should determine whether interim analysis should be undertaken
5. To consider data from interim analyses, unblinded if considered appropriate, plus any additional safety issues for the trial and relevant information from the template and other sources
6. In the light of 3, 4 & 5, and ensuring that ethical considerations are of prime importance, to report to the TSC and recommend on the continuation of the trial.

# DAS - TRACE RA DAS REQUIREMENTS

**APPENDIX 10: TRIAL EVALUATION SCHEMA**

| Trial Investigations | Visit 1 | | Visit 2 | Visit 3 | Visit 4 | | Visit 5 | Visit 6 | | Visit 7, 9, 11, 13, | Visit 8, 10, 12, 14, 16 |
| --- | --- | --- | --- | --- | --- | --- | --- | --- | --- | --- | --- |
|  | Baseline | | Month 3 | Month 6 | Month 12 | | Month 18 | Month 24 | | Month 30, 42, 54, 66,78 | Month 48, 60 , 72, 84 |
|  | ALL | DAS | ALL | DAS | ALL | DAS | ALL | ALL | DAS | ALL | ALL |
| Inclusion/Exclusion Criteria Assessments | ✓ | # |  |  |  |  |  |  |  |  |  |
| Written Informed Consent | ✓ | # |  |  |  |  |  |  |  |  |  |
| Height, Weight, BP | ✓ |  |  |  |  |  |  |  |  |  |  |
| Medical History | ✓ |  |  |  |  |  |  |  |  |  |  |
| Concomitant Medication | ✓ |  | ✓ |  | ✓ |  | ✓ | ✓ |  | ✓ | ✓ |
| ESR/CRP, RhF/anti-CCP, LFTs at baseline and LFTs at follow up visit | ✓ |  |  |  | ✓ |  | ✓ | ✓ |  | ✓ | ✓ |
| Research DNA blood sample (BioBank) | ✓ |  |  |  |  |  |  |  |  |  |  |
| Research serum and plasma (BioBank) ^[[3]](#footnote-3)^ | ✓ |  |  |  |  |  |  |  |  |  |  |
| Research RNA sample (DAS) |  | # |  | # |  |  |  |  |  |  |  |
| Research serum and plasma (DAS) |  | # |  | # |  |  |  |  |  |  |  |
| Discussion of CVD risks | ✓ |  |  |  |  |  |  |  |  |  |  |
| HAQ | ✓ |  |  | # | ✓ |  |  | ✓ |  |  | ✓ |
| EQ5D | ✓ |  |  | # | ✓ |  |  | ✓ |  |  | ✓ |
| Lifestyle Questionnaire | ✓ |  |  |  |  |  |  |  |  |  |  |
| DAS 28 Tender & Swollen joint count | ✓ |  |  | # |  | # |  |  | # |  |  |
| Patient Global Assessment (VAS) | ✓ |  |  | # |  | # |  |  | # |  |  |
| Nurse Global Assessment (VAS) |  | # |  | # |  | # |  |  | # |  |  |
| Check for Serious Adverse events &Cardiovascular events |  |  | ✓ |  | ✓ |  | ✓ | ✓ |  | ✓ | ✓ |
| Drug compliance |  |  | ✓ |  | ✓ |  | ✓ | ✓ |  | ✓ | ✓ |

APPENDIX 11: AMENDMENTS TO THE STUDY PROTOCOL

Appendix 11.1: The following changes have been incorporated into Version 2 (dated 27^th^ July 2007) of the protocol

- The title page footer updated to new date and version
- Changes to Trial Management Team: Trial Manager changed from Ms Sumitra Smith to Dr Hawys Williams. Regional trials co-ordinators added (Ms Rebecca Storey and Mrs Shobna Vasishta)
- Changes to Trial steering committee details: Secretary changed from Ms Sumitra Smith to Dr Hawys Williams
- Endpoints Committee: Chairman (Professor Stuart Cobbe) appointed and details added.
- TRACE RA DAS substudy: Following advice from the Trial Statistician, it was decided that X-rays would be omitted from Version 2 of the trial protocol. This was based on a Power Calculation identifiying that no changes would be detectable by performing X-rays at baseline and 24 months.
- TRACE RA DAS substudy: changes to sample volumes collected at baseline and at 6 months to reflect collaboration with Pfizer to create TRACE RA DAS Biobank
- TRACE RA Biobank: changes to sample volumes collected at baseline to reflect collaboration with Pfizer to create TRACE RA Biobank
- Section 5: Eligibility. Adequate Contraception. Additional information added with regards to methods of adequate contraception.
- Section 6.2.3, Follow up visits for TRACE RA participants: Addition of Liver function test (LFT) to be included as part of 3 month drug compliance/safety visit.
- Main trial schema: Amended to clarify that patients withdrawn from the trial should continue to be followed up annually. This includes patients following a CV event.
- Section 6.1. Patient selection and informed consent: Clarification that centres do not have to fax registration forms prior to randomisation, as original protocol version implied. Centres are requested to fax the registration forms after randomisation.
- Section 6:2.1 Baseline/randomisation visit: Clarification that routine blood test results can be used up to 6 weeks prior to randomisation for trial screening.
- Section 7. Study withdrawal: Clarification that patients withdrawn due to experiencing a cardiovascular event will continue for annual follow up visits as well as flagging for mortality.
- Section 8. Drug supplies and labelling: Change of ‘Cardinal’ to ‘Catalent’ to reflect supplier’s name change.
- Appendix 3. Sample Patient Information Sheet, Consent Forms and GP Letters: following additional documents added:

o Patient Information Leaflet and Consent Form - DNA collection only o GP letter for centres that routinely assess for 10 year cardiovascular disease risk o GP letter for centres that DO NOT routinely assess for 10 year cardiovascular disease risk o GP referral letter

- Appendix 5. Definition of Cardiovascular Endpoints: This section has been re-written by the Chair of the Endpoints Committee to provide further clarification of the endpoints and to adopt the Universal Definition of Myocardial Infarction (Thygesen et al; 2007).
- Appendix 7. Information about Atorvastatin: This section has been amended to incorporate new information published by Pfizer (Summary of Product Characteristics dated 10 December 2007) and also the Drug Safety Update publishes by the MHRA (February 2008) reporting that additional side- effects (sleep disturbances, memory loss, sexual dysfunction, depression, and interstitial lung disease) have been recognised with statins.

Appendix 11.3: The following changes have been incorporated into Version 4 (dated 3^rd^ March 2010) of the protocol

- Section 7. Study withdrawal: Clarification that patients can re-commence trial medication and insertion of guidance on blood tests required prior to re-commencing and thereafter.
- Chief Investigator and statistician Protocol approval and Principal Investigator declaration pages inserted.
- Appendix 2.3 TRACE RA Biobank, A2.3 Methodology b. Sample handling and DNA extraction: Amendment to clarify Biobank blood samples taken as part DAS substudy.
- DAS substudy: DAS score entry criteria reduced from >4.4 to >3.2
- Addition of Pfizer Representative to membership of the Biobank Repository Committee
- Amendment to Professor Lowe’s professional title to reflect his retirement from medical practice in 2009.
- Amendment to start and end dates for trial.

Appendix 11.4: The following changes have been incorporated into Version 4.1 (dated 6^th^ May 2010)

of the protocol

- Section 6.2.2. Allocation of patients to trial treatment arm: following changes to the DAS substudy entry criteria and consultation with the TSC, the requirement for separate labelling of DAS substudy medication was no longer deemed necessary. This is as the number of patients randomised to each arm is expected to be equal if centres as DAS substudy patients will be randomised sequentially. This section of the protocol was amended to reflect this.
- Section 9.5. Changes to causality assessment - addition of ‘related’ as one of the criteria and removal of ‘unlikely to be related’.
- Entire document updated with change of name of Arthritis Research Campaign to Arthritis Research UK to reflect change of name of charity (March 2010)
- Appendix 9: Composition and role of Trial Steering Committee and Data Monitoring Committee: Composition altered to reflect changes in DMC membership.
- Appendix I & II amended in light of Biobank samples no longer being automatically shared with Pfizer.

Appendix 11.5: The following changes have been incorporated into Version 5 (dated 17^th^ December 2010) of the protocol

Summary of Key Points:

- Trial recruitment has been extended to March 2014 with the last follow-up appointment being in March 2016, or until a sufficient number of events has accrued to provide statistical power whichever is the earlier. Recruitment target amended to more than 4000 patients.
- Number of participating centres increased from 100 to 120
- New Power calculations (based on observed recruitment and number of events) and subsequent increase in required sample size.
- Revision of Endpoints to include non-coronary revascularisations, TIA and exclude angina and haemorrhagic stroke
- Pharmacovigilance procedures amended to exclude reporting of adverse events unless an adverse reaction is deemed to have a reasonable causal relationship with atorvastatin. Procedures also amended to provide further guidance on management of patients with new and significant muscle pains.
- Case Report form and blood tests have been simplified
- Patient Information Leaflet - Main trial amended in light of protocol amendment and format designed to make it more visually appealing.
- Patients’ addresses to be collected retrospectively and prospectively
- Co-Sponsor (University Of Manchester) representative amended from Dr Karen Shaw to Prof.

Nalin Thakker

Detailed Description of Protocol Changes:

- Trial Contacts, Page 2: Trial contacts amended in light of Miss Donna Watson and Miss Donna Kempson leaving the trial team and Dr Emma Knox being appointed as Trial Co-ordinator (Manchester CTU)
- TSC Committee Membership, Page 3: Removal of Prof Cobbe as member of TSC, following his retirement. Amendment to Jane Armitage’s title from Dr to Professor
- Trial Schema, Page 6: Amendment to trial schema in accordance with changes in study visits (please see below).
- Section 3.1 (Primary Endpoints), page 11: Primary endpoints amended to include non-coronary revascularisations, TIA and exclude angina and haemorrhagic stroke
- Section 3.2 (Secondary and Tertiary Endpoints), page 11: Secondary endpoints refined to focus on coronary events and Tertiary endpoints added.
- Section 4 (Study Organisation), page 12: Number of patients randomised increased from 3800 to at least 4000 and number of participating centres increased from 100 to 120.
- Section 5 (Eligibility), page15: List of contra-indicated drugs updated according to the SmPC and data from other statin trials.
- Section 6.1: (Patient selection and informed consent) page 15: Addition of pre-printed patient detail logs to procedures to facilitate tracking of data.
- Section 6.2.1 (Baseline/randomisation visit) page 15: Removal of family history of cardiovascular disease, baseline ECG and measurement of waist circumference. ESR/CRP test results can be used within the last 6 weeks if patient’s is stable and treatment unchanged. RhF +/or anti-CCP can also be used from previous timepoints. Removal of routine haematology and biochemistry blood tests.

Removal of random glucose blood test and CK measurement. Removal of collection of data for ONS flagging for mortality of ineligible and eligible but not participating in the trial.

- Section 6.2.3 (Follow up visits for TRACE RA participants), page 16: Previously patients were asked to attend

clinic for annual appointments with prescriptions every 6 months. The protocol has been amended to request identical 6 monthly follow-up, which can be conducted by telephone if necessary

or in their home if required. ECG taken at the end of trial removed. The management of patients whom indicate presence of new and significant muscle symptoms has been revised.

- Section 6.4: (End of trial) page 19: Addition of plan to continue recruiting until March 2014 with last follow-up in March 2016, or until a sufficient number of events has accrued to provide statistical power.
- Section 7 (Discontinuation of study medication), page 20: This section was previously entitled ‘Study withdrawal’ and has been revised to provide clarity that as the trial will be analysed on an intention to treat analysis, patients are asked to continue to be followed up to the end of trial, regardless of whether they remain on the study medication. Patients are also able to recommence on study medication at any time provided liver function tests (ALT/AST) are within the screening limits.
- Section 8.2 (Supply of study medication to centres) page 20: All study medication packs for new patients will comprise of 6 monthly supply, instead of previously supply them with 3 month supply at baseline and 3 month visit. The 3 month study visit will remain.
- Section 9.1 (Cardiovascular outcomes and reporting of cardiovascular outcomes) page 21: A single SAE form will be used for the reporting of cardiovascular endpoints and other SAEs.
- Section 9.2 (Serious adverse events) page 21: Elective rheumatological procedures will now be reported as serious adverse events.
- Section 9.3 (Adverse events) page 21: Unless leading to discontinuation of study treatment, adverse events are not required to be recorded on the Case Report Form. Procedures for management of patients presenting with new and significant muscle pains added.
- Section 9.6 (Reporting of SAEs, SARs and SUSARs to Pfizer) page 22: Reporting requirement of SAEs to Pfizer added.
- Section 9.7 (Recording and reporting of all SAE/SARs) page 23: Flow diagram amended in light of changes in previous sections.
- Section 10.1 (Sample size) page 24: The sample size has been re-calculated by the trial statistician based on recruitment rates in the first 3 years and observed event rates during this period.
- Section 10.2 (Planned recruitment rate) page 24: Number of recruiting centres increased from 100 to 120, with required number of patients recruited by March 2014.
- Section 10.3 (Compliance): page 26: Compliance to study treatment will be measured by asking the patient instead of tablet count.
- Section 11.1 (Assessment of efficacy outcomes) page 26: Addition of procedure for censoring of patients from study medication if they experience a cardiovascular endpoint or develop condition for which statin therapy clearly indicated.
- Section 11.2 (Assessment of safety) page 26: Clarification of procedures for patients presenting with new and significant muscle pains.
- Section 12.7 (Financial matters) page 30: Details of UKCRN adoption of trial and its substudies added.
- Section A2.3 (Biobank Methodology) page 40: Increase in number of patients recruited to Biobank from 3808 to more than 4000.
- Section A3 (Sample Patient Information Sheet, Consent Forms and GP letters) page 44: Removal of Patient Information Leaflet: ONS Flagging, GP letter for ONS flagging consent from non-participants, GP letter for withdrawn patients.
- Section A5 (Definitions of cardiovascular endpoints) page 46-51: Primary endpoints amended to include non-coronary revascularisations, TIA and exclude angina and haemorrhagic stroke. Secondary endpoints refined to focus on coronary events and Tertiary endpoints added. Definitions of non­coronary revascularisations added.
- Section A6 (Safety Outcomes) page 52: Actions to be taken on presentation new and significant muscle pains and elevation CK added.
- Section A8 (Power calculations and other statistical calculations) page 55: Amended in light of statistical remodelling of trial statistics based on observed recruitment and event rate to date.
- Section A9.1 (TSC Committee Composition), page 56: Removal of Prof Cobbe as member of TSC, following his retirement. Amendment to Jane Armitage’s title from Dr to Professor
- Section A10 (Trial Evaluation Schema) page 58: Amended in light of changes to previous sections of protocol (i.e removal of baseline and end of trial ECGs, removal of baseline routine haematology and biochemistry, removal of measurement of waist circumference and family history of cardiovascular disease, all patients to be followed up every 6 months etc.

Section 10.8: Deletion of p values: Independent DMC enquired if a p value of <0.0005 was valid or if this was a typing error."As stated in section 10.8 of the trial protocol there is no formal stopping rule for this study. The p value of <0.0005 was supposed to be a guide as to when the trial should definitely be discontinued: it was not intended that p values > 0.0005 should be used as justification for continuing the trial. It was assumed that the DMC would consider all the available evidence when making such a decision. Rather than changing this value to <0.005, we would prefer to remove the whole sentence from section 10.8, as it is obviously open to misinterpretation."

- Appendix 9: Composition and role of Trial Steering Committee and Data Monitoring Committee: Composition altered to reflect changes in representatives.

Appendix 11.2: The following changes have been incorporated into Version 3 (dated 1^st^ August

2008) of the protocol.

- The title page footer updated to new date and version
- Changes to Trial Team: Project Assistant changed from Ms Nicola Dale to Ms Donna Kempson.
- Data Monitoring Committee: Professor Michael Frenneaux added.
- Endpoints Committee: Professor David Stott, Professor Roger Sturrock and Professor Peter Macfarlane added.


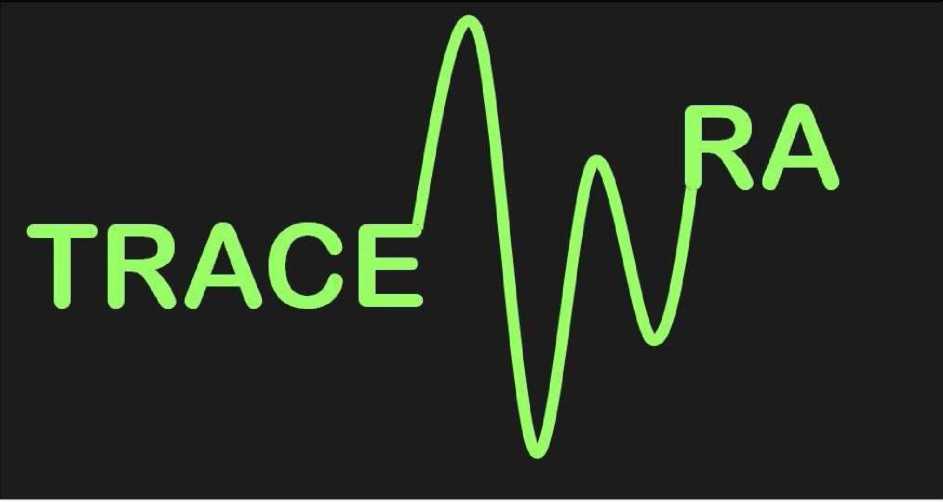


TRial of Atorvastatin for the primary prevention of Cardiovascular Events in Rheumatoid ArthritisSupplementary_Methods_2

**ORIGINAL PROTOCOL**

TRACE RA is supported by the Arthritis Research Campaign and the British Heart Foundation

The trial is co-sponsored by The University of Manchester and Dudley Group of Hospitals NHS Trust

MREC ref no.: 06/Q1704/171 EudraCT no.: 2006-006032-22 ISRCTN: 41829447

**TRIAL CONTACTS**

**Chief Investigator:**

Professor George Kitas Dudley Group of Hospitals NHS Trust, Russells Hall Hospital, Dudley, DY1 2HQ Tel: 01384 244842 Fax: 01384 244808 Email: [g.d.kitas@bham.ac.uk](mailto:g.d.kitas@bham.ac.uk)

PrinPrincipal investigators:

| Professor Deborah Symmons |  | Professor Jill Belch |
| --- | --- | --- |
| ARC Epidemiology Unit University of Manchester Stopford Building, Oxford Road Manchester, M13 9PL |  | Institute of Cardiovascular Research Ninewells Hospital & Medical School Dundee, DD1 4HN |
| Tel: 0161 275 5044 Fax: 0161 275 5043 Email: [deborah.symmons@manchester.ac.uk](mailto:deborah.symmons@manchester.ac.uk) |  | Tel: 01382 632457 Fax: 01382 632333 Email: [j.j.f.belch@dundee.ac.uk](mailto:j.j.f.belch@dundee.ac.uk) |
| Clinical Trial Manager: | | Clinical Trials Assistant: |
| Ms Sumitra Smith |  | Ms Nicola Dale |
| ARC Epidemiology Unit University of Manchester Stopford Building, Oxford Road Manchester, M13 9PL |  | ARC Epidemiology Unit University of Manchester Stopford Building, Oxford Road Manchester, M13 9PL |
| Tel: 0161 275 5639 Fax: 0161 275 5043 Email: [sumitra.smith@manchester.ac.uk](mailto:sumitra.smith@manchester.ac.uk) |  | Tel: 0161 275 3229 Fax: 0161 275 5043 Email: nicola. dale@manchester. ac.uk |

Regional Trials Co-ordinators:

| TBA |  | TBA |
| --- | --- | --- |
| Dudley Group of Hospitals NHS Trust, |  | Institute of Cardiovascular Research |
| Russells Hall Hospital, |  | Ninewells Hospital |
| Dudley, DY1 2HQ |  | & Medical School |
|  |  | Dundee, DD1 4HN |

Statistician:

Dr Peter Nightingale

University of Birmingham The Wellcome Trust Clinical Research Facility First Floor, Blue Zone The Queen Elizabeth Hospital Edgbaston, Birmingham, B15 2TH

Tel: 0121 4721311(Ext.2586)

Email: [p.g.nightingale@bham.ac.uk](mailto:p.g.nightingale@bham.ac.uk)

SENIOR INVESTIGATORS

Dr Ian Bruce, Reader in Rheumatology, Central Manchester & Manchester Children’s University Hospital NHS Trust

Prof. Paul Durrington, Professor of Medicine, Central Manchester & Manchester Children’s University Hospital NHS Trust

Prof. Iain McInnes, Professor of Experimental Medicine, Glasgow Royal Infirmary

Dr Peter Nightingale, Trial Statistician, Wellcome Trust Clinical Research Facility, Birmingham

Prof. Naveed Sattar, Professor of Metabolic Medicine, Glasgow Royal Infirmary

Dr Deva Situnayake, Consultant Rheumatologist, City Hospital, Birmingham

Prof. Allan Struthers, Professor of Clinical Pharmacology, University of Dundee

TRIAL STEERING COMMITTEE

Chairman: Professor Gordon Lowe, Professor of Vascular Medicine, Glasgow Royal Infirmary Independent Members:

Dr Jane Armitage, Senior Research Fellow & Honorary Consultant, CTSU, University of Oxford Professor Keith Fox, Professor of Cardiology, University of Edinburgh Centre for Cardiovascular Science Professor Dorian Haskard, Director, Eric Bywaters Centre for Vascular Inflammation, Imperial College London

ARC member:

Ms Caroline Dore, Senior Clinical Trial Statistician, ARC Lay member:

Ms Ailsa Bosworth, Chief Executive, National Rheumatoid Arthritis Society Investigators:

Professor George Kitas, Consultant Rheumatologist, Dudley Group of Hospitals NHS Trust

Professor Jill Belch, Professor of Cardiovascular and Inflammation Medicine & Honorary Consultant

Physician, Institute of Cardiovascular Research, Ninewells Hospital

Professor Deborah Symmons, Consultant Rheumatologist, University of Manchester

Secretary: Ms Sumitra Smith, Clinical Trials Manager, University of Manchester

DATA MONITORING COMMITTEE

Chairman: Professor Howard Bird, Professor of Pharmacology and Rheumatology, University of Leeds Members:

Dr Christopher Edwards, Consultant Rheumatologist, University of Southampton Dr Hazel Inskip, Senior Scientist, Deputy Director of MRC Epidemiology Resource Centre

TABLE OF CONTENTS Page

| Trial Schema | | 6 |
| --- | --- | --- |
|  | |  |
| 1. | Introduction | 7 |
|  | |  |
| 2. | Background & Rationale |  |
|  | 2.1 Background | 8 |
|  | 2.2 Rationale | 9 |
|  | |  |
| 3. | Trial Design |  |
|  | 3.1 Primary Endpoints | 11 |
|  | 3.2 Secondary Endpoints | 11 |
|  | 3.3 Trial Intervention | 11 |
|  | |  |
| 4. | Study Organisation | 12 |
|  | |  |
| 5. | Eligibility Criteria | 13 |
|  | |  |
| 6. | Trial Procedures |  |
|  | 6.1 Patient accrual & Written Informed Consent | 15 |
|  | 6.2 Trial Investigations | 15 |
|  | 6.3 Procedure for unblinding | 17 |
|  | 6.4 End of Trial | 17 |
|  | |  |
| 7. | Study Withdrawal | 18 |
|  | |  |
| 8. | Drug Supplies & Labelling |  |
|  | 8.1 Packaging & labelling of study medication | 18 |
|  | 8.2 Supply of study medication to centres | 18 |
|  | |  |
| 9. | Pharmacovigilance |  |
|  | 9.1 Cardiovascular outcomes/events reporting | 19 |
|  | 9.2 Adverse events | 19 |
|  | 9.3 Adverse Reactions | 19 |
|  | 9.4 Unexpected Adverse Reactions | 20 |
|  | 9.5 Causality | 20 |
|  | 9.6 Serious Adverse Events/Reactions (SAEs/SARs) | 20 |
|  | 9.7 Suspected Unexpected Serious Adverse Reactions (SUSARS) | 20 |
|  | 9.8 Recording & Reporting of all SAEs/SARs | 21 |
|  | 9.9 Follow-Up of SAEs/SUSARs | 21 |
|  | |  |
| 10. Statistical Considerations | |  |
|  | 10.1 Sample size | 22 |
|  | 10.2 Planned recruitment rate | 22 |
|  | 10.3 Compliance to trial drug | 22 |
|  | 10.4 Loss to follow-up | 22 |
|  | 10.5 Planned analysis | 22 |
|  | 10.6 Procedure for accounting for missing data | 23 |
|  | 10.7 Planned sub-group analysis | 23 |
|  | 10.8 Interim analysis & its frequency | 23 |
|  | 10.9 Economic analysis | 23 |
|  | |  |
| 11. | Assessment of Efficacy & Safety |  |
|  | 11.1 Assessment of Efficacy | 24 |
|  | 11.2 Assessment of Safety | 24 |

| 12. Research Governance   1. Trial administration and logistics 2. Compliance to Protocol 3. Good Clinical Practice 4. Data acquisition & Monitoring 5. Data handling and record keeping 6. Archiving 7. Financial matters 8. Ethical considerations 9. Publication Policy | 25   1. 26 27 27   27   1. 28 28 |
| --- | --- |
|  |  |
| 13. Dissemination of Results |  |
| 13.1 Informing trial participants | 29 |
| 13.2 Expected value of results | 29 |
|  |  |
| 14. Confidentiality & Liability |  |
| 14.1 Patient confidentiality | 30 |
| 14.2 Liability/Indemnity/Insurance | 30 |
|  |  |
| References | 31 |
|  |  |
| Appendix 1: Sub-study 1: TRACE RA DAS sub-study | 34 |
| Appendix 2: Sub-study 2: TRACE RA BioBank sub-study | 35 |
| Appendix 3: Patient information Leaflets, consent forms & GP letters | 39 |
| Appendix 4: Patient lifestyle questionnaire, HAQ & EQ5D | 39 |
| Appendix 5: Definition of Endpoints | 40 |
| Appendix 6: Safety Outcomes | 43 |
| Appendix 7: List of reactions to Atorvastatin | 44 |
| Appendix 8: Power Calculations & other statistical considerations | 45 |
| Appendix 9: Composition & Role of the TSC & DMC | 46 |
| Appendix 10: Trial Evaluations Schema | 48 |

**TRACE RA TRIAL SCHEMA**


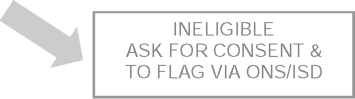
No indication for a statin to be prescribed

Up to 4 weeks later

Existing indication for a statin to be prescribed

REVIEW OF MEDICAL RECORDS TO ASSESS ELIGIBILITY

INFORMED CONSENT


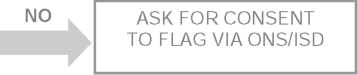
BASELINE BLOOD TESTS ECG, BP, SMOKING STATUS, HAQ, EQ5D HEIGHT, WEIGHT LIFESTYLE FACTORS CONCOMITANT MEDICATION FAMILY HISTORY 28 JOINT COUNT GLOBAL ASSESSMENTS OTHER RELEVANT INFO

BASELINE

VISIT

BASELINE

VISIT

Re-check eligibility

RANDOMISATION


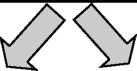
PLACEBO

DRUG


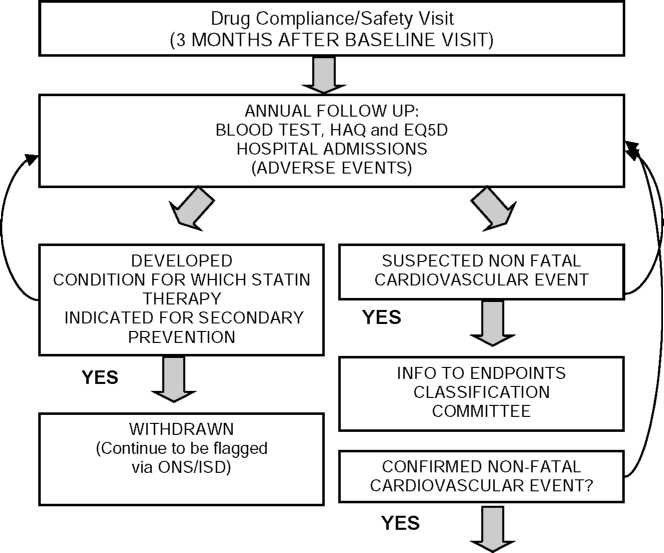


WITHDRAWN (Continue to be flagged via ONS/ISD)

1. **INTRODUCTION**

Rheumatoid arthritis (RA) is associated with increased mortality from cardiovascular disease (CVD). This is thought to be due to accelerated atherosclerotic coronary heart disease (CHD), possibly occurring due to a combination of systemic inflammation augmenting an adverse cardiovascular risk profile.

Statins have a proven beneficial effect in reducing CVD events and mortality in at-risk populations, mostly due to their cholesterol-lowering properties, but also possibly through anti-inflammatory and immunomodulatory effects. Whether such benefit occurs in a high-grade inflammatory condition such as RA remains unknown, because such patients have been systematically excluded from previous statin trials.

This prospective, 5-year, multi-centre, randomised, double blind, placebo-controlled trial will assess the hypothesis that atorvastatin is more effective than placebo in the primary prevention of cardiovascular events in patients with RA*. All patients will receive lifestyle modification advice. Patients already receiving or requiring statin therapy for secondary prevention or whose managing physician thinks they should be on a statin for an existing indication will be excluded.

A nested sub-study (TRACE RA-DAS) will investigate the hypothesis that atorvastatin is more effective than placebo as adjunctive therapy in reducing RA disease activity. Patients enrolled in TRACE RA-DAS will have moderately or severely active disease at the time that they are recruited to the main study.

The main trial (and TRACE RA-DAS) provides a unique opportunity to create a bio bank of appropriately timed DNA, RNA, plasma and serum specimens (TRACE RA-BioBank) that would allow assessment of important supplementary hypotheses in smaller sub-studies. These will, for example, investigate the hypothesis that environmental risk factors for RA and cardiovascular disease overlap and it is likely that there are shared genetic risk factors for the onset and progression of both conditions. A detailed analysis of the molecular markers associated with atorvastatin response in RA and in particular for investigation of inflammatory markers in RA at the level of the genome will also be possible. Whereas the creation of the bio-bank forms part of the current proposal, such sub-studies will be subject to detailed protocol formation and separate ethics approval in the future.

1. Background and Rationale
   1. Trial Background
      1. Cardiovascular disease mortality (CVD) and co-morbidity in RA

Rheumatoid arthritis (RA) affects about 0.8% of the adult population in the UK [1]. It is associated with significant disability and most of the efforts of the scientific community have concentrated on controlling inflammatory symptoms, minimising joint damage and improving function. It is less well appreciated that RA is associated with increased and premature cardiovascular (CV) mortality. This has not improved much over the last 3 decades, despite significant treatment advances. Almost half of all deaths in RA (and about 35-40% of the excess deaths) are due to cardiovascular disease (CVD) [2, 3].

The excess CV deaths in RA may be due to either an increased prevalence or increased case fatality of CVD in RA compared to the general population, and there is now evidence for both of these. Overall CVD (including individual risk factors such as hypertension and dyslipidaemia) is the commonest co­morbidity in patients with RA [4, 5]. Although rheumatoid heart disease is common on echocardiography or autopsy it rarely causes haemodynamic upset, so is an unlikely cause of death [6]. Instead, the evidence suggests that the main cause of increased CV death in RA is ischaemic heart disease (IHD). Mortality studies show that most CV deaths in RA are due to ischaemic pathologies such as myocardial infarction (MI) or congestive heart failure (CHF); probably due to accelerated atherosclerotic coronary heart disease (CHD) [reviewed in 2, 3]. Indeed, functional and phenotypic surrogate markers for atherosclerotic CHD, such as endothelial dysfunction [7], increased carotid intima-media thickness [8, 9] and changes in arterial elasticity [10] are commoner or more pronounced in RA patients than in controls, as are serological surrogates such as coagulation abnormalities [11-14]. The outcome of acute coronary syndromes is also worse in RA than matched controls [15, 16], probably due to increased instability of atherosclerotic plaques and enhanced stress responses [17] associated with high-grade systemic inflammation characteristic of RA.

It is now accepted that atherosclerosis, like RA, is a chronic inflammatory condition [18]. Immunohistochemical studies suggest significant similarities between the mechanisms responsible for chronic synovitis and damage in the rheumatoid joint and the generation and rupture of the atherosclerotic plaque in the vasculature, including the cellular infiltrates adhesion molecule expression, cytokine millieux, free radical and degradative enzyme release [reviewed in 19-21]. This is further supported by epidemiological work in the general population showing that several serological markers of systemic inflammation may associate with cardiovascular outcomes. The best studied is CRP, the level of which, even within the normal range, is a good predictor of future MI or ischaemic stroke in the general population, whether there is pre-existing CVD or not [22, 23]. Baseline CRP is also a predictor of CVD death in patients with early inflammatory arthritis, even within 5 years from the onset of arthritis [24]. RA disease activity as assessed by the erythrocyte sedimentation rate, raised fibrinogen levels [25], joint swelling [26] or a composite score [27] has been shown to associate with CV events, CV death and overall mortality respectively.

- - 1. The effect of current treatments on CVD risk and outcome in RA

Thrombotic variables may be positively or negatively affected by commonly used medications, particularly non-selective non-steroidal anti-inflammatory drugs (nsNSAIDs), cyclo-oxygenase 2 inhibitors (Coxibs) and antimalarials. The effects of other treatments for RA are less clearly defined: disease-modifying anti­rheumatic drugs (DMARDs) do not appear to increase overall mortality; in contrast, effective control of inflammatory activity appears to confer survival benefits [28-31]. However, it remains to be proven whether such benefits are due to improved CV outcomes, and although suppression of inflammation in RA makes good theoretical sense, we still do not know how to achieve it - and in which patients - without compromising the vasculature. For example, compared with other DMARDs, the use of methotrexate (MTX) in RA has been reported in one study to associate with reduced overall and CVD mortality in unselected patients [28], probably due to superior control of inflammation. In another study however, use of MTX in RA patients with pre-existing CVD led to a significant increase in mortality compared with other DMARDs [32]; this may be due to its anti-folate, thus potentially hyperhomocystinaemic effects [33]. Also, use of anti-TNF or potent anti-inflammatory/immunosuppressive therapy, although effective at reducing systemic inflammation, may [34] or may not [35, 36] associate with improvements in vascular function and surrogates of atherosclerosis. This emphasises the need for prospective, randomised, controlled trials to confirm any theoretical predictions.

- - 1. Classical cardiovascular risk factors in RA

Inflammation in RA may affect classical CVD risk factors, including lipid metabolism. Dyslipidaemia has been well documented in RA and appears to associate with the acute phase response [37]. Most studies suggest that during active RA, total and LDL cholesterol may be reduced, but HDL is consistently found to be even further reduced leading to an unfavourable lipid profile. Control of disease activity with several drugs or use of ciclosporin may lead to elevation of all lipid levels with the lipid profile remaining unfavourable.

Generally, RA patients have a higher prevalence of vascular risk factors [38] and signs of asymptomatic arterial disease [39] than matched controls, which may be present even before the onset of inflammatory polyarthritis [40]. About a third of unselected hospital Ra patients have documented CVD and/or CVD risk factors and are on treatment for these. Of the remaining two thirds, 90% have at least one modifiable risk factor. 70% and 60% have above the recommended levels of total cholesterol and systolic blood pressure respectively, and 40% have a >15% 10-year risk of a CHD event [13, 41]. Smoking, obesity and sedentary lifestyle may be important but are also more difficult to modify in RA than in the general population, due to the physical and psychosocial consequences of the disease [42]. As occurs in other high-risk groups (e.g. diabetes), pharmacological interventions targeted at hypertension and/or dyslipidaemia may be the most practical approach to this problem. Hypertension in RA may associate with the use of nsNSAIDs and Coxibs. Theoretically, ACE inhibitors (ACE-I) may have particularly beneficial effects in RA, since they have been shown to confer significant benefits in other high-risk populations [43], may have anti-oxidant properties and improve endothelial dysfunction [44]. However, other co-morbidities, polypharmacy, frequent treatment changes and the age groups characteristic of RA make both the routine use of such drugs problematic: for example, the combination of NSAIDs and ACE-I is commonly nephrotoxic, particularly in the elderly [45].

- - 1. Statins

Statins have a combination of properties that makes them particularly attractive for CV outcome studies in RA: they can be delivered in single daily dosing (an important factor in patients receiving multiple medications); they do not require much monitoring over and above that usually necessary for RA patients on DMARDs, and they do not interact adversely with most of the treatments commonly used in rA.

The efficacy of statins in the primary and secondary prevention of CHD events in the 'at risk population' has been demonstrated in several trials, with a reduction of major coronary events of 35% or above [45­51]. This is related almost exclusively to their lipid-lowering effects. There is however evidence from human in vivo studies, animal models and in vitro work, which suggest that statins have several other, so called "pleiotropic" effects. These include: reduced oxidative stress, anti-inflammatory and immunomodulatory actions, improved endothelial function, beneficial effects on vascular smooth muscle cells, antithrombotic effects and even antihypertensive properties. Several of these pleiotropic effects of statins are relevant to the atherosclerotic and chronic inflammatory pathologies of RA [52-57]. This opens up the possibility that statins may be beneficial to the rheumatoid component of RA [58, 59]. Indeed, the TARA trial [60], a 'proof of principle' trial demonstrated that atorvastatin 40mg daily, as an adjunct to DMARD therapy, provided additional benefit for inflammatory control of RA in at least a subgroup of patients.

With the exception of ciclosporin (which is metabolised through cytochrome P450 and can thus increase the risk of myopathy when used with some statins) there are no known significant interactions between anti-rheumatic drugs and statins. Statins are useful for secondary and primary prevention, in patients with overt CHD or those with a 10-year CVD (CHD+stroke) risk of >20%. Recent work shows that this is the case also for low risk patients, e.g. patients with diabetes with a <15% CHD risk [61]. Whereas many statin-effects may be class effects, there is evidence to suggest individual differences. Atorvastatin appears suitable for this pragmatic study. Several studies have shown a good safety profile over a wide dose range with no need to adjust dose for renal impairment. Improvements in cardiovascular end-points have been shown in many different settings and clinical populations such as ACS, stable CHD, diabetes and hypertension, including low CV risk diabetics. Early benefits seen in some atorvastatin studies have not been seen with other statins, particularly simvastatin, whereas newer statins (e.g. rosuvastatin) currently lack a large body of endpoint data. Atorvastatin may also have better effects on plaque progression and inflammatory marker reduction than either pravastatin or simvastatin [62-68]. Atorvastatin is also the only statin that has been used in an RA population with some "proof of concept" that, at a dose of 40mg daily, it can reduce systemic inflammation and improve lipid profile in RA [60], and has been shown to reduce arterial stiffness in patients with RA [69].

2.2 Rationale

2**.**2.1 Need for the trial

Cardiovascular morbidity and mortality in RA are common, severe and have not received adequate attention until very recently. No trials to date have addressed whether any intervention(s) can reduce the rate of CV events in patients with RA. Articles in leading medical journals, such as the Lancet [70] clearly identify the need for sufficiently powered and specifically designed trials addressing this problem.

The cause of the CV morbidity and mortality in RA is probably multifactorial, and may be best treated by a drug with pleiotropic effects in addition to standard lipid lowering. The proposed intervention is based on sound basic science and extensive clinical trial data in other populations. However, the efficacy and safety of such an intervention remains to be proven in the RA population which, due to its major morbidity, polypharmacy and concomitant muscle pathology has been systematically excluded from all major statin trials.

1. Aim of the trial

To establish whether atorvastatin will protect patients with RA aged >50 years or with >10 years' duration of RA from fatal and non-fatal atherosclerotic events.

A nested sub-study (TRACE RA DAS) has also been designed to determine whether atorvastatin, used in conjunction with standard DMARD therapy, will provide added arthritis control in patients with moderately or severely active RA.

A further sub-study, (TRACE RA BioBank) has also been incorporated into the main study. The aim of this sub-study is as follows:

1. To develop a DNA repository for the 3808 patients with rheumatoid arthritis (RA) enrolled in the main TRACE RA study.
2. To develop a plasma and serum repository for the above patients.
3. To devise and complete peer reviewed projects on the above samples using the phenotypic and outcome data collected in the TRACE RA trial.
4. To take advantage of established population-based record-linkage capability, to allow longitudinal tracking of subjects and thereby enabling investigation of the relationship between susceptibility genotypes and pertinent clinical features including characteristics at diagnosis, response to treatment, development of RA complications, vascular disease and survival/
5. How the results will be used

If effective, the implementation of this intervention in the routine rheumatology clinic would be easy and, apart from the drug costs, would have no resource implications.

The trial will increase awareness of CV morbidity and mortality in RA and, in the process, identify patients who need to be properly risk-assessed and treated.

If atorvastatin also shows evidence of being a disease-modifying drug this will add to the choice of such drugs, which is at present limited. Current evidence suggests that the goal in RA treatment should be to minimise the inflammatory response - ideally aiming for remission. This is likely to slow or halt radiographic progression and to improve cardiovascular co-morbidity and mortality. All additions to the family of drugs which reduce inflammation are welcome.

1. Trial Design

Trial type

Multi-centre, randomised, double blind, placebo-controlled trial Primary hypotheses

TRACE RA - All patients: Atorvastatin is more effective than placebo in the primary prevention of cardiovascular events in RA patients.

TRACE RA DAS patients only: Atorvastatin is more effective than placebo as adjunctive therapy for the reduction of disease activity in RA patients

- 1. Primary endpoint

TRACE RA (all patients): Co-primary Endpoints

- Cardiovascular death, non-fatal myocardial infarction or stroke
- As above plus coronary revascularization

TRACE RA-DAS (sub-study only):

- EULAR moderate or good response based on DAS 28 at 6 months
  1. Secondary endpoints TRACE RA

All-cause mortality

Examination of the coronary and cardiovascular components separately Carotid revascularization Hospitalised angina

Fatal and non-fatal peripheral atherosclerotic events Changes in fasting lipids and changes in CRP Functional outcome assessed by HAQ and EQ5D Statin safety-related outcomes Hospitalisation for any cause Number of nights spent in hospital

TRACE RA-DAS

DMARD (Disease Modifying and Anti-Rheumatic Drugs) changes DAS 28 at month 12 and 24.

Radiological outcome (assessed by changes in the Larsen score in x-rays of hands and wrists at baseline and year two)

Functional outcome assessed by HAQ and EQ5D at month 6, 12 and 24.

- 1. Trial intervention

The active treatment will comprise of Atorvastatin 40mg taken once daily. The placebo will be a matching dummy, taken once daily. Pfizer UK Ltd will provide both.

In addition all patients will be counselled about any other modifiable CV risk factors identified at the screening visit. GPs will be informed of any patients found to be hypertensive, to have diabetes or to have a recognised indication for statin use according to current clinical practice.

1. Study Organisation

TRACE RA will randomise approximately 3800 patients, from up to 100 centres in the United Kingdom (UK). The aim is to complete accrual within 18 months, by maximising the number of centres and the speed with which the centres are activated.

The planned study initiation is January 2007, the completion of enrolment is mid 2008 and completion of follow up is planned for mid/end 2014. The principal investigators, trial manager, data manager, trial co­ordinators and senior investigators will form a trial management group to execute the trial according to ICH GCP. The three principal investigators plus the trial manager will form an executive committee.

There will be trials units based in Dudley, Dundee and Manchester to oversee the management of this trial according to ICH GCP. The trials units will support their affiliated recruiting centres and monitor data management for the trial. Each recruiting centre will agree affiliation to one of the trials units prior to participating in the study. However, it may be necessary to make some changes during the trial to balance the workload between trial units.

Data generated will be collected by the trials units, which are responsible for checking incoming case report forms (CRFs) for compliance with the protocol, inconsistent and missing data, and for resolving data queries. Data from all recruiting centres will be pooled at the ARC Epidemiology Unit, University of Manchester, who maintain overall responsibility for all trial data, and for the Standard Operating Procedures (SOPs) that describe how the trial is to be conducted within participating trials centres and units. All data will be handled, computerised and stored in accordance with the Data Protection Act 1998. Quality control of data will be maintained by the University of Manchester through regular meetings to discuss data management with the other trials units.

Data and statistical analysis will be overseen by Dr Peter Nightingale (trial statistician) who is based at the Wellcome Trust Clinical Research Facility in Birmingham.

Patients entered into the TRACE RA trial should not be asked to participate in another pharmaceutical clinical trial for the duration of their participation in the TRACE RA trial. They may, however, participate in longitudinal observational studies such as the BSR Biologics Register and comparison cohort.

1. **Eligibility**

Inclusion criteria:

- Patients who satisfy 1987 ACR classification criteria for RA applied cumulatively [71]
- Age >50 years old OR >10 years of RA disease duration
- Written informed consent

Exclusion criteria:

- Pregnancy, breast-feeding or women of child-bearing potential not using adequate contraception
- Known primary muscle disease
- Known atherosclerotic disease i.e. previous episodes of confirmed Acute Coronary Syndrome (ACS), unstable angina; myocardial infarction with or without ST elevation; or stable CHD/CVD deemed to require statin therapy on clinical grounds, including:
- Previous amputation due to severe peripheral vascular disease or current peripheral arterial disease
- Previous central or peripheral revascularisation procedure (including angioplasty or stent, artery bypass graft surgery)
- Accelerated hypertension, severe heart failure (class III or IV), significant dysrhythmia or angina requiring hospitalisation in the 6 months preceding potential study entry
- Uncontrolled hypertension (treated or untreated) defined as systolic blood pressure > 200 mmHg and/or diastolic blood pressure > 110 mmHg (identified as the disappearance of all sound (Korotkoff Phase V) after sitting quietly for at least 3 min)
- Previous cerebrovascular accident
- Other accepted indication for statin therapy according to the investigators' current clinical practice
- Known familial hyperlipidaemia requiring drug therapy
- Known diabetes
- Known hypersensitivity or intolerance to statins
- Active liver disease or hepatic dysfunction with AST or ALT >2x upper limit of normal (ULN)
- Severe renal dysfunction (Creatinine >200 micromol/l)
- Creatinine phosphokinase (CK) >3xULN
- Uncontrolled hypothyroidism
- Participation in another clinical trial (other than observational or lifestyle studies and registries) concurrently or within 30 days prior to screening for entry into this study
- Other serious illness or significant abnormalities that may compromise the patient's safety or successful participation in the study
- Any illness which in the doctor's opinion means that the patient is unable to give informed consent
- Known alcohol abuse
- Taking any of the following medications:
  - - Other HMG-CoA reductase inhibitors (unless warranted as add-in therapy for post-endpoint management of study patients. In such a case during the course of the trial, it will be suggested to the managing physician to start initially
    - Atorvastatin at a dose of 10mg daily (or equivalent dose of another statin of their choice).
    - Drugs known to be associated with rhabdomyolysis in combination with HMG-CoA reductase inhibitors (e.g. ciclosporin, erythromycin, azo anti-fungals, protease inhibitors and all macrolides)
    - Drugs (other than beta-blockers, diuretics, ACE inhibitors, other anti-hypertensive agents, oral hypoglycaemic agents and thyroxine replacement therapy) known to affect lipid levels, that interact with the study medications, or that may affect clinical laboratory parameters (such as isotretinoin).
    - For the TRACE RA-DAS sub-study, intermittent systemic (intravenous or intramuscular) steroids are not allowed later than 2 month prior to measurement of clinical and laboratory outcome parameters (both at baseline or follow-up assessments for the purposes of the trial). Intra- articular steroid injections are allowed, but any joint injected within the previous 2 months will be counted as both tender and swollen in the 28 joint counts.
    - Lipid-regulating drugs: probucol, fibrates and derivatives, bile acid sequestering resins. Patients currently taking a lipid-altering drug may be considered for screening after a 4-week wash-out period except in the case of probucol where medication must have been discontinued for at least 6 months.
- Drinking more than one 240ml glass of grapefruit juice per day

1. **Trial Procedures**
   1. Patient selection and informed consent

Once a patient has been selected based on the eligibility criteria, sufficient time will be provided for the patient to decide on trial entry, but the time which elapses between randomisation and start of treatment should be minimised (no longer than four weeks).

Potentially eligible RA patients will be identified from their medical records based on the presence of inclusion/exclusion criteria using a specific proforma. Information on the trial will be given to potentially eligible patients and they will be asked to give their written informed consent. Patients will be checked to ensure they are not already on a statin and do not have an existing indication for a statin according to standard clinical practice.

The eligibility checklist, consent form, randomisation registration form and allocated trial number will be faxed back to the relevant trials unit prior to the patient being dispensed the drug.

Trial ID numbers will be provided on pre-printed CRFs - a trial number from the CRF used for each patient will be the patient's trial number for the duration of the trial. 6.2 Trial investigations: (Please see Appendix 11 for trial evaluation schema) 6.2.1 Baseline/randomisation visit

Patients will be given an opportunity to ask any questions they may have, and then asked to provide their written informed consent to enter the study.

- Following this the patient will have the following examinations:

® Medical History

® Family history of cardiovascular disease (CVD)

® Smoking status ® Record of concomitant medication ® 28 tender and swollen joint count ® Height, Weight, Waist circumference and blood pressure ® ECG

- Participants will be asked to complete the following questionnaires:

® Patient global assessment (VAS)

® HAQ [72] and EQ5D [73] questionnaires ® Lifestyle factor questionnaire

- And be asked to give a blood sample:

® Routine Haematology, Biochemistry, ESR/CRP and RhF tests

All patients will also be asked to consent to the optional TRACE RA BioBank sub study (Appendix 2).

- For TRACE RA-BioBank Sub study participants (Optional)

® 10mls plasma (citrate) blood sample ® 10mls serum (clotted) blood sample

® 2 x 10mls EDTA blood sample (immediately stored at -80°C)

Patients who have a DAS28 score of >4.4 will be asked to consent in addition to the TRACE RA DAS sub study (Appendix 1). The DAS28 (Disease Activity Score) [74] will be calculated using the most recent ESR/CRP. Outcome measures will be recorded in the CRF and patient hospital notes (See appendix 6). This sub study will only be run at selected centres.

- For TRACE RA DAS Sub study participants (Optional)

® X-ray of hands and wrist ® Nurse global assessment (VAS)

® 2 x 2.5ml blood sample (for RNA testing)

All patients will be counselled by the study nurse or doctor about any modifiable cardiovascular risk factors identified, e.g. smoking or obesity (BMI>30kg/m^2^) and given a copy of a leaflet prepared for the TRACE RA trial.

Eligible patients not consenting to randomisation will be asked for their written informed consent to have their records electronically tagged. Mortality in this group will be compared with that of the study group as a measure of generalisability, and their screening information will be used to assess predictors of mortality.

Patients ineligible due to having an existing indication for a statin will also be asked for their written informed consent to have their records electronically tagged.

GPs will be informed of all patients found to be hypertensive, to have diabetes or an existing indication for a statin at the screening visit using a standard letter.

Patients will then be randomised to either the atorvastatin arm (40mg of atorvastatin oral tablet taken once daily) or placebo arm (Placebo atorvastatin oral tablet taken once daily) of the trial. The patient will also be registered with the trials units in order to obtain confirmation of the trial number for anonymised analysis of their medical information and subsequent dispatch of the trial drug.

1. Allocation of patients to trial treatment arm

The study will be double blind with matching placebo. Neither patients, investigators nor trial units will be aware of the treatment allocation. The medication will be provided by Pfizer UK Ltd., bottled by an independent pharmaceutical company (Cardinal Health UK Limited) to GMP standards and dispensed by the local study pharmacist.

The randomisation process will be incorporated into the drug labelling. There will be two sets of unique drug labelling numbers; one for the main study (Set A) and one for the TRACE RA -DAS sub-study (Set B). The initial bottles of each set will be delivered in blocks of four. Each block of four will include two bottles containing placebo and two bottles containing active drug.

Each patient will be allocated a filled and labelled bottle coded with a unique drug number when entering the trial. All future supplies for this patient will be coded with the same unique drug number. Patients will continue on the same treatment (i.e. active or placebo) throughout the duration of the study.

Scratch cards will be supplied to the local pharmacy so that the code can be broken if necessary. Cardinal Health will provide the independent trial statistician with information about treatment allocation by unique drug number to enable the interim analyses to be conducted.

A TRACE RA SOP will be in the study site file for further information on providing treatment to patients.

1. Follow-up visits for TRACE RA participants

Patients will attend visits during the following months:

- Month 3 from first administration of trial drug

Check drug compliance Side effects/Drug toxicities

- Month 12, 24, 36, 48, 60 and 72 from first administration of trial drug -

Cardiovascular outcomes (e.g. Occurrence of heart attacks or strokes)

Length of any in-patient stay Haematology and Biochemistry blood tests Record of current medication HAQ and EQ5D Side effects/Drug toxicities

- End of Trial visit (At Month 60 or 72)

Height, weight, waist circumference and blood pressure ECG

Compliance with study medication will be checked at each visit.

Follow-up visits for TRACE RA DAS participants · Month 6 from first administration

of trial drug (TRACE RA DAS sub-study only)

Patient Global (VAS)

Nurse Global Assessment (VAS)

28 tender and swollen joint count ESR/CRP

- Month 12 from first drug administration of trial drug (TRACE RA DAS sub-study only)

Patient Global (VAS)

Nurse Global Assessment (VAS)

28 tender and swollen joint count ESR/CRP

- Month 24 from first administration of trial drug (TRACE RA DAS sub-study only)

X-ray of hands and wrists Patient Global (VAS)

Nurse Global Assessment (VAS)

28 tender and swollen joint count ESR/CRP

Patients in the TRACE RA DAS sub-study will have an additional visit at 6 months at which the components of the DAS28 score will be checked. Consultants will be asked not to change the DMARD therapy of patients enrolled in the disease activity sub-study for the first 6 months of the clinical trial.

Additional visits to the rheumatology clinic and changes in medication will be decided based on clinical need. Patients will be given the telephone number of their consultant or Rheumatology department/helpline in case they have any concerns whilst enrolled on the trial.

Patients will continue the intervention for the entire period of their participation in the trial, unless the trial steering committee decides to discontinue the study for any reason. Any patients who discontinue the intervention will remain under follow up.

1. Procedure for unblinding

Each hospital pharmacy will have a nominated person that will be able to break the randomisation code for an individual patient if required in a medical or other emergency. The code (scratch card) for each individual patient will be kept securely in the pharmacy. The local and main co-ordinating centre will be informed each time the code is broken and will be given the reason for the unblinding.

1. End of trial

The trial will end five years after the recruitment of the last patient, or after the last patient has attended their five year review, whichever is the later. All patients will attend for annual review until the trial has been completed and will stop their medication on the day of their final assessment. All events which occur prior to this final assessment will be included in the analysis whether or not the patient has continued to take the trial medication. Because of the 'intention-to-treat' (ITT) analysis, it is essential to follow all patients up to this 'final assessment' date.

1. **Study Withdrawal**

Patients will be withdrawn from the study if they experience a cardiovascular event or develop any condition for which statin therapy is clearly indicated. They will stop the trial medication and be managed appropriately as per their physician's current clinical practice, while they will continue to be flagged for mortality. They will be included in the ITT analysis.

After study entry, patients will not be routinely screened for the 10 year CVD risk as part of the trial. If screening is done opportunistically and a statin is felt to be indicated for primary prevention then the managing physician will be advised to add in atorvastatin at a dose of either 10mg or 40 mg as clinically indicated. The patient will continue in the trial taking both the study medication and the prescribed atorvastatin. A record of concomitant statin prescription will be made at each annual visit.

If the trial is stopped early for safety reasons, all patients will stop the study medication. If it is stopped early due to efficacy of the statin arm, all patients will be offered the opportunity to continue/commence statin therapy. If the trial goes its full length, all patients will stop the trial medication and be managed according to their physician's preference pending full analysis and publication of the trial findings.

Patients are free to withdraw from the trial at any point. This will have no implications on their future care.

1. **Drug Supplies and Labelling**

Atorvastatin and the placebo atorvastatin will be supplied free of charge by Pfizer UK Ltd. Further details on costing can be found in the "Research Governance" section of the protocol.

- 1. Packaging and labelling of study medication

The drug will be packaged in bottles and labelled by Cardinal Health according to GMP standards.

Bottles will be labelled with a minimum of the following information:

- Unique drug number (number allocated to drug bottle)
- Packaging lot number
- Expiry date
- Number of tablets in bottle
- Dosage instructions
- Storage conditions

Bottling and labelling will be done in several runs spaced throughout the trial. All bottles will contain extra tablets to allow for delays in patient visits.

- 1. Supply of study medication to centres

Cardinal Health will supply the packaged drugs to the pharmacies of individual hospitals at regular intervals throughout the trial.

Patients will be asked to return the remainder of their trial medication to pharmacy at each visit in order that a compliance check can be made and drug accountability can be undertaken. The pharmacy will store the returned trial medication and the trial monitors will perform the drug reconciliation/compliance at each monitoring visit.

Patients will be provided with a 3 month supply of the study medication at the baseline and Month 3 visits. After this, they will be provided with a 6 month supply of the study medication. The trial pharmacist will be provided with a drug reconciliation label sheet and each time the trial drug is dispensed, the pharmacy will be asked to affix the supplementary drug label to the sheet so that a 'dispensation record' can be maintained for the purposes of the trial.

1. **Pharmacovigilance**
   1. Cardiovascular Outcomes and reporting of cardiovascular outcomes

Any cardiovascular events that occur to patient should be reported on the cardiovascular outcome form that will be provided in the site file as these events will be classified as part of the endpoints of the trial.

All information regarding a cardiovascular event should be faxed to the University of Manchester on 0161 275 5043 within 24 hours of knowledge of the event. Follow-up information may be required after completion of protocol treatment if necessary. The local principal investigator or research nurse will be contacted by the trials unit if further information is required.

- 1. Adverse events

Adverse event (AE): any untoward medical occurrence in a clinical trial subject and which does not necessarily have a causal relationship with treatment

An adverse event includes:

1. an exacerbation of a pre-existing illness
2. an increase in frequency or intensity of a pre-existing episodic event/condition
3. a condition (even though it may have been present prior to the start of the trial) detected after trial treatment administration
4. continuous persistent disease or symptoms present at baseline that worsens following administration of the trial treatment

An adverse event does not include:

1. medical or surgical procedures - the condition which leads to the procedure is the adverse event
2. pre-existing disease or conditions present before treatment that do not worsen
3. situations where an untoward medical occurrence has occurred e.g. cosmetic elective surgery
4. overdose of medication without signs or symptoms
5. the disease being treated or associated symptoms/signs unless more severe than expected for the patient's condition
   1. Adverse Reactions

Adverse reaction to an investigational medicinal product (AR): any untoward and unintended responses to an investigational medicinal product (IMP) related to any dose administered

All adverse events judged by the reporting investigator as having a reasonable causal relationship to an IMP qualify as adverse reactions. The expression reasonable causal relationship means to convey in general that there is evidence or argument to suggest a causal relationship.

An initial adverse event form will be included in the CRF for completion at each study visit. If the event is deemed as serious by the local principal investigator, a serious adverse event report form is provided in the site file to be completed by the research team and faxed to the trials unit within 24 hours of knowledge of the event.

- 1. Unexpected Adverse Reactions

Unexpected adverse reaction: an adverse reaction, the nature, or severity of which is not consistent with the applicable product information (e.g. Investigator’s Brochure for an unapproved investigational product or Summary of Product Characteristics (SPC)/Package Insert for an

authorised product)

The investigator should keep detailed records of all AEs. Each AE should be evaluated with respect to seriousness, severity, causality and expectedness.

The term severity is used to describe the intensity of a specified event. This is not the same as serious, which is based on patient/event outcome or action criteria.

- 1. Causality

Causality refers to the relationship between the trial treatment and the adverse event.

Each AE should be assessed as follows:

Seriousness: serious, not serious.

Severity: mild, moderate, severe.

Causality: probably related, possibly related, unlikely to be related, unrelated to treatment. Expectedness: expected, unexpected.

- 1. Serious Adverse Events/Reactions (SAEs/SARs)

Serious adverse event (SAE) or serious adverse reaction (SAR): any untoward medical occurrence or effect that at any dose

- results in death,
- is life-threatening (i.e. with an immediate, not hypothetical, risk of death at the time of the event),
- requires hospitalisation or prolongs existing hospitalisation (excluding hospitalisation for elective treatment of a pre-existing condition),
- results in persistent or significant disability or incapacity,
- is a congenital anomaly or birth defect (i.e. the outcome of pregnancy involving the patient)
- any other important medical condition which, though not included in the above, mayjeopardise the patient and may require medical or surgical intervention to prevent one of the outcomes listed (e.g. allergic bronchospasm requiring intensive emergency treatment, seizures or blood dyscrasias which do not result in hospitalisation, or development of drug dependency).

Medical judgement should be exercised in deciding whether an adverse event/reaction is serious in other situations.

Death will not be considered as a SAE/SAR in the TRACE RA trial as it is one of the secondary endpoints of the trial. However, all information of the cause of death should be provided in the 'Death Form' in the CRF if it is available.

- 1. Suspected Unexpected Serious Adverse Reactions (SUSARs)

A SUSAR is both a serious and unexpected adverse reaction, the nature, or severity of which is not consistent with the applicable product information (e.g. investigator brochure for an unapproved investigational product, or summary of product characteristics for an authorised product). SUSARs require expedited reporting by the trial sponsor (i.e. Chief Investigator and University of Manchester) to MHRA, therefore every effort should be made to notify the regional trials office within the timeframe shown below.

As a general rule, the treatment code for the specific patient should be broken before reporting a SUSAR. Events associated with placebo will usually not satisfy the criteria for a SUSAR.


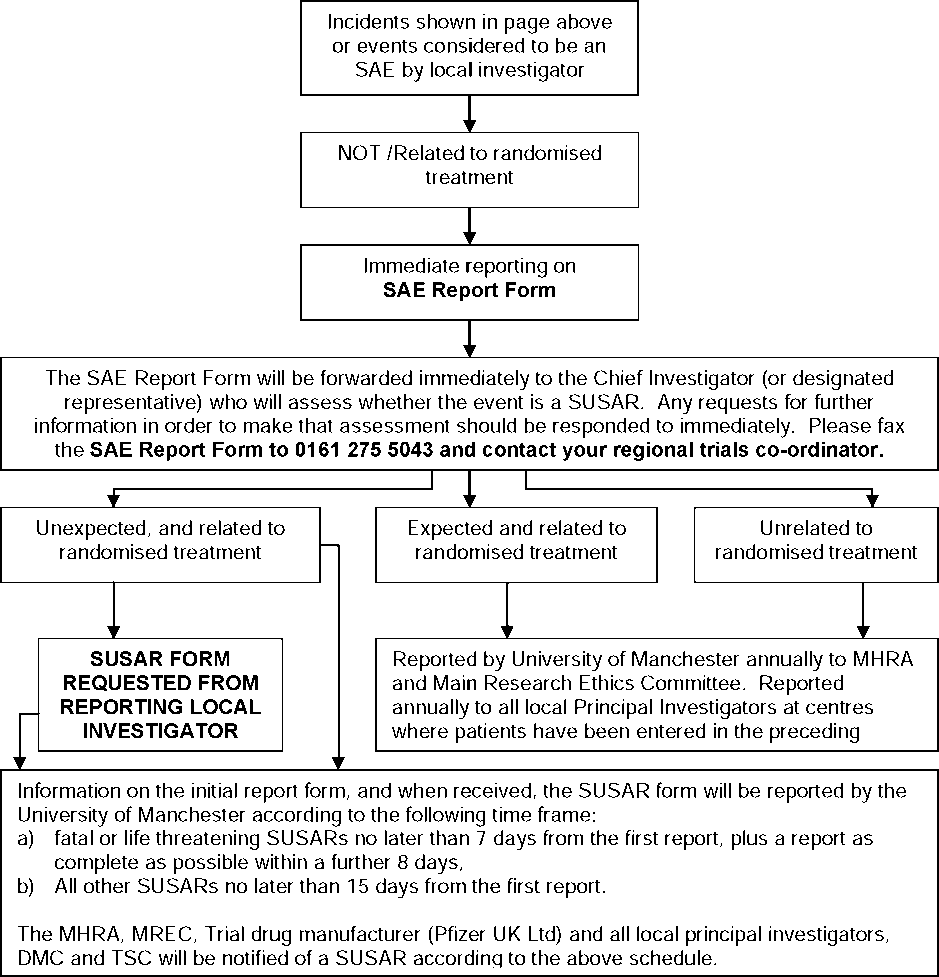


1. Follow-up of SAEs and SUSARs

9.8 Recording and reporting of all SAE/SARs

Flow diagram of SAE reporting and action taken following the report:

The patient must be followed-up until clinical recovery is complete and laboratory results have returned to normal, or until disease has stabilised. Information on final diagnosis and outcome of SAEs which may not be available at the time the SAE is initially reported should be forwarded on the SAE initial report form as soon as this information is available. Follow-up may continue after completion of protocol treatment if necessary.

All information regarding an SAE should be faxed to the University of Manchester on 0161 275 5043, and information required by the drug manufacturer will be passed on by that trials unit. Centres are free to volunteer information to the drug manufacturer if they wish, but are under no obligation to do so.

1. **Statistical Considerations**
   1. Sample size

A total sample of 3808 patients is required for the logistic regression analysis of the 5 year outcomes to have 80% power to detect a significant difference (at the 5% level) between the statin treated and placebo treated arms of the study. This assumes a 1.8% annual event rate in the placebo treated arm, a 32% reduction in this rate for the statin treated arm and a 20% total drop-out rate (Appendix 8). With these same assumptions the Cox regression analysis of 5 years of follow-up would require a sample of 3548 patients or a total of 208 events for 80% power. The trial will continue (if necessary) until at least this number of events has been accrued and all the available follow-up data will be used in the Cox regression analysis.

- 1. Planned recruitment rate

Recruitment of the required sample is expected to be complete within 18 to 24 months. Recruitment will start simultaneously in all centres. The collaborating centres in this trial each follow-up between 500 and 3500 RA patients/annum, so the planned recruitment rate, although ambitious, is feasible.

With the anticipated support of 60 - 100 centres/sites within the UK, it is predicted than an average recruitment rate of around 2 - 4 patients per centre per month would achieve this recruitment target.

To facilitate recruitment, the trial manager will be in post at least 6 months and the trial co-ordinators will be in post before the start of the recruitment period. Research nurses employed at the regional co­ordinating centres will also be in post before recruitment begins.

The trial will be publicised through the arc's "Arthritis Today" magazine and through the National Rheumatoid Arthritis Society (NRAS) as well as local resources. A British Society for Rheumatology (BSR) special interest group has been set up to act as a forum for the study investigators.

- 1. Compliance

A non-compliance rate of not more than 4% is anticipated. Compliance will be assessed by a count of pills in bottles returned to the local pharmacy. This will be carried out by the trial co-ordinators during their monitoring visits.

- 1. Loss to follow-up

The loss to follow-up rate will be low as the great majority of patients will be under long-term rheumatology follow-up. We have allowed a withdrawal or discontinuation rate of 20% over the study period. This is about 70% of that observed in WOSCOPS (primary prevention) [47], but almost double that observed in 4S (secondary prevention) [48].

- 1. Planned analyses

All patients randomised and who have taken at least one dose of the study drug will be included in the analysis, irrespective of whether the study drug is continued (ITT analysis). Logistic regression models will be developed with the occurrence of a first cardiovascular event or adverse event as the dependent variable and treatment allocation as the independent variable.

The models will be adjusted for factors used in the stratification and for any baseline imbalances. However, the trial is of sufficient size to expect that most potential confounders (e.g. smoking, aspirin, NSAID/Coxib, corticosteroid, anti-TNF usage etc) are likely to be balanced between the groups. Treatment differences will be expressed as odds ratios with 95% confidence intervals. Two-sided p values of <0.05 will be considered significant.

Survival analysis, comparing the time from randomisation to the occurrence of the first primary cardiovascular endpoint, will also be performed using a Cox regression analysis that will again adjust for baseline measures and recruiting centre effects. Kaplan-Meier Product Limit (PL) estimates of the survival curves with 95% confidence intervals will be calculated.

Analysis of rheumatology outcomes

All patients randomised in the disease activity substudy trial will be included in the analysis, irrespective of whether the study drug is continued (intention-to-treat analysis). The dependent variable will be the dichotomous variable of EULAR response. Any further baseline variables can be incorporated into the model if necessary to adjust for baseline imbalances. Treatment differences will be expressed as odds ratios with 95% confidence intervals. Two-sided p values of <0.05 will be considered significant.

- 1. Procedure for accounting for missing data

Tests will be conducted to explore whether missing data are missing at random and appropriate tests then deployed to impute the missing data.

- 1. Planned sub-group analyses

Sub-group analyses will be carried out with respect to: sex; age (<65 vs. >65 years); RA disease duration; rheumatoid factor status and anti-TNF therapy (if numbers are sufficient). It is recognised that these sub­analyses will have less statistical power than those based on the whole study sample and this will be reported appropriately.

- 1. Interim analyses and its frequency

Interim analyses will be performed when the number of events reaches 25%, 50% and 75% of the total expected events. No formal stopping rule will be set but the DMC will advise the Chairman of the Trial Steering Committee that the trial should be stopped if, in DMC's view, the randomised comparison in the trial has provided both (a) proof beyond reasonable doubt that for all, or for some, types of patients the trial treatment is clearly indicated or clearly contraindicated in terms of a net difference in major morbidity and mortality, and/or (b) evidence that might reasonably be expected to influence the patient management of clinicians aware of the results of any other studies. The use of a p-value of <0.0005 would be expected for this. Safety data will be continuously monitored by the independent DMC.

- 1. Economic analyses

The trial will not address any economic issues directly but, since EQ5D can be used for health utility assessment [75], the results can subsequently be used for economic modelling and cost-effectiveness based on calculation of QALYs gained.

The trial will also monitor the number of hospitals admissions and length of stay that occur via information collated from the CRFs.

1. **Assessment of Efficacy and Safety**
   1. Assessment of efficacy outcomes

English and Welsh patients' records will be electronically tagged for mortality with the Office for National Statistics (ONS), and Scottish patients at the Scottish Office's Information and Statistics Division (ISD) as well as the local hospital Trusts' Medical Information departments. Separate ethical approval will be obtained for this from the ISD ethics committee. Underlying cause of death will be ascertained from death certificates provided by ONS and ISD (which also supply details of times and causes of all hospital admissions in Scotland) supplemented by information from hospital records, including post-mortem examinations, if performed.

Hospital admissions will be ascertained at each centre by matching the patient details with national NHS Hospital Episode Statistics. Non-fatal events will be ascertained annually and at the end of the study by contact with each patient and/or household members, GP and hospital departments, with relevant documentation, using a standardised proforma. The proforma and relevant hospital records of patients with suspected non-fatal endpoint events will be provided to the "endpoints classification committee" who will determine and categorise the event for analysis. Participants will be withdrawn (censored) from the study once they have experienced a cardiovascular event or develop any condition for which statin therapy is indicated for secondary prevention.

- 1. Assessment of safety

Statin safety-related outcomes, as stated in Appendix 3, will be assessed continuously by the independent DMC.

All patients will be asked about any adverse events they have experienced at each follow up visit.

The local principal investigator at each site must assess all adverse events for seriousness and causality and report all serious adverse events (defined as events requiring or prolonging hospital admission, leading to death or congenital abnormality) immediately to the local and main co-ordinating centre. The immediate report should be followed by a detailed written report. Serious adverse events that are listed as primary endpoints do not require immediate reporting. The main co-ordinating centre will report all serious adverse events (which are not primary endpoints) to the DMC within 24 hours of knowledge of the event, whether or not it has been attributed to the trial intervention.

The main co-ordinating centre will report all suspected unexpected serious adverse reactions (SUSARs) to the MHRA, the main REC and the DMC. SUSARs that are fatal or life-threatening will be reported as soon as possible, but no later than 7 days after knowledge. All other SUSARs will be reported as soon as possible, but within a maximum of 15 days.

Serious adverse reactions will be classed as SUSARs if the nature or severity of the reaction is not consistent with those previously observed. Please see Appendix 6 for a list of previously observed reactions to atorvastatin.

Patients should continue with their DMARD monitoring as normal. If the patient has an isolated abnormality in liver function (LFT) without a rise in creatinine kinase levels (CK), then this should be attributed to the statin in the first three months. Thereafter any DMARD or non-steroidal is the more likely cause and rheumatologists will be recommended to consider stopping these drugs prior to stopping the trial medication. Following this, the trial medication may be stopped for up to 4 weeks while the cause of any LFT abnormality is investigated without having to withdraw the patient from the trial.

1. **Research Governance**
   1. Trial administration and logistics

Dudley Group of Hospitals NHS Trust and the University of Manchester are co-sponsors of the TRACE

RA trial. Sponsorship activities and delegated responsibilities are shared between Dudley Group of

Hospitals nHs Trust, the employer of the Chief Investigator (CI); and the University of Manchester, in

accordance with the UK Medicines for Human Use (Clinical Trials) Regulations 2004 and in line with the

Research Governance Framework for Health and Social Care, April 2005 2^nd^ Edition, and according to

ICH GCP. Both parties agree to allow inspection of sponsors' premises by the competent authorities.

Dudley Group of Hospitals NHS Trust (DGOH) & Chief Investigator responsibilities:

- Put and keep in place arrangements to adhere to ICH GCP
- Ensure that Investigational Medicinal Products (IMPs) are made available to subjects free of charge
- Take appropriate urgent safety measures
- Ensure that Pharmacovigilance is maintained throughout the duration of the trial - please note that the administration of Pharmacovigilance for the trial has been delegated to the University of Manchester by the Chief Investigator.
- Ensure that PIs conduct the study in accordance with ICH GCP, the DOH Research Governance Framework and laws and statutes that relate to the study and any local requirements as may be specified by their host institution.
- Responsibility for putting and keeping in place arrangements to conduct the study according to Good Clinical Practice, the DOH Research Governance Framework and the laws and statutes that relate to the study
- Responsibility to use all reasonable efforts to ensure that the data collected and reported are accurate, complete and identifiable at source; and that record keeping and data transfer procedures adhere to the Data Protection Act 1998.
- Responsibility for monitoring the study in accordance with the arrangements outlined in the submission to the Sponsor.
- Responsibility to supply documentation and reports as deemed necessary by the Sponsor to fulfil its obligations.
- Responsibility to cooperate with audits or inspections undertaken by the host institution, the Sponsor and regulatory authorities, including the MHRA, as required.
- Responsibility to assist investigations into any alleged research misconduct undertaken by or on behalf of the Sponsor.
- Responsibility to make the necessary provision for archiving essential documents.

ARC Epidemiology Unit, University of Manchester responsibilities:

Trial Administration Responsibilities:

- Request Clinical Trial Authorisation (CTA) and make any amendments that are required for the authorisation
- Undertake to allow inspection of co-sponsors premises
- Gain appropriate authorisations prior to starting the trial, including authorisation from both the NHS Trust research offices and the University of Manchester, ethical approval.
- Give notice of the following events to the appropriate regulatory bodies:

o amendments to CTA, make representations and amendments o amendments to the protocol o the termination of the trial

- Maintain a Master File containing essential trial documents and to make the file available for statutory inspections by bodies such as the MHRA

Financial Responsibilities:

- Administer funding and co-ordinate any required legal agreements and investigator statements or agreements.

Responsibilities that have been delegated by DGOH & CI:

- Keep records of all adverse events reported by investigators
- Ensure recording and prompt reporting of suspected unexpected serious adverse reactions (SUSARs) to the Chief Investigator
- Ensure investigators are informed of SUSARs
- Provide an annual list of suspected adverse reactions and a safety report to the relevant authorities and committees.

The following responsibilities are retained by the Chief Investigator, or in his absence, a named deputy(s):

- Prompt decision making as to which serious adverse events are SUSARs, and prompt reporting of that to the University of Manchester for onward reporting to the licensing authority.

The following responsibilities are delegated by the Chief Investigator to the local Principal Investigators at each trial centre:

- Obtain Management (R&D/ Research Governance) approval
- Responsibility for putting and keeping in place arrangements to conduct the study according to Good Clinical Practice, the DH Research Governance Framework and the laws and statutes that relate to the study
- Responsibility to liaise with Pharmacy to document the supply, handling and accountability of all trial drugs
- Responsibility to ensure that all members of the study team have sufficient knowledge, training and experience to undertake the roles assigned to them and to comply with requirements as specified by the host organisation
- Responsibility to maintain a Site File (containing the essential documents) and to make the site file available for inspection if requested by the CI (on behalf of the Sponsors)
- Responsibility to conduct the study in accordance with the agreed research protocol except where necessary to eliminate (an) immediate hazard(s) - These circumstances must be reported to the CI who will be responsible for reporting these events on behalf of the sponsor organisations, to the research ethics committee and the MHRA
- Responsibility to use all reasonable efforts to ensure that the data collected and reported are accurate, complete and identifiable at source; and that record keeping and data transfer procedures adhere to the Data Protection Act 1998
- Responsibility to supply documentation and reports as deemed necessary by the Sponsor
- Responsibility to cooperate with audits or inspections undertaken by the host institution, the Sponsors and regulatory authorities, including the MHRA as required.
- Responsibility to assist investigations into any alleged research misconduct undertaken by or on behalf of the Sponsors
- Responsibility to make the necessary local provision for archiving essential documents

The delegation of sponsorship responsibilities does not impact on or alter standard NHS indemnity cover. The agreement of delegated responsibilities is viewed as a partnership and as such it is necessary to share pertinent information between the University of Manchester and the Dudley Group of Hospitals NHS Trust/Chief Investigator, including proposed inspections by the MHRA and/or other regulatory bodies.

- 1. Compliance to Protocol

TRACE RA is being conducted in accordance with the professional and regulatory standards required for

non commercial research in the NHS under the UK Medicines for Human Use (Clinical Trials) Regulations

2004. Before activating the trial, participating centres are required to sign an agreement accepting delegated responsibilities for all trial activity which takes place within their centre.

- 1. Good Clinical Practice

This trial will be conducted in accordance with the protocol, the conditions and principles stipulated in the

MRC guidelines of Good Clinical Practice 1998 and all other applicable regulatory requirements.

- 1. Data acquisition and monitoring

Trials unit staff will visit the participating centres to confirm that agreements are being adhered to, specifically to carry out source data verification and confirm compliance with the protocol and the protection of patients' rights as detailed in the Declaration of Helsinki. Copies of the Declaration may be obtained from the designated regional trials unit. By participating in the TRACE RA trial, Principal Investigators at each centre are confirming agreement with his/her local NHS Trust to ensure the following:

- Sufficient data is recorded for all participating patients to enable accurate linkage between patient hospital records and trial case report forms.
- Source data and all trial related documentation are accurate, complete, maintained and accessible for monitoring and audit visits
- All staff at their individual centres who are involved with the trial will meet the requirements of working within the statutory provisions of UK law.
- Original consent forms are dated and signed by both patient and investigator and are kept together in a central log together with a copy of the specific patient information sheet(s) given to the patient at the time of consent. The original consent form must be kept in the centre site file and copies of the consent form should be given to the patient, filed in the patient hospital notes and also forwarded to the regional trials unit.
- Copies of CRFs are retained for 15 years at the NHS Trusts and the University of Manchester to comply with international and organisational regulations
- Staff will comply with the Standard Operating Procedures for TRACE RA

The affiliated trials units will monitor receipt of CRFs, evaluate incoming CRFs for compliance with the protocol and resolve inconsistencies and missing data queries.

Participating centres will be monitored by their allocated trials unit and also possibly by the relevant regulatory authorities. Monitoring by the units will confirm compliance with the protocol and source data verification (SDV).

The Trial Manager (based in Manchester) will establish quality assurance proformas to be completed by the trial co-ordinators when visiting the participating NHS Trusts. Some combined visits between the trials units will be conducted to ensure consistency of approach. Each participating NHS Trust will be visited at least once every six months during the recruitment period and at least annually thereafter to ensure that there is compliance to the protocol and that there is completeness and accuracy of documentation using an established audit trail.

When a monitoring visit is required, the trials unit will contact the centre to discuss proposed visit dates. Once a date has been confirmed, a list of patient initials/ID numbers whose medical records will be monitored/audited will be sent to the recruiting centre. This list will be sent out in advance to give sufficient time for the information to be made available (the trial statistician will provide advice on what percentage of patients are to be monitored/audited).

If any problems are detected in the course of the monitoring/auditing visits, then the Principal Investigator and the trials unit will work together to resolve queries to determine the centre's future participation in the study.

- 1. Data handling and record keeping

All data will be entered on to a computerised database at the trials units. All data will be identified via a unique trial number and data tables will be linked using this number. The names and addresses of patients matched to their trial number will be stored in a separate secure database. All databases will be password protected and stored according to the requirements of the Data Protection Act 1998.

- 1. Archiving

All source and study documentation must be securely retained by the local Principal Investigator for 15 years after the trial has ended. An end of study visit may be performed by the trials units to resolve any data queries and outstanding trial documentation before the documentation can be archived by the participating centre. Source data (including data on any patients who die) must be retained for the duration of the recruitment, treatment and follow up phases of the trial for inspection by representatives of trials units.

- 1. Financial matters

TRACE RA is investigator-designed and led, and is jointly funded by the Arthritis Research Campaign and the British Heart Foundation. Pfizer UK Limited has provided free active trial drug and placebo for the whole of TRACE RA, as well as an unrestricted educational grant for the TRACE RA-DAS sub-study. If additional financial support is received from any other source, this will be made apparent to the approving MREC but will not require a protocol amendment.

Cost implications to the NHS Trusts:

Support for local set-up costs (£300 per centre), local pharmacy set-up and running costs (£200 per centre) and local research nurse time (£70 per recruited patient) will be made available to collaborating NHS Trusts. Invoicing should be raised by the participating trial centres to their respective trials units. The trials centres will be contacted at the end of trial recruitment to ensure that payment has been received by the centre for the services provided. Additional support in terms of human resources will be provided to the trial centres by the trial units, through the trial co-ordinators (for the duration of the trial) and centrally-appointed trial nurses (for the initial 2 years of the trial), subject to satisfactory honorary contracts and reciprocal agreements.

Additional medical or nursing time, the very limited number of laboratory tests and ECGs (additional to the patients' routine care) and any other locality-specific costs are expected to be recovered through the collaborating Trusts' NHS R&D Support for Science allocation (or any mechanisms replacing this).

- 1. Ethical considerations

The trial protocol will be submitted for ethical review to COREC. The trial will NOT commence recruitment until all central and individual site regulatory responsibilities are available.

The main ethical consideration pertaining to the TRACE RA trial is whether the trial should be terminated early if efficacy of atorvastatin is proven. The planned interim analyses should ensure that the trial continues only as long as is needed to establish, beyond reasonable doubt, that treatment with atorvastatin is either beneficial or harmful to patients with RA.

- 1. Publication Policy

The main trial results will be published in the name of the trial in a peer-reviewed journal, on behalf of all collaborators. The trials units and all participating centres and clinicians will be acknowledged in this publication.

All presentation and publications relating to the trial must be authorised by the TRACE RA Trial Steering Committee.

No investigator may, at any time, present or attempt to publish data relating to the TRACE RA trial and its sub studies without prior permission from the Trial Steering Committee.

1. **Dissemination of Results**
   1. Informing trial participants

Participating centres will be sent a list of their patients along with their allocated treatment arm at the end of the trial. They will then be able to inform those patients who wish to know which treatment arm they were allocated.

- 1. Expected value of the results

The proposed trial will add to the evidence base of how to reduce the cardiovascular risk of patients with RA. It will hopefully identify an intervention that will reduce the incidence of the most common cause of death in the most common form of chronic inflammatory arthritis. The trial will also be used to define RA specific CV risk profiles, which can be utilised to identify patients at high risk and inform the need for further investigation and treatment.

Indirectly, this trial may provide some insight into the link between inflammation (in this case, high-grade inflammation) and CVD and provide mechanistic clues that can be investigated specifically in subsequent studies. TRACE RA BioBank will be an excellent resource to address such questions at the basic scientific level.

The TRACE RA-DAS sub-study will add to the evidence base of compounds which have disease modifying properties in RA. If benefit is shown, it is likely that atorvastatin might then be used in combination with other DMARDs to improve overall disease control. This information would be of most benefit in patients with a known high cVd risk.

1. **Confidentiality and Liability**
   1. Confidentiality

The trials units (Dudley, Dundee & Manchester) will comply with all aspects of the Data Protection Act 1998. All information collected during the course of the trial will be kept strictly confidential.

Patients NHS numbers will be collected on a patient eligibility and registration form (this will be returned to Manchester Trials Unit) at the beginning of the trial. All other data collection forms, which are faxed/posted to the trials unit, will be coded with 3 patient identifiers (Patient's Initials, Centre numbers and Patient Identification Number). Information will be held securely on paper and electronically at the trials units, including appropriate storage, restricted access and disposal arrangements of patients' personal and clinical details. Participants will also not be identified in the results of the study.

Patient information recorded in their medical records will be accessed during identification of potential patients and when site monitoring visits occur to ensure that the trial is being carried out according to ICH GCP guidelines.

Stored patient information will be kept on NHS and University computers so as to be able to track the number of patients on the trial. Data from patients' medical records will be transcribed onto case report forms.

All clinical information about patients will be stored on a central database at the University of Manchester. The main database and any sub-databases will be password protected and stored according to the requirements of the Data Protection Act 1998.

On occasion, if quality of life questionnaires are not completed in clinics due to time constraints or patients not attending clinic, the questionnaire may be posted by the research nurse for patients to complete and return to the University of Manchester Trials Unit (a SAE will be provided to facilitate this).

The local principal investigators must keep a separate log of patients' trial numbers, names, addresses and hospital numbers. The local principal investigator must maintain in strict confidence trial documents, which are to be held in the local hospital (e.g. patients' written informed consent forms). The local principal investigator must also ensure that patient confidentiality is maintained.

The trials units will maintain confidentiality of all subject data and will not reproduce or disclose any information by which subjects could be identified, other than reporting of serious adverse events. Representatives of the trials units will be required to have access to patients medical records for quality assurance purposes but patients should be assured that their confidentiality will be respected at all times. This will be stated in the patient information sheet.

- 1. Liability/ Indemnity/ Insurance

The individual NHS trusts have a duty of care to patients treated, whether or not the patient is taking part in a clinical trial and the NHS trusts remain liable for clinical negligence and other negligent harm to patients under this duty of care. Indemnity for participating hospitals is provided by the usual NHS indemnity agreements.

**References**

1. Symmons DP, Turner G, Webb R et al. The prevalence of rheumatoid arthritis in the United Kingdom: new estimates for a new century. Rheumatology 2002; 41: 793-800
2. Goodson N. Coronary artery disease and rheumatoid arthritis. Curr. Opin Rheumatol. 2002; 14: 115-120
3. Kitas GD, Erb N. Tackling ischaemic heart disease in rheumatoid arthritis. Rheumatology 2003; 42: 607-13
4. Kroot EJJA, van Gestel AM, Swinkels HL, et al. Chronic comorbidity in patients with early rheumatoid arthritis: a descriptive study. J Rheumatol 2001; 28: 1511-7
5. Kitas GD, Banks MJ, Bacon PA. Cardiac involvement in rheumatoid disease. Clin Med JRCPL 2001; 1: 18-21
6. Goodson NJ, Solomon DH. The cardiovascular manifestations of rheumatic diseases. Curr. Opin. Rheumatol. 2006; 18(2): 135-40
7. Bacon PA, Raza K, Banks MJ, Townend J, Kitas GD. The role of endothelial cell dysfunction in the cardiovascular mortality of RA. Int Rev Immunol 2002; 21 (1):1 -17
8. Park YB, Ahn CW, Choi HK, Lee SH, In BH, Lee hC et al. Atherosclerosis in rheumatoid arthritis: morphologic evidence obtained by carotid ultrasound. Arthritis Rheum 2002; 46(7):1714-1719.
9. Alkaabi JK, Ho M, Levison R, Pullar T, Belch JJF. Rheumatoid arthritis and macrovascular disease. Rheumatology 2003; 42: 292-7
10. Klocke R, Cockcroft JR, Taylor GJ, Hall IR, Blake DR. Arterial stiffness and central blood pressure, as determined by pulse wave analysis, in rheumatoid arthritis. Ann Rheum Dis 2003; 62(5):414-418
11. Belch, JJF, McArdle B, Madhok R, McLaughlin K, Forbes CD, Sturrock RD. Decreased plasma fibrinolysis in patients with rheumatoid arthritis. Annals of Rheumatic Diseases 1984; 43 (6): 774-779.
12. Lau CS, McLaren M, Hanslip J, Kerr M, Belch JJF. Abnormal plasma fibrinolysis in patients with rheumatoid arthritis and impaired endothelial fibrinolytic response in those complicated by vasculitis. Annals of Rheumatic Diseases 1993; 52 (9): 643-649
13. McEntegart A, Capell HA, Madhok R, Lowe GDO, Rumley A, Woodward M. Cardiovascular (CVS) Risk factors in an RA population. Rheumatology 2000; 39: 40
14. McLaren M, Alkaabi J, Connacher M, Belch JJ, Valenete E. Activated factor XII in rheumatoid arthritis. Rheumatol. Int. 2002; 22(5): 182-4
15. Douglas KM, Pace AV, Treharne GJ, Saratzis A, Nightingale P, Erb N, Banks MJ, Kitas GD. Excess recurrent cardiac events in rheumatoid arthritis patients with acute coronary syndrome. Ann Rheum Dis 2006; 65(3): 348-53
16. Van Doornum S, Brand C, King B, Sunfararajan V. Increased case fatality rates following a first cardiovascular event in patients with rheumatoid arthritis. Arthritis Rheum. 2006; 54 (7): 2061-8
17. Veldhujzen van Zanten JJ, Ring C, Caroll D, Kitas GD. Increased CRP in response to acute stress in patients with rheumatoid arthritis. Ann Rheum Dis. 2005; 64(9): 1299-304
18. Ross R. Atherosclerosis - An inflammatory disease. N Eng J Med 1999; 340: 115-26
19. Kitas GD, Banks MJ, Bacon PA. Accelerated Atherosclerosis as a cause of cardiovascular death in RA. Pathogenesis 1998; 1 (2): 73-83
20. Sattar N, McCarey DW, Capell H, McInnes IB. Explaining how "high-grade" systemic inflammation accelerates vascular risk in rheumatoid arthritis. Circulation 2003; 108(24):2957-2963
21. Stevens R, Douglas KMJ, Saratzis A, Kitas GD. Inflammation and atherosclerosis in rheumatoid arthritis. Exp. Rev. Mol. Med. 2005; 7 (7): 1-24
22. Danesh J, Whincup P, Walker M, Lennon L et al. Low grade inflammation and coronary heart disease: prospective study and updated meta-analyses. Br Med J 2000; 321: 199-204
23. Morrow DA, Ridker PM. C-reactive protein, inflammation and coronary risk. Med Clin N Am 2000; 84: 149-61
24. Goodson NJ, Symmons DPM, Scott DG, Bunn D, Lunt M, Silman AJ. Baseline levels of CRP and prediction of death from cardiovascular disease in patients with inflammatory polyarthritis: a ten year follow up study of a primary care based cohort. Arthritis Rheum 2005; 52(8): 2293-9
25. Wallberg-Jonsson S, Johansson H, Ohman ML, Rantapaa-Dahlqvist S. Extent of inflammation predicts cardiovascular disease and overall mortality in seropositive rheumatoid arthritis: a retrospective cohort study from disease onset. J Rheumatol 1999; 26: 2562-71
26. Jacobsson LT, Turesson C, Hanson RL et al. Joint swelling as a predictor of death from cardiovascular disease in a population study of Pima Indians. Arthritis Rheum. 2001; 44: 1170-6
27. Chehata JC, Hassell AB, Clarke SA et al. Mortality in rheumatoid arthritis: relation to single and composite measures of disease activity. Rheumatology 2001; 40: 447-52
28. Choi HK, Herman MA, Seeger JD, Robins JM, Wolfe F. Methotrexate and mortality in patients with rheumatoid arthritis: a prospective study. Lancet 2002; 359: 1173-7
29. Mitchell DM, Spitz PW, Young DY, Bloch DA, McShane DJ, Fries JF. Survival, prognosis, and causes of death in rheumatoid arthritis. Arthritis Rheum 1986; 29: 706-14
30. Lehtinen K, Isomaki H. Intramuscular gold therapy is associated with long survival in patients with rheumatoid arthritis. J Rheumatol 1991; 18: 524-9
31. Krause D, Schleusser B, Herborn G, Rau R. Response to methotrexate treatment is associated with reduced mortality in patients with severe rheumatoid arthritis. Arthritis Rheum 2000; 43: 14-21
32. Landewe RBM, van den Borne BEEM, Breedveld FC, Djkmans BAC. Methotrexate effects in patients with rheumatoid arthritis with cardiovascular comorbidity. Lancet 2000; 355: 1616-7
33. Erb N, Kitas GD. Homocysteine modulation as a reason for continuous folic acid supplementation in methotrexate-treated rheumatoid arthritis patients. Rheumatology 2001; 40: 715-6
34. Hurliman D. et al. Anti-tumor necrosis factor alpha treatment improves endothelial function in patients with rheumatoid arthritis. Circulation 2002; 106(17): 2184-7
35. Raza K, Banks M, Kitas GD. Reversing myocardial microvascular disease in a patient with rheumatoid arthritis. J Rheumatol 2005; 32(4): 754-6
36. Van Doornum S, McColl G, Wicks IP. Tumour necrosis factor antagonists improve disease activity but not arterial stiffness in rheumatoid arthritis. Rheumatology 2005; 44(11): 1428-32
37. Situnayake RD, Kitas GD. Dyslipidaemia and rheumatoid arthritis. Ann Rheum Dis 1997; 56: 341-2
38. Dessein PH, Joffe BI, Veller MG et al. Traditional and non-traditional cardiovascular risk factors are associated with atherosclerosis in rheumatoid arthritis. J Rheumatol 2005; 32(3): 435-42
39. Alkaabi JK, Ho M, Levison R, Pullar T, Morley KD, Belch JJF. The prevalence of macrovascular disease in Rheumatoid Arthritis (RA) Rheumatology 2000; 39: 39
40. Goodson NJ, Silman AJ, Pattison DL, Lunt M, Bunn D, Luben R, Day N, Khaw KT, Symmons DP. Traditional cardiovascular risk factors measured prior to the onset of inflammatory polyarthritis. Rheumatology 2004; 43(6): 731-6
41. Erb N, Pace AV, Douglas KJM, Banks M, Kitas GD. Risk assessment for coronary heart disease in rheumatoid arthritis and osteoarthritis. Scand. J. Rheumatol. 2004; 33: 293-299
42. Treharne GJ, Hale ED, Lyons AC, Booth DA, Banks M, Erb N, Douglas KM, Mitton DL, Kitas GD. Cardiovascular disease and psychological morbidity among rheumatoid arthritis patients. Rheumatology 2005; 44(2): 241-6
43. HOPE study investigators. Effects of an ACE inhibitor, ramipril, on cardiovascular events in high risk patients. N Eng J Med 2000; 342:145-53
44. Bacon PA, Kitas GD. Rheumatoid Arthritis, Vasculitis and Arteriosclerosis. In: Atherosclerosis and Autoimmunity; Shoenfeld Y, Harats D, Wick G (eds); Elsevier Science BV 2001; 301-13
45. Adhiyaman V, Asghar M, Oke A, White AD, Shah IU. Nephrotoxicity in the elderly due to co-prescription of angiotensin converting enzyme inhibitors and nonsteroidal anti-inflammatory drugs.J R Soc Med. 2001; 94(10):512-4
46. Downs JR, Clearfield M, Weis S et al for the AFCAPS/TexCAPS Research Group. Primary prevention of acute coronary events with lovastatin in men and women with average cholesterol levels. JAMA 1998; 279: 1612-22
47. Shepherd J, Cobbe SJ, Ford I et al for the West of Scotland Coronary Prevention Study Group. Prevention of coronary heart disease with pravastatin in men with hypercholesterolaemia. N Eng J Med 1995; 333: 1701-7
48. Scandinavian Simvastatin Survival Study Group. Randomised trial of cholesterol lowering in 4444 patients with coronary heart disease. The Scandinavian Simvastatin Survival Study (4S). Lancet 1994; 344: 1383-9
49. Sacks FM, Pfeffer MA, Moye LA et al. The effect of pravastatin on coronary events after myocardial infarction in patients with average cholesterol levels. N Eng J Med 1996; 335: 1001-9
50. Long-term Intervention with Pravastatin in Ischaemic Disease (LIPID) Study Group. Prevention of cardiovascular events and death with pravastatin in patients with coronary heart disease and a broad range of initial cholesterol levels. N Eng J Med 1998; 339: 1349-57
51. Plehn JF, Davis BR, Sacks FM et al. Reduction of stroke incidence after myocardial infarction with pravastatin. The cholesterol and recurrent events (CARE) study. Circulation 1999; 99: 216-23
52. Leung BP, Sattar N, Crilly A et al. A novel anti-inflammatory role for simvastatin in inflammatory arthritis. J. Immunol. 2003; 420: 78-84
53. Blake GJ, Ridker PM. Are statins anti-inflammatory? Curr Control Trials Cardiovasc Med 2000; 1: 161-5
54. Palinski W, Napoli C. Unravelling pleiotropic effects of statins on plaque rupture. Arterioscl Thromb Vasc Biol 2002; 22: 1745-50
55. Kwak B, Mulhaupt F, Myit S, Mach F. Statins as a newly recognised type of immunomodulator. Nat Med 2000; 6: 1399-1402
56. Weitz-Schmidt G. Statins as anti-inflammatory agents. TRENDS in Pharmacol Sci 2002; 23 (10): 482­486
57. Gotto AM Jr. Antioxidants, statins and atherosclerosis. J Am Coll Cardiol. 2003; 41 (7): 1205-10
58. Kitas GD, Sattar N. The potential role of statins in the treatment of rheumatoid arthritis. J. R. Coll. Physicians Edinb. 2005; 35: 309-16
59. Douglas KMJ, Sattar N, Kitas GD. Potential role of statins and PPARs in rheumatoid arthritis. Future Rheumatology 2006; 1(2): 259-274
60. McCarey DW, McInnes IB, Madhok R, Hampson R et al. Trial of atorvastatin in rheumatoid arthritis (TARA): double-blind, randomised, placebo-controlled trial. Lancet 2004; 363: 2015-21
61. Colhoun HM, Thomason MJ, Mackness MI, Maton SM et al. Design of the collaborative atorvastatin diabetes study (CARDS) in patients with type 2 diabetes. Diabetic Medicine 2002; 19: 201-11 (results just presented in abstract form)
62. Koren MJ, Hunninghake DB & the ALLIANCE Investigators. Clinical outcomes in managed care patients with coronary heart disease treated aggressively in lipid-lowering disease management clinics. The ALLIANCE study. J. Am. Coll. Cardiol. 2004; 44: 1772-9
63. Cannon CP, Braunwald E, McCabe BS, Rader DJ et al. Comparison of intensive and moderate lipid lowering with statins after acute coronary syndromes. N. Eng. J. Med. 2004; 350
64. Kinlay S, Timms T, Clark M, Karam C et al. Comparison of effect of Intensive lipid lowering with atorvastatin to less intensive lowering with lovastatin on C-reactive protein in patients with stable angina pectoris and inducible myocardial ischemia. Am. J. Cardiol. 2002; 89: 1205-7
65. van Wissen S, Trip MD, Smilde TJ, de Graaf J et al. Differential hs-CRP reduction in patients with familial hypercholesterolaemia treated with aggressive or conventional statin therapy. Atherosclerosis 2002; 165: 361-6
66. Topol EJ. Intensive statin therapy - A sea change in cardiovascular prevention. N. Eng. J. Med. 2004; 350
67. Williams B, Poulter NR, Brown MJ, Davis M et al. British Hypertension Society guidelines for hypertension management 2004 (BHS-IV): summary. Brit Med J 2004; 328: 634-40
68. Schwartz GG, Olsson AG, Ezekowitz Md, Ganz P et al. Effects of atorvastatin on early recurrent ischemic events in acute coronary syndromes. The MIRACL study: a randomized controlled trial. JAMA 2001; 285: 1711-8
69. Van Doornum S, McColl G, Wicks IP. Atorvastatin reduces arterial stiffness in patients with rheumatoid arthritis. Ann Rheum Dis. 2004; 63(12): 1571-5
70. Klareskog L, Hamsten A. Statins in rheumatoid arthritis: two birds with one stone? Lancet 2004; 363: 2011-1
71. The American Rheumatism Association 1987 revised criteria for the classification of rheumatoid arthritis. Arthritis Rheum 1988;31:315-24
72. Kirwan JR, Reeback J. Stanford health assessment questionnaire modified to assess disability in British patients with rheumatoid arthritis. Br J Rheumatol 1986;25:206-9
73. EuroQol Group. EuroQol—a new facility for the measurement of health-related quality of life. Health Policy 1990;16:199-208.
74. Prevoo MLL, Van't Hof MA, Kuper HH et al. Modified disease activity scores that include twenty-eight joint counts Arthritis Rheum 1995; 38: 44-8
75. Witney AG, Treharne GJ, Tavakoli M, Lyons AC, Vincent K, Scott DL, Kitas GD. The relationship of medical, demographic and psychosocial factors to direct and indirect health utility instruments in rheumatoid arthritis. Rheumatology 2006; 45(8): 975-81
76. Shephard J, Blauw GJ, Murphy MB, Cobbe SJ et al. The design of a Prospective Study of Pravastatin in the Elderly at Risk (PROSPER).

A1.1 Study Hypothesis

- Atorvastatin is more effective than placebo as adjuvant therapy for the control of disease activity in patients with RA
- Atorvastatin is more effective than placebo as adjuvant therapy in slowing radiographic damage and the long-term decline in physical function in patients with RA
- RNA samples will allow gene expression profiling studies in RA. Blood samples collected from this patient population would facilitate research in a broader array gene expression profiling approach and would allow assessment of differential expression of genes without a priori information.

A1.2 Study Design

A 3-year, multicentre, randomised, double blind, placebo-controlled trial of atorvastatin 40mg once daily for control of disease activity in patients with RA aged >50 years. This is a sub-study of the main TRACE RA trial which explores the influence of atorvastatin on cardiovascular endpoints.

A total of 326 patients will be required and recruitment to the disease activity sub-study will be completed within 12 months of study commencement and each patient will be followed for two years (with respect to the sub-study but will be followed up for another 3 years until the end of the cardiovascular/main study).

The inclusion and exclusion criteria for the disease activity sub-study are similar to the main study with the express proviso that all patients entering the sub-study must have a baseline DAS28 score of >4.4.

A1.3 Proposed Duration of Treatment, Follow up and Frequency of Intervention

These patients will have an additional nurse global assessment, X-ray of hands and wrist at the randomisation visit and be asked to provide a blood sample for RNA testing to be carried out the University of Dundee. A Repository Committee will be set up to oversee work relating to these samples and to consider requests from other organisations for the blood samples. All requests from academic or pharmaceutical organisations to use the samples would be submitted to an ethics committee for regulatory approval.

Patients will also have the following assessment in Months 6, 12, and 24:

28 tender and swollen joint counts Patient global assessment Nurse global assessment

X-ray of hands and wrists (at baseline and month 24 only)

ESR

A1.4 Proposed Sample Size

A sample size of 326 patients is required to provide 80% power at the 5% significance rate to detect a difference in EULAR response rate between statin treated and the placebo arms. This assumes a 20% response rate in the placebo arm and 20% loss to follow-up during the 2 years of the sub-study

A1.5 Planned Analysis

All patients randomised in the trial will be included in the analysis, irrespective of whether the study drug was discontinued (ITT analysis). EULAR response rates in the two arms will be compared using odds ratios with 95% confidence intervals and adjusting for any baseline differences between the groups. Rheumatology efficacy outcomes will be recorded at 6, 12 and 24 months visits. For safety outcomes, logistic regression models will be used for analysis with treatment allocation together with the factors used for stratification. Any further baseline variables can be incorporated into the model if necessary to adjust for baseline imbalances. Two-sided p values of <0.05 will be considered significant.

A1.6 Planned Sub-group Analysis

Sub-group analyses will be carried out with respect to: gender, age group (<65 vs. >65 years), RA disease duration, rheumatoid factor status and anti TNF therapy (if number if sufficient).

A1.7 Proposed Frequency of Analysis

No interim analysis is planned for TRACE RA DAS. Safety data will be continuously monitored by the independent DMC.

A2.1 Background

Rheumatoid Arthritis (RA) was previously considered to be a benign, controllable disease with a reasonably good prognosis in the majority of patients. It is now known that it can be a severe, progressive disorder associated with a premature mortality. Patients with RA have a 5-year mortality rate similar to that of patients post myocardial infarction (MI), with triple coronary vessel disease or with neoplastic disease, RA should be viewed as an urgent medical problem requiring immediate intervention. Despite this, research targeted to cardiovascular disease (CVD), the main cause of death in RA, was ignored for many years. In RA life span is shortened by approximately 15-20% from the date of onset of the illness.

Between 34-40% of the excess deaths are from cardiovascular causes. Various studies have given Standard Mortality Ratios for CVD in RA of between 1.51 to 5.25, where the lower estimate is for MI only and the largest includes heart failure. The TRACE RA study addresses the clinical problem of enhanced CV mortality in this group of patients via a randomised controlled trial (RCT) of Atorvastatin versus placebo in 3,808 RA patients. This study is funded by the arc and the British Heart Foundation and will provide novel data on the beneficial, or otherwise, effects of a statin on CV events and mortality in RA.

However, such a trial, where the study population is large and well characterised, also provides unique opportunities for major studies, particularly those where DNA and RNA can be used to shed light on disease susceptibility, outcome and drug responsiveness. Familial, twin, admixture and migration studies all indicate a substantial contribution of genetic factors in the aetiology of this common complex disorder. Substantial advances in gene identification are now being made: linkage scans have highlighted consistently replicated regions harbouring susceptibility genes and a growing number of substantiated susceptibility variants have been defined. Further progress in definition and characterisation of novel genes will require a large community-based RA DNA resource, enriched by the availability of detailed long-term clinical follow-up.

This resource will allow: study of the modest relative risks expected of many susceptibility variants (by comparison with 'normal' populations), replication of other resources, measurement of the population attributable risk of susceptibility variants, evaluation of potential gene/gene and gene/environment interactions, definition of complication-related susceptibility variants, and exploration of genetic effects on treatment response. There is, therefore, a clear case to be made for establishing such a resource on the basis of genetic studies alone.

In addition the study of inflammation and CV disorders is evolving, in particular biomarkers for disease progression and events are becoming available, and more will be uncovered. A resource of banked serum and plasma from a well-phenotyped large population has the potential to answer questions relating to these novel mechanisms in a rapid, powered and convincing fashion.

A2.2 Study Aims

- To develop a DNA repository for the 3808 patients with rheumatoid arthritis (RA) enrolled in the main TRACE RA study
- To develop a plasma and serum repository for the above patients.
- To devise and complete peer-reviewed projects on the above samples using the phenotypic and outcome data collected in the TRACE RA trial.
- To take advantage of established population-based record-linkage capability, to allow longitudinal tracking of the subjects, thereby enabling investigation of the relationship between susceptibility genotypes and pertinent clinical features including characteristics at diagnosis, response to treatment, development of RA complications, vascular disease and survival.
- To develop an infrastructure surrounding the collection, storage and use of these samples which concerns itself with ethics, consent, privacy and collaboration, to protect the rights of the patients whilst maintaining the highest standards of clinical research.

A2.3 Methodology:

Informed consents and blood will be collected from 3,808 patients with RA that are participating in the main TRACE RA study.

1. Subjects:

The subjects will be those enrolled into the TRACE RA study (inclusion and exclusion criteria described in TRACE RA protocol). 3,808 RA patients will be enrolled in up to 100 centres.

1. Sample handling and DNA extraction:

The following blood samples will be taken from each subject: One 10mls citrate sample (for plasma), one 10mls clotted (for serum), and two 10mls EDTA for DNA (immediately stored at -80C). Serum and plasma will be prepared, aliquoted and frozen at the collection site in an appropriately timely fashion. We will also store blood spots from each subject for future use (whole genome amplification; identity confirmation etc). Frozen samples will be transported in batches on dry ice via courier in specially prepared packages routinely used by the group for sample transfer.

Transfer to Ninewells Hospital will be followed by further appropriate sample preparation, and storage in alarmed secure freezers. DNA for all samples will be quantified by fluorescence dye techniques to maximise standardisation of concentrations, an essential prerequisite to robust, quality high throughput genotyping.

Finally, each sample will be tested in a multiplex PCR reaction that will include an SRY marker (a Y- specific amplicon, as a check on identity integrity) and one autosomal marker (such as beta-actin) (to confirm DNA quality). DNA quantification, normalisation, dilution and aliquoting will be performed using an Xiril X100 liquid handling robot (available in Dundee), and stored at -30^0^c in bar coded replicate 96 well plates Importantly, DNA samples will be bar-coded and the robot will confirm the identity of each sample and track the progress of each sample to all daughter plates.

A2.4 Projects and Statistics:

A Repository Committee will be set up to oversee the work relating to these samples, and to consider requests from other organisations. This committee will consist of the 3 Principal Investigators, the co­applicant, the arc selected Trial Steering Committee chairman amongst others including patient representation. This steering group would become the custodian of the DNA resource to ensure that maximum benefit is derived from the collection whilst avoiding any unnecessary duplication of genotyping/assaying. All requests from academic or pharmaceutical organisations to use the samples would be submitted to an ethics committee for regulatory approval.

The mainstay of analyses based on this resource will be using conventional genetic/serum/plasma statistical methodology, encompassing a range of different analytical approaches and study objectives (including for example, single-locus and haplotype-based studies; biological and positional candidate analyses; replication studies for associations uncovered in other (smaller) populations; logistic regression analyses seeking to dissect LD structure and define aetiological variants; and gene-gene interactions). In addition, the associated longitudinal clinical data will permit analyses of the relationship between genotypes of interest and pertinent outcomes including complication rates; treatment response; survival/mortality; and disease progression. These data will be analysed using multivariate regression and Cox regression methods, including algorithms for repeated measures when appropriate.

A2.5 Facilities and Experience available for the study:

Dundee's Ninewells Hospital is uniquely placed to act as the physical Repository; due to its experience in such studies to date. Dr Colin Palmer and Professor Jill Belch have extensive experience in sample preparation for these types of studies.

These include grants from TENOVUS to pilot endothelial studies in a large population of patients with diabetes, the Raynaud's and Scleroderma Association to link the development of carotid vascular disease to certain genotypes in Systemic sclerosis, from the Welcome Trust UK Case/control study of type 2 diabetes, where all subjects (n=15000) are from Tayside. Tayside is also the principal centre for the recruitment and sample handling for Generation Scotland, with 10,000 individuals to be targeted within the next 2 years, growing to 50,000 Scotland wide by the end of 2011. The robotics and software developed for these large studies will easily accommodate the currently proposed sample throughput.

POPADAD, a Scotland wide 8 year CV mortality trial (n= 1,320) also has Ninewells (and the Dundee applicants) as its core laboratory for sample collection. In addition we have recently developed a "High Volume" genotyping workflow to complement our "high throughput" affymetrix and Illumina systems, where we can cheaply and efficiently genotype 10,000 patient samples for 1 SNP in 2 hours. This system uses both KASPAR indirect allelic discrimination and TAQMAN based allelic discrimination. KASPAR is simpler and much cheaper, but assays that fail on KASPAR will be performed on TAQMAN. Both genotyping techniques are homogenous fluorescent assays that can be performed easily in an automated fashion. Sample reformatting will be performed to 384 well formats on a Xiril liquid handling robot. Reaction mixtures will be dispensed to 384 using a DEERAC high speed nanodispenser (1-2ul total reaction volumes). PCR will be performed in a H2OBIT ultra-high-throughput thermal cycler (10,000 samples per 2 hour run) and the final genotypes scored using an Applied Biosystem SDS9700. The genotyping project will be analysed, managed and quality controlled using a dedicated genotyping database system (KLUSTERCALLER, KBiosciences).

Standard analytical techniques, in addition to genetic studies, include HPLC-MS, GC-MSMS, ELISA, Spectrophotometry, Affymetrix micro-array, MALDI, proteomics (Prof Mike Ferguson), flow cytometry, (Luminex Bead array) and Western Blotting.

A2.6 Ethical considerations

a. Confidentiality, security, ethics and database management

With the implementation of the new data protection act issues of data security and confidentiality are very much in the public eye, as they should be. The mechanisms employed to ensure appropriate security and confidentiality within Ninewells result from years of deliberation and debate and are continually reviewed and updated in parallel with legal and ethical requirements. The exemplar project for Ninewells is the DARTS project, (Diabetes Audit and Research Tayside). As an example of how TRACE RA samples will be treated using DARTS logistics will be explained below.

Clinical information on DARTS is stored on a Structured Query Language (SQL) database. Clinical information is accessed via a Web server and Web browser. The first level of security on clinical data is that imposed by the NHS-NET network. Although DARTS has a presence on the wider Internet, users from the Internet cannot access the DARTS Web Server located within the confines of NHS-NET. A login screen is presented and communications are all encrypted, and access determined by username and password. All attempts to log-on to DARTS and every action subsequently taken are logged producing audit trails. For DARTS anonymous Genetic research we use all the security and confidentiality mechanisms provided by the Microsoft SQL Server V7 platform.

The Tenovus grant award has allowed us to implement a secure system for anonymous genetic case/control studies. Every patient, GP, general practice and hospital clinic represented on the system is allocated a unique randomly generated identifier intended for the purposes of anonymous research. Researchers are then restricted to 'views' of data that use these anonymous identifiers and omit any identifying fields.

Thus, all demographic information is removed except sex, age (in months), and social class (Carstair's Index). For genetic research, only the nurse performing the fieldwork has access to the patients identifying numbers. This nurse does not have access to any other patient-specific data. In addition, although the system administrator (through necessity) has access to all data on the server, the identity of individual genetic markers is not available to him.

The present information sheet is included in Appendix 3. It has been modified according to MRC guidelines to state that products derived from the sample may be used by other researchers and the commercial sector. It includes specific clauses that allow use of samples for RA and wider medical issues including drug response.

All record linkage studies and issues relating to confidentiality, anonymisation of data and dataset security will come under the aegis of the Health Informatics Centre at the University of Dundee. This has an external scientific Advisory Board (chair Professor Elizabeth Russell, Professor of Epidemiology, University of Aberdeen) that scrutinises all anonymised record linkage research in Tayside according to published standard operating procedures.

1. Administration of the Collection

DNA aliquots will be made and master stocks stored in Dundee. Complete plated collections of the DNA (~15ug) will be distributed to the appropriate collaborating centres. Requests for DNA from external collaborators will be considered. Written applications will be to test specific variants within a gene and standardized data will be required in these requests: data to establish the importance of the gene, the role of the specific variants to be typed e.g. coding variants, haplotype tagging SNPs etc, details of the assay used for SNPs, preliminary data to give allele frequencies and power calculation. Permission will be given to exclusively (for one year after permission is granted) test the requested specific SNPS.

DNA aliquots may be obtained from Dundee. DNA will be given blinded and will include approximately 10% of samples with the most DNA as duplicates to provide a measure of genotyping accuracy. A condition of using the resource will be that all genotyping will be given to the core database so that these data are held centrally; the specific data field however will remain under the custodianship of the scientist performing the study until the data are published.

The analysis of the ongoing longitudinal data will be obtained by users of the resource working with the epidemiological team in Dundee. A collaborative framework has been established in the recent SRIF- funded Health Informatics Centre at Ninewells Hospital. The Health Informatics Centre thus has standard operating procedures that facilitate this collaborative inter-institutional research on anonymised datasets for approved projects. In order to ensure the continuation and the development of this resource, a cost recovery charge will be levied for the supply of DNA and subsequent data analysis to external centres from Dundee. Pricing will be determined by the Repository committee based on cost recovery and will be aligned with other UK resources. We would use these funds to support the maintenance and development of the resource at Ninewells beyond the period of this award.

A2.7 Conclusion

We anticipate great demand both nationally and internationally because, to our knowledge there is no comparable publicly available resource.

It will be especially unique because of the longitudinal follow-up of phenotypic data. This Repository will form the basis of an RA Framingham and provide insight into disease mechanisms, complications and drug responsiveness for many years to come.

**APPENDIX 3: SAMPLE PATIENT INFORMATION SHEET, CONSENT FORMS AND GP LETTERS**

These are provided as separate documents and will be stored in the master trial file, regional trial unit site files and the centres' site files.

Please see list of documents as follows (All documents will be approved by MREC):

1. Patient Information Leaflet & Consent - Main TRACE RA trial
2. Patient Information Leaflet & Consent - TRACE RA DAS (Disease Activity sub-study)
3. Patient Information Leaflet & Consent - TRACE RA BioBank (Genetic sub-study)
4. Patient Information Leaflet & Consent - ONS Flagging (for non-eligible patient/ eligible patients who do not wish to enter the main trial)
5. GP letter
6. GP letter for ONS Flagging consent from non-participants
7. Promotional Leaflet - TRACE RA information to patients
8. Promotional Leaflet - TRACE RA information to investigators

**APPENDIX 4: QUESTIONNAIRES**

These are provided as separate documents and will be stored in the master trial file, regional trial unit site files and the centres' site files.

Please see list of documents as follows (All documents will be approved by MREC):

1. Lifestyle questionnaire (Version 1, 01 November 2006)
2. Health Assessment Questionnaire (HAQ)
3. EQ5D - EuroQul 5 dimensional lifestyle questionnaire
4. Patient Global Assessment (Visual Analogue Score)
5. Nurse Global Assessment (Visual Analogue Score) - (TRACE RA DAS Substudy only)

**APPENDIX 5: DEFINITIONS OF CARDIOVASCULAR ENDPOINTS**

An independent cardiovascular endpoint classification committee will review cardiovascular events and all

deaths and will classify them according to the WHO MONICA method, or alternative of their choice.

Co-primary Endpoints

- Cardiovascular death, non-fatal myocardial infarction or stroke^76^
- As above plus coronary and carotid revascularization

Secondary endpoints

- All cause mortality
- Fatal and non-fatal peripheral atherosclerotic events
- Carotid revascularization
- Hospitalised angina

A5.1 PRIMARY CARDIOVASCULAR ENDPOINTS

1. Stroke (any event that meets the criteria listed below for one of the following 3 categories of stroke):

1. Ischemic stroke (one of the following conditions must be met):
2. Rapid onset of focal neurologic deficit lasting >24 hours or leading to death plus evidence from neuroimaging (CT or MRI) showing cerebral/cerebellar infarction or no abnormality, or post-mortem examination showing cerebral and/or cerebellar infarction.
3. Rapid onset of global neurologic deficit (e.g. coma) lasting >24 hours or leading to death plus evidence from neuroimaging showing infarction, or post-mortem examination showing infarction.
4. Focal neurologic deficit (mode of onset uncertain) lasting >24 hours or leading to death plus evidence from neuroimaging showing infarction, or post-mortem examination showing infarction.
5. Primary intercerebral and/or cerebellar haemorrhage (one of the following conditions must be met):
6. Rapid onset of focal neurologic deficit lasting >24 hours or leading to death plus evidence from neuroimaging or post-mortem examination showing primary intracerebral and/or cerebellar haemorrhage.
7. Rapid onset of global neurologic deficit (e.g. coma) lasting >24 hours or leading to death, plus evidence from neuroimaging or post-mortem examination showing primary intracerebral and cerebellar haemorrhage.
8. Focal neurologic deficit (mode of onset uncertain) lasting >24 hours or leading to death, plus evidence from neuroimaging or post-mortem examination showing primary intracerebral and/or cerebellar haemorrhage.
9. Not known (1 of the following conditions must be met):
10. Rapid onset of focal neurologic deficit lasting >24 hours or leading to death, without neuroimaging or post-mortem data available.
11. Rapid onset of global neurologic deficit (e.g. coma) >24 hours or leading to death, without neuroimaging or post-mortem data available.
12. Focal neurologic deficit (mode of onset uncertain) lasting >24 hours or leading to death, without neuroimaging or post-mortem data available.

Note: The following conditions will be excluded from the defined endpoint of stroke as outlined above:

1. Primary subarachnoid haemorrhage
2. Subdural or extradural haematoma
3. Traumatic intracerebral haemorrhage
4. Neurologic deficit due to major metabolic or haemodynamic disturbance
5. Venous sinus thrombosis and
6. Cerebral tumour
7. Transient ischaemic attack:

a. Rapid onset of focal neurologic deficit or loss of monocular function lasting < 24hours.

Note: Isolated rotational vertigo, diplopia, or dysphagia are excluded from the defined endpoint of transient ischaemic attack.

1. Definite coronary heart disease death (either or both of the following criteria must be met):
2. Death certificate or equivalent documentation with consistent or underlying or immediate cause plus:
3. Preterminal hospitalisation with definite or suspect myocardial infarction
4. Previous definite angina or suspect or definite myocardial infarction when no other cause other than atherosclerotic coronary heart disease death could be ascribed as the cause of death and
5. Autopsy evidence of acute coronary arterial thrombosis and/or acute myocardial infarction
6. Sudden and unexpected death (requires all 3 characteristics):
7. Death occurring within 1 hour after onset of symptoms or having been last been seen without them
8. No known nonatherosclerotic acute or chronic process or event that could have been potentially lethal.
9. An 'unexpected' death in a person who is not confined at home, hospital, or other institution because of illness within 24 hours before death.
10. Definite nonfatal myocardial infarction (1 or more of the following criteria must be met):
11. Diagnostic electrocardiogram (ECG) at the time of the event
12. Ischaemic cardiac pain (and/or unexplained acute left ventricular failure) and diagnostic enzymes.
13. Ischaemic cardiac pain and/or unexplained acute left ventricular failure with both equivocal enzymes and equivocal electrocardiogram
14. Diagnostic enzymes and equivocal electrocardiogram.
15. Angiographic evidence of occlusion of a major artery with appropriate ventriculographic wall motion abnormality where previous angiogram since randomisation showed no such abnormality.
16. An electrocardiogram at an annual or at an unscheduled visit showing a myocardial infarction that was not evident on the previous electrocardiogram.
17. Suspect coronary heart disease death (either or both of the following criteria must be met):
18. Death certificate or equivalent documentation with consistent underlying or immediate cause but neither adequate preterminal documentation of the event nor previous atherosclerotic coronary heart disease diagnosis.
19. Rapid and unexpected death (all 3 characteristics required):
20. Death occurring between 1 and 24 hours after the onset of severe symptoms or having last been seen without them.
21. No known nonatherosclerotic acute or chronic process or event that could have been potentially lethal.
22. An 'unexpected' death in a person who is not confined to the home, hospital, or other institution because of illness within 24 hours before death.
23. Suspect nonfatal myocardial infarction (any 1 of the following criteria is met but the combination of (a) and (b) or (a) and (c) is not present):
24. Ischaemic cardiac pain and/or unexplained acute left ventricular failure.

Note: This definition holds only if an electrocardiogram performed during or shortly after the pain shows new equivocal changes as defined in this appendix, or alternatively, enzymes estimated around the time of pain are equivocal.

1. Diagnostic enzymes
2. Equivocal electrocardiogram and equivocal enzymes
3. Other cardiac death: Death certificate or equivalent document with consistent underlying or immediate cause and adequate preterminal documentation of the event.

Note: Preterminal documentation and/or information may include hospitalisation for, or diagnosis of, a cardiac-related illness other than atherosclerotic heart disease for a previous event other than the terminal event.

1. Other vascular death: Death certificate or equivalent documentation with consistent underlying or immediate cause and adequate preterminal documentation of the event.

Note: Preterminal documentation and/or information may include hospitalisation for, or diagnosis of, a vascular-related illness for a previous event other than the terminal event.

1. Other death (either of the following criteria must be met) *:
2. Death certificate or equivalent documentation with diagnosis consistent with preterminal documentation and/or information.
3. Death certificate only

- When no formal written documentation is available, verbal information from relative and/or witness will be admissible and should be recorded on the appropriate study forms.

1. Coronary artery bypass graft surgery (CABG)

Other coronary artery revascularization procedures (angioplasty, atherectomy, laser ablation or stenting or any newly introduced invasive method for the management of coronary artery disease).

A5.2 SECONDARY ENDPOINTS

1. All-cause mortality
2. Fatal peripheral atherosclerotic events

Death occurring within 28 days from the onset of hospital-verified acute peripheral arterial embolic events. The diagnosis is based on symptoms, clinical findings and/or appropriate radiological investigations.

1. Non-fatal peripheral atherosclerotic events

Hospital-verified acute peripheral arterial embolic events. The diagnosis is based on symptoms, clinical findings and/or appropriate radiological investigations.

1. Carotid revascularization
2. Hospitalised angina

The Endpoints Committee will be responsible for the classification of all possible study endpoints. The Committee will receive all baseline and end of trial electrocardiograms showing serial changes, information regarding domiciliary visits or hospitalisation associated with possible myocardial infarction, and information on all deaths.

**APPENDIX 6: SAFETY OUTCOMES**

Clinical laboratory safety parameters are shown below. Haematology and biochemistry will be performed at the local laboratories. All of the safety parameters are subject to routine regular monitoring for safety of the disease-modifying anti-rheumatic drugs (DMARDs. Such monitoring is required as part of routine clinical practice at intervals ranging between 2 weeks and 3 months for all but one DMARDs (the antimalarials). All patients will be questioned about adverse events at each follow-up visit, and both serious and non-serious adverse events will be recorded and made available for review by the DMC. Serious adverse events (SAE) will be communicated within 24 hours to the main co-ordinating centre and then to the DMC. If a SAE occurs, it is at the discretion of the local investigator to interrupt or discontinue the study drug. Randomisation codes will be kept securely by participating pharmacists. If deemed necessary by the local investigator the randomisation code can be broken either by contacting the randomisation centre of the local pharmacy.

Abnormalities of haematological and biochemical parameters in this population may be either due to DMARDs or the study drug. The decision of attribution will be left to the managing local investigator.

The drug should be stopped if:

- Elevation of ALT or AST to >3 times ULN persisting on retesting 1 week later should lead to discontinuation of study medication and coded as such.
- If there is elevation of CK to more than 10 times ULN the drug should be stopped immediately. Elevation of CK to >5 times ULN persisting on retesting 1 week later should lead to discontinuation of study medication and coded as such.

Additional assessments for the purpose of the study:

Patients will be required to undergo an ECG at baseline and at the end of the trial to monitor their heart function and patients will also be asked to provide a blood sample at baseline to measure the RhF to confirm that they suffer from rheumatoid arthritis.

**APPENDIX 7: INFORMATION ABOUT ATORVASTATIN**

Atorvastatin is generally well-tolerated. Adverse reactions have usually been mild and transient. Less than 2% of patients were discontinued from clinical trials due to side effects attributed to Atorvastatin.

The 'Summary of Product Characteristics' about Atorvastatin will be filed in the TRACE RA site file. Information about Atorvastatin can be found on the electronics medicines compendium section of the ([www.emc.medicines.org.uk](http://www.emc.medicines.org.uk)) website and detailed information about the side effects of the drug is also available in the British National Formulary (BNF - Current version. 51, March 2006).

Clinical Adverse Experiences (Please see ‘Summary of Product Characteristics’ for more details)

The most frequent (1% or more) adverse effects associated with Atorvastatin therapy, in patients participating in controlled clinical studies were:

Psychiatric disorders - Insomnia

Nervous System disorders - Headache

Gastrointestinal disorders - Abdominal pain, dyspepsia, nausea, flatulence, constipation, diarrhoea Musculoskeletal and Connective Tissue Disorders: Myalgia

General Disorders and Administration Site Conditions:

- Asthenia
- Elevated serum ALT levels have been reported in patients receiving Atorvastatin.
- Elevated serum CPK levels > 3 times upper normal (ULN) occurred in 2.5 % of patients on Atorvastatin compared with 3.1% with other HMG-CoA reductase inhibitors in clinical trials.

[Additional adverse events that have been reported in atorvastatin clinical trials are categorised below according to system organ class and frequency. Frequencies are defined as: very common (>10%), common (>1% and <10%), uncommon (>0.1% and <1%), rare (>0.01% and <0.1%) and very rare (0.01%)].

| **Organ/System Disorder** | **Event** | **Frequency** |
| --- | --- | --- |
|  | Anorexia | Uncommon |
| Metabolism & Nutrition | Hypoglycaemia  Hyperqlycaemia | Very rare Very rare |
|  | Dizziness | Common |
| Nervous system | Paresthesia | Uncommon |
|  | Peripheral neuropathy | Rare |
| Gastrointestinal | Vomiting  Pancreatitis | Uncommon  Rare |
| Hepatobiliary | Hepatitis  Cholestatic jaundice | Very rare Very rare |
| Skin & Subcutaneous Tissue | Alopecia  Pruritus | Uncommon  Uncommon |
|  | Rash | Uncommon |
| Musculoskeletal and Connective Tissue | Muscle cramps  Myositis  Myopathy | Uncommon Rare Very rare |
| Reproductive system | Impotence | Uncommon |
|  | Chest pain | Common |
| General disorders | Angina | Common |
|  | Angioneurotic oedema | Very rare |

**APPENDIX 8: POWER CALCULATION AND OTHER STATISTICAL CONSIDERATIONS**

Table 1

Total sample size required for logistic regression (after 5 years, assuming total drop-out rate of 20%)

Reduction in Annual Event Rate

Annual event rate Power

|  | 1.8% | 1.9% | 2.0% | 1.8% | 1.9% | 2.0% |
| --- | --- | --- | --- | --- | --- | --- |
|  | 80% | 80% | 80% | 90% | 90% | 90% |
| 30% | 4368 | 4150 | 3948 | 5778 | 5490 | 5224 |
| 31% | 4090 | 3870 | 3666 | 5410 | 5118 | 4848 |
| 32% | 3808 | 3616 | 3438 | 5036 | 4780 | 4544 |
| 33% | 3580 | 3360 | 3206 | 4734 | 4444 | 4236 |
| 34% | 3348 | 3154 | 3018 | 4424 | 4166 | 3986 |
| 35% | 3136 | 2966 | 2826 | 4140 | 3914 | 3730 |

Total sample size required for Cox regression (after 5 years, assuming a total drop-out rate of 20%)

Reduction in Annual Event Rate

| Annual event rate | 1.8% | 1.9% | 2.0% | 1.8% | 1.9% | 2.0% |
| --- | --- | --- | --- | --- | --- | --- |
| Power | 80% | 80% | 80% | 90% | 90% | 90% |
| 30% | 4100 | 3888 | 3696 | 5488 | 5204 | 4948 |
| 31% | 3810 | 3612 | 3436 | 5100 | 4836 | 4598 |
| 32% | 3548 | 3364 | 3200 | 4750 | 4504 | 4282 |
| 33% | 3310 | 3140 | 2984 | 4432 | 4202 | 3996 |
| 34% | 3094 | 2934 | 2790 | 4142 | 3926 | 3734 |
| 35% | 2896 | 2746 | 2612 | 3876 | 3676 | 3496 |

Total number of patients with events required for Cox regression

Reduction in Annual Event Rate

Annual event rate Power

|  | 1.8% | 1.9% | 2.0% | 1.8% | 1.9% | 2.0% |
| --- | --- | --- | --- | --- | --- | --- |
|  | 80% | 80% | 80% | 90% | 90% | 90% |
| 30% | 244 | 243 | 243 | 326 | 326 | 325 |
| 31% | 225 | 225 | 225 | 301 | 301 | 301 |
| 32% | 208 | 208 | 208 | 279 | 279 | 278 |
| 33% | 193 | 193 | 193 | 259 | 258 | 258 |
| 34% | 180 | 180 | 179 | 240 | 240 | 240 |
| 35% | 167 | 167 | 167 | 224 | 223 | 223 |

**APPENDIX 9: COMPOSITION AND ROLE OF TRIAL STEERING COMMITTEE AND DATA MONITORING COMMITTEE**

A11.1 TRACE RA Trial Steering Committee (TSC) – Composition

The Chief Investigator (CI) must obtain written approval of the TSC composition from arc before their first meeting. The membership should be limited and include an independent Chairman (not involved directly with the trial other than as a member of the TSC), two or more other independent expert members and the Chief Investigator. Where possible the membership should include a lay/consumer representative. Caroline Dore, the arc CTC Senior Statistician, who is based at the MRC Clinical Trials Unit, should be invited to all meetings, in the role of observer. The trial manager, trial statistician etc should attend meetings as appropriate. Observers from arc and Host Institution should be invited to all meetings.

Chairman: Professor Gordon Lowe, Professor of Vascular Medicine, Glasgow Royal Infirmary

Independent Members:

Dr Jane Armitage, Senior Research Fellow & Honorary Consultant, CTSU, University of Oxford Professor Keith Fox, Professor of Cardiology, University of Edinburgh Centre for Cardiovascular Science Professor Dorian Haskard, Director, Eric Bywaters Centre for Vascular Inflammation, Imperial College London

ARC member:

Ms Caroline Dore, Senior Clinical Trial Statistician, ARC Lay member:

Ms Ailsa Bosworth, Chief Executive, National Rheumatoid Arthritis Society Investigators:

Professor George Kitas, Consultant Rheumatologist, Dudley Group of Hospitals NHS Trust Professor Jill Belch, Professor of Cardiovascular and Inflammation Medicine & Honorary Consultant Physician, Institute of Cardiovascular Research, Ninewells Hospital Professor Deborah Symmons, Consultant Rheumatologist, University of Manchester

Secretary: Ms Sumitra Smith, Clinical Trials Manager, University of Manchester

A11.2 TRACE RA Trial Steering Committee (TSC) -Role of the Trial Steering Committee:

It is arc Clinical Trials Collaboration policy that a Trial Steering Committee (TSC) should be set up for each of its multi-centre trials with the following terms of reference:

Terms of Reference:

1. To monitor and supervise the progress of the trial towards its interim and overall objectives
2. To review at regular intervals relevant information from other sources (e.g. other related trials)
3. To consider the recommendations of the Data Monitoring Committee (DMC)
4. In light of 1, 2 & 3, to inform arc on the progress of the trial
5. To advise arc on publicity and the presentation of all aspects of the trial.

The TSC provides overall supervision for the trial, and advice to the CI, arc and the Host Institution on all aspects. The first meeting of the TSC should be held before recruitment to the trial begins, in order for the TSC to approve the protocol. Thereafter, the TSC should meet at least annually. The TSC should ensure that there are no major deviations from the trial protocol. The CI should call meetings of the TSC when there are any matters arising from the conduct or management of the trial that might require their advice.

A11.3 Data Monitoring Committee (DMC) - Composition

The Central DMC assigns a sub-group consisting of at least three members to represent clinical, statistical and clinical trial expertise to each trial funded by arc CTC. Each sub-group will have a Chair to organise work related to their specific trial. All members of the sub-group should be independent of the trial they are monitoring. The frequency with which the DMC sub-group meets will be dependent on the needs of the individual trial. The Chief Investigator (CI) should submit a detailed plan for the interim analysis before the trial commences. The plan must satisfy members of the DMC sub-group. Communication between the CI and the sub-group chair is encouraged but should not bypass the TSC Chair.

The CI and the Chair of the TSC will agree with their DMC sub-group Chair a timely mechanism for reporting to the DMC sub-group. With the help of the trial statistician, the CI must provide blinded data, in strict confidence, to the DMC sub-group as frequently as the members of the sub-group request. The template for reporting interim data should be used by all CIs. Serious unexpected suspected adverse reactions must be reported to the lead clinician of the DMC sub-group and chairperson of the relevant multi-centre research ethics committee immediately. If appropriate, the MHRA (Medicines and Healthcare products Regulatory Agency) must also be informed.

Chairman: Professor Howard Bird, Professor of Pharmacology and Rheumatology, University of Leeds

Members:

Dr Christopher Edwards, Consultant Rheumatologist, University of Southampton Dr Hazel Inskip, Senior Scientist, Deputy Director of MRC Epidemiology Resource Centre

A11.4 Data Monitoring Committee (DMC) - Role

The DMC is the only body involved in the trial that has access to the unblinded comparative data. The role of the DMC is to monitor these data and make recommendations to the TSC on whether the trial should continue. Membership of the DMC should be completely independent of the CI, TSC and Host institution. The arc Clinical Trials Collaboration has established a central DMC, and will select a DMC sub-group for each arc funded multi-centre trial, assigning members from this central pool. The first meeting of the DMC should be held before recruitment to the trial begins, in order for the DMC to approve the protocol and ensure that appropriate arrangements have been made for review of the accumulating data and the results of any interim analyses.

Terms of Reference: DMC sub-group:

1. To set up and maintain direct communication with the CI and Chair of the TSC. The Chair of the TSC should be made aware of all communications between the CI and DMC sub-group.
2. To receive a copy of the trial protocol and plans for interim analysis prior to commencement of the trial, or, in the case of the first wave of trials, as early as possible.
3. To receive reports (as per template in Appendix 3) during the trial at intervals agreed with the TSC and CI. It would be expected that these would be 6 monthly in the first year, and no less frequent than 12 monthly after that.
4. If interim analysis of the trial data is not planned in the protocol the sub-group should determine whether interim analysis should be undertaken
5. To consider data from interim analyses, unblinded if considered appropriate, plus any additional safety issues for the trial and relevant information from the template and other sources
6. In the light of 3, 4 & 5, and ensuring that ethical considerations are of prime importance, to report to the Central DMC and TSC and recommend on the continuation of the trial.

**APPENDIX 10: TRIAL EVALUATIONS SCHEMA**

* DAS - TRACE RA DAS REQUIREMENTS

** VISIT 9 - MOST PATIENTS WOULD FINISH TRIAL AT VISIT 8, HOWEVER, PATIENTS WHO WERE RECRUITED IN THE EARLY QUARTER OF THE STUDY WOULD BE MONITORED FOR UP TO 72 MONTHS AND WOULD HAVE MONTH 72 TO AS THEIR FINAL VISIT, INSTEAD OF MONTH 60.

| Trial Investigations | Visit 1 | | Visit 2 | Visit 3 | Visit 4 | | Visit 5 | | Visit 6 | Visit 7 | Visit 8 | Visit 9 | EOT |
| --- | --- | --- | --- | --- | --- | --- | --- | --- | --- | --- | --- | --- | --- |
|  | Baseline | | Month 3 | Month 6 | Month 12 | | Month 24 | | Month 36 | Month 48 | Month 60 | Month 72 | Month 60/72 |
|  | ALL | DAS | ALL | DAS | ALL | DAS | ALL | DAS | ALL | ALL | ALL | ALL | ALL |
| Inclusion/Exclusion Criteria Assessments Written Informed Consent  Height, Weight, BP, Waist Circumference  Medical History  Concomitant Medication  Routine Haematology/Biochemistry  ESR/CRP  RHF  Glucose  Creatinine Kinase, LFTs Research plasma, serum and DNA sample  Research RNA sample ECG  X-ray of hands and wrist Discussion of CVD risks  HAQ  EQ5D  Lifestyle Questionnaire  28 Tender & Swollen joint count Patient Global Assessment (VAS)  Nurse Global Assessment (VAS)  Check for Adverse events Check for Cardiovascular events Drug compliance |  |  |  |  |  |  |  |  |  |  |  |  |  |
|  | ✓ | # |  |  |  |  |  |  |  |  |  |  |  |
|  | ✓ | # |  |  |  |  |  |  |  |  |  |  |  |
|  |  |  |  |  |  |  |  |  |  |  |  |  |  |
|  | ✓ |  |  |  |  |  |  |  |  |  |  |  | ✓ |
|  | ✓ |  |  |  |  |  |  |  |  |  |  |  |  |
|  | ✓ |  | ✓ |  | ✓ |  | ✓ |  | ✓ | ✓ | ✓ | ✓ | ✓ |
|  | ✓ |  |  |  | ✓ |  | ✓ |  | ✓ | ✓ | ✓ | ✓ | ✓ |
|  | ✓ |  |  | # |  | # |  | # |  |  |  |  | ✓ |
|  | ✓ |  |  |  |  |  |  |  |  |  |  |  | ✓ |
|  | ✓ |  |  |  |  |  |  |  |  |  |  |  |  |
|  | ✓ |  |  |  |  |  |  |  |  |  |  |  |  |
|  | ✓ |  |  |  |  |  |  |  |  |  |  |  |  |
|  |  | # |  |  |  |  |  |  |  |  |  |  |  |
|  |  |  |  |  |  |  |  |  |  |  |  |  |  |
|  | ✓ |  |  |  |  |  |  |  |  |  |  |  | ✓ |
|  |  | # |  |  |  |  |  | # |  |  |  |  |  |
|  | ✓ |  |  |  |  |  |  |  |  |  |  |  |  |
|  |  |  |  |  |  |  |  |  |  |  |  |  |  |
|  | ✓ |  |  |  | ✓ |  | ✓ |  | ✓ | ✓ | ✓ | ✓ |  |
|  | ✓ |  |  |  | ✓ |  | ✓ |  | ✓ | ✓ | ✓ | ✓ |  |
|  | ✓ |  |  |  |  |  |  |  |  |  |  |  |  |
|  |  |  |  |  |  |  |  |  |  |  |  |  |  |
|  | ✓ |  |  | # |  | # |  | # |  |  |  |  | ✓ |
|  | ✓ |  |  | # |  | # |  | # |  |  |  |  | ✓ |
|  |  | # |  | # |  | # |  | # |  |  |  |  |  |
|  |  |  |  |  |  |  |  |  |  |  |  |  |  |
|  |  |  | ✓ |  | ✓ |  | ✓ |  | ✓ | ✓ | ✓ | ✓ |  |
|  |  |  | ✓ |  | ✓ |  | ✓ |  | ✓ | ✓ | ✓ | ✓ |  |
|  |  |  | ✓ |  | ✓ |  | ✓ |  | ✓ | ✓ | ✓ | ✓ |  |

**Supplementary_Methods_3: TRIAL HISTORY**

When TRACE-RA was designed the two co-primary endpoints were i) cardiovascular death, non-fatal myocardial infarction or stroke; and ii) these endpoints plus coronary revascularisation. The anticipated event rate for the first endpoint was 1⋅8% per annum and the planned sample size was 3808 to be followed for five years with an expected 20% non-compliance. If the true effect of taking the treatment was a 32% RRR but only 75% of patients were compliant, then the effect seen would be about 24% (i.e. 75% of 32%). The minimum number of first events to be able to detect such a reduction with 80% power at the 5% significance level was calculated to be 208. With an annual event rate in the placebo arm estimated at 1⋅8%, it was calculated that around 27000 person years (PYRs) of follow-up were required (supplementary figure 1). A major review of the trial was undertaken in May/June 2010 by the funders, sponsors and the Trial Steering Committee (TSC), about three years after recruitment commenced (August 2007). The observed event rate at that time was below the anticipated 1⋅8% at 1⋅3%, which was partly attributed to a “healthy volunteer” effect. Revised power calculations suggested that an expansion of the primary endpoint and increase in sample size would be required to achieve adequate statistical power. As a result Ethics committee approval was obtained to modify the primary endpoint to also include non-coronary revascularisation and TIAs and to exclude haemorrhagic stroke and non-coronary cardiac death anticipating an event rate of 1⋅6% pa; and to increase the sample size and recruitment period from 3808 in two years to 5350 patients to be recruited by March 2014. The aim was to accrue 434 (instead of the originally calculated 208) primary events after five years of follow-up. This required an increase in the number of recruiting sites from 60-100 to up to 120 centres (supplementary figure 1). The protocol was revised to include these changes and follow-up was to continue until approximately 27000 person years of follow-up had accrued.

However, even with the expansion of the primary endpoint, the event rate remained even lower (0⋅7% pa) than initially observed and expected. As a result, TRACE RA was terminated prematurely in December 2012 on the grounds of futility, as the funders deemed it unlikely that the trial would fulfil its main aims. Results presented include data collected until December 2012 by which time 2⋅5 years of median follow-up had accrued.

**Supplementary_Methods_4 – TSC APPROVED STATISTICAL ANALYSIS PLAN**

Overview and plan of investigation

This document contains a comprehensive description of the statistical techniques to be used in the analysis of data from the Trace RA study and has been written prior to data lock.

Study design and randomisation

Trace RA is a multi-centre, randomised, double blind, placebo-controlled trial of oral atorvastatin 40mg once daily versus placebo (dummy atorvastatin tablets) once daily.

Patients, investigators and trial units are unaware of the treatment allocation. The medication is provided by Pfizer UK Ltd., bottled by Catalent Pharma Solutions UK Limited and dispensed by the local study pharmacist. The randomisation process is incorporated into the drug labelling. Each patient is allocated a filled and labelled bottle coded with a unique drug number when entering the trial. All future supplies for this patient will be coded with the same unique drug number. Patients will continue on the same treatment (i.e. active or placebo) throughout the duration of the study. Scratch cards are supplied to the local pharmacy so that the code can be broken if necessary. Catalent Pharma Solutions provides the independent trial statistician with information about treatment allocation by unique drug number to enable the interim analyses to be conducted.

Primary objective

The primary aim of this study is to determine whether atorvastatin is more effective than placebo in the primary prevention of cardiovascular events in RA patients. A cardiovascular event is defined as:

Coronary event;

Presumed ischaemic stroke or transient ischaemic attack (TIA);

Any non-coronary arterial revascularisation;

Any other cardiovascular death excluding both confirmed cerebral haemorrhage and non-coronary cardiac death

Secondary and tertiary objectives

The secondary aim of this study is to determine whether atorvastatin is more effective than placebo in the prevention of the components of the primary endpoint separately:

Coronary event (non-fatal myocardial infarction, coronary death or coronary revascularisation);

Presumed ischaemic stroke or TIA;

Any non-coronary arterial revascularisation;

Any other cardiovascular death excluding both confirmed cerebral haemorrhage [ICD I64-99 in the 10th International Classification of Diseases]) and non-coronary cardiac death [ICD I00-I15 and I26-I52]

The tertiary aim of the study is to determine whether the difference in the effectiveness of atorvastatin and placebo in the primary prevention of cardiovascular events in RA patients varies by subgroup. Subgroup analyses will be performed for:

Age

Gender

DAS28

Rheumatoid factor

Other tertiary aims of the study are to compare the effects of atorvastatin and placebo with regard to:

Total and cause-specific mortality (coronary, other vascular and non-vascular death separately)

Hospitalisations for various other causes

Statin safety-related outcomes (persistent elevation of ALT or AST; myopathy, defined as muscle symptoms plus CK>10 x upper limit normal)

Lipid levels

Functional outcome assessed by HAQ and EQ5D

Determination of sample size

The aim of the trial is to detect (with 80% power at the 5% significance level) a 32% relative risk reduction (RRR) in the primary endpoint attributable to treatment with atorvastatin and anticipating an average of 25% non-compliance. If the true effect of taking the treatment is a 32% RRR but only 75% of patients are compliant, then the effect seen in this trial should be about 24% (i.e. 75% of 32%). The required number of first events (i.e. patients with events) to be able to detect such a reduction with 80% power at the 5% significance level is 434 (Appendix Three). The estimated average blinded event rate in all patients is expected to be 1⋅6-1⋅8% per annum. A maximum of around 27000 patient-years of follow-up is required for these event rates to produce the required number of first events (Appendix Three).

Study plan

See Trial Schema (page six of protocol) and Trial Evaluations Schema (Appendix 10 of protocol, page 58).

Patients are asked to attend visits at three months, and then six-monthly from randomisation. When attendance is not possible the case report form may be completed by telephone interview. If patients are to continue study treatment, preferably ALT (and if ALT not available then AST) measurements are mandatory within six weeks of the follow-up visit.

Modifications to the statistical section of the protocol

The following are all recorded as amendments to the study protocol (Appendix 11 of protocol, page 59).

The independent data monitoring committee queried the p value of <0⋅0005 that was suggested in the protocol as a guide as to when the trial should definitely be discontinued. Since there is no formal stopping rule for this study, it was not intended that p values > 0⋅0005 should be used as justification for continuing the trial. It was assumed that the data monitoring committee would consider all the available evidence when making such a decision. The p value of <0⋅0005 was removed from the protocol to avoid misinterpretation.

The number of recruiting centres was increased from 100 to 120, with a prediction that the required number of patients would be recruited by March 2014.

Compliance to study treatment will be measured by asking the patient instead of by tablet count.

Power calculations and other statistical calculations were amended in light of statistical remodelling of trial statistics based on observed recruitment rates in the first three years and observed event rates during this period.

Demographic and baseline characteristics

Demographic variables will include gender, age and ethnicity.

Baseline characteristics will include body mass index, smoking status, time since diagnosis, time since onset of symptoms, tender joint count, swollen joint count, early morning stiffness, DAS28, pain score, fatigue score, wellness score, SDI score, ADI score, EQ5D VAS, systolic blood pressure, diastolic blood pressure, hypertension, first degree relative with the disease, family history of diabetes, medications, ESR, CRP, RhF (rheumatoid factor), anti-CCP (anti-cyclic citrullinated peptide antibody), haemoglobin, platelets, white blood count, neutrophils, creatinine, AST, ALT, glucose, total cholesterol, triglycerides, HDL, and LDL.

Primary efficacy variable

The records of English and Welsh randomised patients will be electronically tagged for mortality with the Office for National Statistics (ONS) and Scottish patients at the Scottish Office’s Information and Statistics Division (ISD) as well as the local hospital Trusts’ Medical Information departments. Underlying cause of death will be ascertained from death certificates provided by ONS and ISD (which also supply details of times and causes of all hospital admissions in Scotland) supplemented by information from hospital records, including post-mortem examinations, if performed.

Hospital admissions will be ascertained at each centre by matching the patient details with national NHS Hospital Episode Statistics. In addition, non-fatal events will be ascertained regularly by contact with each patient. If necessary, additional information will be sought from household members, GP or hospital departments.

An independent Endpoints Committee will be responsible for the classification of all possible study endpoints. The Committee will receive all baseline and end of trial electrocardiograms showing serial changes, information regarding domiciliary visits or hospitalisation associated with possible endpoints, and information on all deaths.

Secondary and tertiary efficacy variables

The secondary efficacy variables will be the components of the primary endpoint separately:

Coronary events [i.e. non-fatal myocardial infarction, coronary death or coronary revascularisation];

Presumed ischaemic stroke or transient ischaemic attack;

Any non-coronary arterial revascularisation;

Any other cardiovascular death excluding both confirmed cerebral haemorrhage [ICD I64-99 in the 10th International Classification of Diseases]) and non-coronary cardiac death [ICD I00-I15 and I26-I52]

The tertiary efficacy variables will be:

Total and cause-specific mortality (coronary, other vascular and non-vascular death separately)

Hospitalisations for various other causes

Statin safety-related outcomes (persistent elevation of ALT or AST; myopathy, defined as muscle symptoms plus CK>10 x upper limit normal)

Lipid levels

Functional outcome assessed by HAQ and EQ5D

Adverse events

A Serious Adverse Event (SAE) is defined as any untoward medical occurrence that:

- results in death
- is life-threatening (i.e. with an immediate, not hypothetical, risk of death at the time of the event),
- requires hospitalisation or prolongs existing hospitalisation
- results in persistent or significant disability or incapacity,
- is a congenital anomaly or birth defect (i.e. the outcome of pregnancy involving the patient)
- is any other important medical condition which, though not included in the above, may jeopardise the patient and may require medical or surgical intervention to prevent one of the outcomes listed

Medical judgement should be exercised in deciding whether an adverse event/reaction is serious in other situations. Hospitalisations include planned admissions for elective surgery. All serious adverse events must be reported on the serious adverse event form.

An adverse event is the development of an undesirable medical condition or the deterioration of a pre-existing medical condition following or during exposure to a pharmaceutical product, whether or not considered causally related to the product. An undesirable medical condition can be symptoms (e.g. nausea, chest pain), signs (e.g. tachycardia, enlarged liver) or the abnormal results of an investigation (e.g. laboratory findings, electrocardiogram).

Adverse events (either serious or non-serious) which lead to discontinuation of study treatment should be routinely recorded on the CRF. Other adverse events need not be recorded in the CRF.

In addition, new significant muscle symptoms should be sought at each study visit and recorded. If present and if ALT is greater than the upper limit of normal, then a blood CK should be measured.

Serious adverse events, judged by the reporting investigator as having a reasonable causal relationship to atorvastatin, qualify as serious adverse reactions. This judgement of causality should be made without breaking the randomisation code – on the assumption that the patient has been exposed to atorvastatin. Serious adverse reactions (SARs) should be reported on the serious adverse event report form.

For any reported SAR, the assessment of ‘expectedness’ will be made by the Chief Investigator (or their delegated deputy) based on the current Summary of Product Characteristics (SmPC)/Package Insert for atorvastatin. If confirmed as a suspected unexpected serious adverse reaction (SUSAR) it will be subject to expedited reporting by the trial sponsor (i.e. Chief Investigator and University of Manchester) to MHRA, therefore every effort should be made to notify the regional trials office within the specified timeframe (Section 9⋅7 of protocol, page 23). As a general rule, the treatment code for the specific patient should be broken before reporting a SUSAR to MHRA. Events associated with placebo will usually not satisfy the criteria for a SUSAR.

Laboratory safety variables

Clinical laboratory safety parameters are shown below. Haematology and biochemistry will be performed at the local laboratories. All of the safety parameters are subject to routine regular monitoring for safety of the disease-modifying anti-rheumatic drugs (DMARDs). Such monitoring is required as part of routine clinical practice at intervals ranging between two weeks and three months for all but one DMARDs (the antimalarials). All patients will be questioned about muscle symptoms at each follow-up visit and if new and significant, CK will be measured. Only serious adverse events will be recorded. Abnormalities of haematological and biochemical parameters in this population may be either due to DMARDs or the study drug. The decision of attribution will be left to the managing local investigator.

The study drug should be stopped if:

- Patient has new and significant muscle pains and CK>10xULN,
- Patient has new and significant muscle pains and ALT/AST >2xULN and CK between 3-10xULN and elevation persists upon retesting.
- Myopathy is diagnosed (muscle pain or weakness and CK >10xULN)
- Elevation of CK to >5xULN, persisting on retesting one week later, should also lead to discontinuation of study medication.

Vital signs

N/A (only measured at baseline)

Other safety variables

N/A

(Health related) Quality of life variables

Functional outcome assessed by HAQ and EQ5D recorded annually since randomisation

Health economic variables

The trial will not address any economic issues directly but, since EQ5D can be used for health utility assessment, the results can subsequently be used for economic modelling and cost-effectiveness based on calculation of QALYs gained. The trial will also monitor the number of hospital admissions and length of stay that occur via information collated from the CRFs.

Efficacy population

All efficacy analyses will be based on all randomized patients irrespective of whether the patient actually received study drug or the patient’s compliance with the study protocol.

Safety population

All safety analyses will be based on randomized and treated patients. This population corresponds to all randomized patients who received at least one study drug administration (either atorvastatin or placebo).

Disposition of patients

The number and percentage of all randomized patients and randomized and treated patients will be summarized by centre and by treatment group.

A listing of randomized and non-treated patients will be presented with patient number, date of consent, centre and the main reason for not being treated.

Patients prematurely permanently discontinuing the study drug will be summarized by treatment group (using counts and percentages) according to the main reason for discontinuation as described in the CRF.

Patients who did not complete the study will be considered as lost to follow-up and summarized in each treatment group.

The number and percentage of patients whose code has been broken on site will be summarized by treatment group according to the reason for code breaking.

Statistical methods

All the available follow-up data will be used in the analysis and all tests will be two-sided at the 5% level. Unless otherwise specified, all the safety analyses will be purely descriptive. Continuous data following a Normal distribution will be summarized using the number of observations available (n), mean, and standard deviation; other continuous data will be summarized using n, median, lower quartile and upper quartile; categorical data will be summarized using counts and percentages. Missing data will not be categorized in the summaries and will not be taken into account in the denominator. Descriptive analysis of continuous and categorical efficacy data will focus on endpoints defined as the last non-missing post-baseline values. In addition, descriptive statistics by visit will be provided on observed cases, i.e. only on patients having non-missing assessments at a nominal visit. The reference dates will be date of first double-blind study drug administration if applicable or date of consent otherwise. In all tables and listings the reference date(s) will be clearly identified. Computed duration expressed in days will be calculated by subtracting the two selected dates plus one day.

Demographics and baseline characteristics

All of the analyses will be performed on all randomised patients and on all randomised and treated patients. All baseline characteristics will be summarized by treatment groups with descriptive statistics and statistical comparisons will be performed.

Smoking status will be analysed using the categories current smoker (smokes more than one cigarette per day), ex-smoker (has smoked more than one cigarette per day but does not currently) and non-smoker (has never smoked more than one cigarette per day).

Medications (taken at baseline or concomitantly to the study drug) will be classified into one of the following categories by the investigator:

Non-biological Disease Modifying Anti-Rheumatic Drugs (DMARDs)

Biological DMARDs (Anti-TNFs)

Steroids

Aspirin

Non-steroidal anti-inflammatory drugs (NSAIDs) / Coxibs

ACE Inhibitors

Non-study statin

Other cardiac drugs

Concomitant medications are medications starting between the date of first study drug administration and the date of last study drug intake (both extreme dates included).

Extent of study treatment exposure and compliance

Summaries of compliance data will be provided for all randomised patients. Compliance will be estimated based on patient reports on Case Report Forms and patients will be considered compliant if they report taking ‘most’ of their study tablets since their last visit.

Analysis of primary efficacy variable

The primary comparison will involve Cox regression analyses of “major vascular events” (as defined above) during the scheduled treatment period among all those allocated atorvastatin tablets versus all those allocated placebo tablets (i.e. “intention-to-treat” comparisons) stratified by centre.

Cumulative incidence functions in each treatment group will be calculated and plotted using nonparametric Kaplan-Meier estimates. Corresponding 95% confidence intervals will be computed at each scheduled time-point of the protocol using Greenwood’s variance estimate. Hazard ratios with 95% confidence intervals (CI) will be estimated using the Cox model. Patients without events in the study period will be considered as right-censored at the date of their final follow-up visit (whether face-to-face or telephone) or at 20^th^ December 2012 for those without a final follow-up visit. Final follow-up visits are defined as those visits that took place between 26^th^ March 2012 and 20^th^ December 2012. The number of each component defining the primary endpoint will be summarized.

A multivariable analysis will be performed using a Cox proportional hazards model stratified by centre and adjusting for any baseline imbalances between treatment groups. A second analysis will adjust for compliance and statin use (as time-dependent dichotomous variables) also.

The Kaplan-Meier survival curves will be compared between treatment groups using the Tarone-Ware test as a sensitivity analysis.

Analysis of secondary and tertiary efficacy variables

The secondary efficacy variables defined above will be analysed in the same way as the primary efficacy variable.

Total and cause-specific mortality (coronary, other vascular and non-vascular death separately) will also be analysed in the same way.

The number of hospitalisations for various other causes will be compared and the causes summarised by treatment group.

Statin safety-related outcomes (persistent elevation of ALT or AST; myopathy, defined as muscle symptoms plus CK>10 x upper limit normal) in the two treatment groups will be compared separately and combined.

Lipid levels at the end of the study in the two treatment groups will be compared. For patients with baseline lipid levels recorded, the change in levels from baseline to end of study will be calculated and the changes in the two groups will be compared.

Functional outcome assessed by HAQ and EQ5D in the two treatment groups will be compared at each time point.

Multiplicity issues

Allowance for multiple hypothesis testing in these analyses will be made using the “Bonferroni” correction.

Analyses of adverse events

Adverse events that are components of the primary endpoint will be excluded from these analyses. The following information will be displayed as an overview of adverse events:

- Number (%) of patients with any SAE,
- Number (%) of patients permanently withdrawn from treatment due to AE (i.e. the AE was indicated by investigators as the main reason for premature discontinuation and the action taken due to the AE was permanent discontinuation of study drug)
- Summary tables by system organ class and preferred term/high level group term
- Summary of relationship to study drug (possibly related/not possibly related

according to the investigator) and maximal intensity (mild/moderate/severe), respectively. In case of missing information concerning relationship to study drug, it will be considered as possibly related (worst case scenario).

Laboratory variables analysis

Summary by visit:

- Laboratory parameters and their changes from baseline will be summarized using means and standard deviations, or medians and quartiles, at each time-point and for the last measure assessed before the end of the study period. Only patients with baseline value for the parameter considered will be taken into account.

Analyses of vital sign variables

N/A (only measured at baseline)

Analyses of other safety variables

N/A

Analyses of (health related) quality of life variables

Health related quality of life: EQ-5D questionnaire (EUROQOL )

- The responses of each EQ-5D item will be presented by visit for each treatment group using tables. The tables will contain information on the frequency and proportion of the population reporting level 1 (no

problems), level 2 (some problems) and level 3 (extreme problems) per item, by treatment group. Only patients with baseline and a post baseline assessment will be considered in the analysis. EQ-5D self-reported VAS data generates a quantitative measure on the self-perceived overall health related quality of life. The VAS will be described by visit with the mean and the standard deviation for each treatment group.

HAQ scores will be presented by visit for each treatment group.

Analyses of health economic variables

The number and duration of hospitalisations will be summarized in each treatment group

Missing data

Unless otherwise stated, missing values will remain missing, i.e. no attempt will be made to impute missing values, and only observed values will be used in data analyses and presentations.

Derived variables will be considered missing if the original variables required to calculate them are missing. For example, if a baseline assessment is missing for a particular patient, then change from baseline at endpoint will be missing. Depending upon the assessment, analyses may not include all patients in the analysis population, because certain patients in the intended population may have missing data.

In the analyses of efficacy variables at the final evaluation, the LOCF procedure will be used to account for incomplete assessments for patients who discontinue the study prematurely and for missing final assessments for patients who complete the study.

If the assessment of the relationship to study drug is missing, then a possible relationship to the study drug will be assumed and the AEs will be listed as such in the frequency tables of possibly related AEs.

Windows for time points

The reference day in the study will be the day of the first double-blind study drug administration and will be denoted by Day 1. If a patient is randomized but not exposed to double-blind study drug, the reference day will be the date of consent.

No time windows will be defined for visits; only nominal times will be used in the definition of time points.

Unscheduled visits

Data from unscheduled visits will be treated in the same way as data from scheduled visits. Unscheduled visits will be categorised as due to an SAE, due to a raised ALT/AST ratio or due to another reason. For this categorisation an SAE will take precedence over a raised ALT/AST ratio, which will take precedence over other reasons.

Interim analysis

That performed for DMC

Software documentation

All summaries and statistical analyses will be generated using PASW Statistics 18 or a later version of SPSS

**Appendices**

Appendix 1 Study flow chart (page six of protocol)

Appendix 2 Summary of analyses

| **Type of Analysis** | **Analysis Population(s)** | **Statistical Method** |
| --- | --- | --- |
| Disposition of patients | Efficacy and safety populations | Counts and percentages by centre and by treatment group. |
| Demographics and baseline characteristics | Efficacy and safety populations | Descriptive statistics (means and standard deviations, medians and quartiles or counts and percentages) by treatment group; comparisons of treatment groups using t tests, Mann Whitney tests, Fisher’s exact test or Kendall’s tau-b as appropriate. |
| Compliance | Efficacy population | Numbers and percentages compliant at each nominal time point by treatment group. |
| Statin use | Efficacy population | Numbers and percentages on either the study statin or a non-study statin at each nominal time point by treatment group. |
| Primary efficacy variable | Efficacy population | Cox regression analyses stratified by centre; hazard ratios with 95% confidence intervals from Cox model. Kaplan-Meier estimates with 95% confidence intervals at each nominal time-point using Greenwood’s variance estimate; Tarone-Ware test to compare treatment groups. Counts of each component defining the primary endpoint by treatment group.  Multivariable analysis using Cox model stratified by centre and adjusting for any baseline imbalances between treatment groups.  Multivariable analysis using Cox model stratified by centre, adjusting for any baseline imbalances between treatment groups and adjusting for compliance and statin use as time-dependent dichotomous covariates. |
| Secondary and tertiary efficacy variables | Efficacy population | Subgroup analyses of the primary efficacy variable will be performed in the same way as the main analysis, but with the inclusion of an interaction between treatment group and subgroup.  The secondary efficacy variables, as well as total and cause-specific mortality (coronary, other vascular and non-vascular death separately), will be analysed in the same way as the primary efficacy variable.  The number of hospitalisations in the two treatment groups for various other causes will be compared using Kendall’s tau-b and the causes summarised as counts and percentages by treatment group.  The numbers of patients with statin safety-related outcomes (persistent elevation of ALT or AST; myopathy, defined as muscle symptoms plus CK>10 x upper limit normal) in the two treatment groups will be compared separately and combined using Fisher’s exact test.  Lipid levels at the end of the study in the two treatment groups will be compared using t tests or Mann Whitney tests, as appropriate. For patients with baseline lipid levels recorded, the change in levels from baseline to end of study will be calculated and the changes in the two groups will be compared using t tests or Mann Whitney tests, as appropriate.  Functional outcome assessed by HAQ and EQ5D in the two treatment groups will be compared at each nominal time-point using t tests or Mann Whitney tests, as appropriate. |
| Adverse events | Safety population | Counts and percentages of patients with serious adverse events by treatment group. |
| **Type of Analysis** | **Analysis Population(s)** | **Statistical Method** |
| Laboratory variables | Safety population | Descriptive statistics (means and standard deviations or medians and quartiles) at each nominal time-point by treatment group; descriptive statistics (means and standard deviations or medians and quartiles) of changes from baseline at each nominal time-point by treatment group. |
| Health related quality of life | Efficacy population | Counts and percentages reporting level 1 (no problems), level 2 (some problems) and level 3 (extreme problems) for each EQ-5D item at each nominal time-point, by treatment group; descriptive statistics (means and standard deviations or medians and quartiles) of EQ-5D self-reported VAS at each nominal time-point for each treatment group; descriptive statistics (means and standard deviations or medians and quartiles) of HAQ scores at each nominal time-point for each treatment group. |
| Health economic variables | Efficacy population | Number of hospitalisations in each treatment group; descriptive statistics (means and standard deviations or medians and quartiles) for duration of hospitalisations in each treatment group. |

Efficacy population: all randomized patients irrespective of whether the patient actually received study drug or the patient’s compliance with the study protocol

Safety population: all randomized patients who received at least one study drug administration (either atorvastatin or placebo)

**Supplementary_Figure_1: Number of events required for 80% power at different levels of event rate and relative risk reduction.**

RR: risk reduction; ER: event rate

**Supplementary Figure 2: TRACE RA results in the context of the Cholesterol Treatment Trialists’ meta-analysis of statin trials**

Proportional reduction of CVE risk per 1mmol/L LDLc reduction in TRACE RA in comparison with the Cholesterol Treatment Trialists’ (CTT) Collaboration meta-analysis of 26 statin trials (reference 11). CI: confidence interval; CVE: cardiovascular event; LDL: low density lipoprotein

**Supplementary_Table_1: Detailed Trial Profile during Follow-up**

**Total (N=3002)**

**Atorvastatin (N=1504)/Placebo (N=1498)**

| **TIME** | **PRIMARY FATAL** | | | | | **PRIMARY NON-FATAL** | | | **OTHER DEATHS** | | | | | **END OF TRIAL VISIT** | | | | **WITHDREW CONSENT** | | **DID NOT ATTEND SPECIFIED FOLLOW UP VISIT** | | **DUE TO ATTEND NEXT SPECIFIED**  **VISIT** | | | |
| --- | --- | --- | --- | --- | --- | --- | --- | --- | --- | --- | --- | --- | --- | --- | --- | --- | --- | --- | --- | --- | --- | --- | --- | --- | --- |
| **BASELINE** | **0** | | | | | **0** | | | **0** | | | | | **0** | | | | **0** | | **0** | | **3002** | | | |
|  | **0** | | **0** | | | **0** | | **0** | **0** | | **0** | | | **0** | | **0** | | **0** | **0** | **0** | **0** | | **1504** | **1498** | |
|  | | | | | | | | | | | | | | | | | | | | | | | | | |
| **3 MONTHS** | **0** | | | | | **7** | | | **1** | | | | | **0** | | | | **29** | | **57** | | **2908** | | | |
|  | **0** | | **0** | | | **0** | | **7** | **0** | | **1** | | | **0** | | **0** | | **17** | **12** | **28** | **29** | | **1459** | **1449** | |
|  | | | | | | | | | | | | | | | | | | | | | | | | | |
| **6 MONTHS** | **0** | | | | | **6** | | | **4** | | | | | **105** | | | | **41** | | **69** | | **2683** | | | |
|  | **0** | | **0** | | | **3** | | **3** | **0** | | **4** | | | **51** | | **54** | | **22** | **19** | **38** | **31** | | **1345** | **1338** | |
|  | | | | | | | | | | | | | | | | | | | | | | | | | |
| **12 MONTHS** | **2** | | | | | **5** | | | **5** | | | | | **191** | | | | **38** | | **9** | | **2433** | | | |
|  | **2** | | | **0** | | **2** | | **3** | **3** | | **2** | | | **97** | | **94** | | **19** | **19** | **3** | **6** | | **1219** | **1214** | |
|  | | | | | | | | | | | | | | | | | | | | | | | | | |
| **18 MONTHS** | **2** | | | | **12** | | | | **7** | | | **325** | | | | | | **23** | | **65** | | **1999** | | | |
|  | **0** | **2** | | | **5** | | **7** | | **3** | **4** | | **175** | | | **150** | | | **12** | **11** | **32** | **33** | | **992** | **1007** | |
|  | | | | | | | | | | | | | | | | | | | | | | | | | |
| **24 MONTHS** | **0** | | | | **8** | | | | **4** | | | | **448** | | | | | **9** | | **25** | | **1505** | | | |
|  | **0** | **0** | | | **6** | | **2** | | **1** | **3** | | | **223** | | **225** | | | **5** | **4** | **10** | **15** | | **747** | **758** | |
|  | | | | | | | | | | | | | | | | | | | | | | | | | |
| **30 MONTHS** | **0** | | | | **4** | | | | **6** | | | | | **397** | | | | **6** | | **45** | | **1047** | | | |
|  | **0** | **0** | | | **2** | | **2** | | **5** | | **1** | | | **192** | | **205** | | **3** | **3** | **24** | **21** | | **521** | **526** | |
|  | | | | | | | | | | | | | | | | | | | | | | | | | |
| **36 MONTHS** | **1** | | | | **5** | | | | **3** | | | | | **314** | | | | **1** | | **20** | | **703** | | | |
|  | **0** | **1** | | | **2** | | **3** | | **2** | | **1** | | | **155** | | **159** | | **1** | **0** | **10** | **10** | | **351** | **352** | |
|  | | | | | | | | | | | | | | | | | | | | | | | | | |
| **42 MONTHS** | **0** | | | | **4** | | | | **2** | | | | | **303** | | | **1** | | | **27** | | **366** | | | |
|  | **0** | **0** | | | **0** | | **4** | | **1** | | **1** | | | **164** | | **139** | | **1** | **0** | **15** | **12** | | **170** | **196** | |
|  | | | | | | | | | | | | | | | | | | | | | | | | | |
| **48 MONTHS** | **0** | | | | **2** | | | | **2** | | | | | **213** | | | | **0** | | **4** | | **145** | | | |
|  | **0** | **0** | | | **0** | | **2** | | **1** | | **1** | | | **97** | | **116** | | **0** | **0** | **4** | **0** | | **68** | **77** | |
|  | | | | | | | | | | | | | | | | | | | | | | | | | |
| **54 MONTHS** | **2** | | | | **0** | | | | **0** | | | | | **103** | | | | **0** | | **17** | | **23** | | | |
|  | **2** | **0** | | | **0** | | **0** | | **0** | | **0** | | | **48** | | **55** | | **0** | **0** | **9** | **8** | | **9** | **14** | |
|  | | | | | | | | | | | | | | | | | | | | | | | | | |
| **60 MONTHS** | **0** | | | | **0** | | | | **0** | | | | | **23** | | | | **0** | | **0** | | **0** | | | |
|  | **0** | **0** | | | **0** | | **0** | | **0** | | **0** | | | **9** | | **14** | | **0** | **0** | **0** | **0** | | **0** | **0** | |
|  | | | | | | | | | | | | | | | | | | | | | | | | | |
| **TOTALS** | **7** | | | | **53** | | | | **34** | | | | | **2422** | | | | **148** | | **338** | |  | | | |
|  | **4** | **3** | | | **20** | | **33** | | **16** | | **18** | | | **1211** | | **1211** | | **80** | **68** | **173** | **165** | |  | |  |

**Supplementary_Table_2: Additional baseline characteristics of the patients randomised in the active and placebo arms**

|  | **Atorvastatin 40mg (n=1504)** | | **Placebo (n=1498)** | |
| --- | --- | --- | --- | --- |
|  | N* | Count (%), mean (SD) or median (quartiles) | N* | Count (%), mean (SD) or median (quartiles) |
| **Demographic/Anthropometric characteristics** | | | | |
| Race | 1421 |  | 1430 |  |
| White |  | 1394 (98·1%) |  | 1407 (98·4%) |
| Asian/Asian British |  | 7 (0·5%) |  | 15 (1·0%) |
| Other Mixed |  | 11 (0·8%) |  | 2 (0·1%) |
| Black/Black British |  | 8 (0·6%) |  | 4 (0·3%) |
| **RA characteristics** | | | | |
| Tender joint count | 1490 | 3 (1-8) | 1489 | 3 (0-8) |
| Swollen joint count | 1488 | 2 (0-5) | 1490 | 2 (0-6) |
| Early morning stiffness (minutes) | 1275 | 30 (5-60) | 1263 | 30 (5-60) |
| DAS28 category | 1471 |  | 1471 |  |
| High |  | 258 (17·5%) |  | 233 (15·8%) |
| Moderate |  | 643 (43·7%) |  | 621 (42·2%) |
| Low |  | 199 (13·5%) |  | 218 (14·8%) |
| Remission |  | 371 (25·2%) |  | 399 (27·1%) |
| Pain score | 1349 | 39 (18-60) | 1333 | 35 (15-60) |
| Fatigue score | 1349 | 50 (22-73) | 1333 | 50 (21-72) |
| Wellness score | 1488 | 31 (15-53) | 1483 | 30 (14-52) |
| EQ5D VAS | 1461 | 70 (50-82) | 1458 | 70 (54-85) |
| HAQ DI score | 1473 | 1·25 (0·50-1·88) | 1464 | 1·25 (0·38-1·88) |
| **Cardiovascular characteristics** | | | | |
| Systolic blood pressure (1^st^ reading) | 1476 | 136 (18) | 1469 | 135 (18) |
| Systolic blood pressure (2^nd^ reading) | 1320 | 135 (17) | 1311 | 134 (18) |
| Diastolic blood pressure (1^st^ reading) | 1449 | 79 (10) | 1443 | 80 (10) |
| Diastolic blood pressure (2^nd^ reading) | 1291 | 79 (10) | 1282 | 79 (10) |
| Family history of diabetes | 1473 | 316 (21·5%) | 1471 | 327 (22·2%) |

* number with information available

**Supplementary_Table_3: Compliance (3a) and non-study statin use (3b) during the follow-up period**

**3a. Compliance**

| **Months of follow-up** |  | **Atorvastatin** |  |  | **Placebo** |  |
| --- | --- | --- | --- | --- | --- | --- |
|  | **N*** | **Compliant** | **Percentage** | **N*** | **Compliant** | **Percentage** |
| 3 | 1504 | 1332 | 89 | 1491 | 1332 | 89 |
| 6 | 1480 | 1158 | 78 | 1460 | 1162 | 80 |
| 12 | 1407 | 1004 | 71 | 1396 | 1001 | 72 |
| 24 | 1049 | 653 | 62 | 1065 | 627 | 59 |
| 36 | 583 | 311 | 53 | 565 | 293 | 52 |
| 48 | 204 | 92 | 45 | 205 | 86 | 42 |
| 60 | 18 | 7 | 39 | 8 | 2 | 25 |

**3b. Non-study statin use**

| **Months of follow-up** |  | **Atorvastatin** |  |  | **Placebo** |  |
| --- | --- | --- | --- | --- | --- | --- |
|  | **N*** | **Non-study statin** | **Percentage** | **N*** | **Non-study statin** | **Percentage** |
| 3 | 1504 | 8 | 0·5 | 1491 | 10 | 0·7 |
| 6 | 1480 | 8 | 0·5 | 1460 | 12 | 0·8 |
| 12 | 1407 | 15 | 1·1 | 1396 | 29 | 2·1 |
| 24 | 1049 | 18 | 1·7 | 1065 | 44 | 4·1 |
| 36 | 583 | 17 | 2·9 | 565 | 40 | 7·1 |
| 48 | 204 | 8 | 3·9 | 205 | 16 | 7·8 |
| 60 | 18 | 1 | 5·6 | 8 | 0 | 0·0 |

* number with information available

**Supplementary_Table_4: (4a) Secondary endpoint (components of the primary endpoint separately); (4b) Tertiary endpoint (deaths from any cause) in the atorvastatin and placebo groups**

**Table 4a: Patients with CV events**

|  | **Atorvastatin 40mg (n=1504)** | | **Placebo**  **(n=1498)** | |  |  |  |
| --- | --- | --- | --- | --- | --- | --- | --- |
| **Type** | **n** | **% (of group)** | **n** | **% (of group)** | **Hazard Ratio** | **Lower limit of 95% CI** | **Upper limit of 95% CI** |
| Coronary events [i·e· non-fatal myocardial infarction, coronary death or coronary revascularisation] | 13 | 0·9 | 23 | 1·5 | 1·79 | 0·91 | 3·54 |
| Non-fatal myocardial infarction | 11 | 0·7 | 20 | 1·3 | 1·84 | 0·88 | 3·84 |
| Coronary death | 2 | 0·1 | 2 | 0·1 | 1·00 | 0·14 | 7·11 |
| Coronary revascularisation | 8 | 0·5 | 14 | 0·9 | 1·77 | 0·74 | 4·23 |
| Presumed ischemic stroke or transient ischemic attack | 6 | 0·4 | 12 | 0·8 | 2·01 | 0·75 | 5·35 |
| Presumed ischemic stroke | 2 | 0·1 | 7 | 0·5 | 3·52 | 0·73 | 16·93 |
| Transient ischemic attack | 4 | 0·3 | 5 | 0·3 | 1·25 | 0·34 | 4·66 |
| Any non-coronary arterial revascularisation | 3 | 0·2 | 1 | 0·1 | 0·33 | 0·03 | 3·20 |
| Any other cardiovascular death excluding both confirmed cerebral haemorrhage [ICD I64-99 in the International Classification of Diseases 10^th^ revision]) and non-coronary cardiac death [ICD I00-I15 and I26-I52] | 0 | 0·0 | 0 | 0·0 |  |  |  |
| Peripheral atherosclerotic event | 1 | 0·1 | 0 | 0·0 |  |  |  |
| Suspect coronary heart disease death | 2 | 0·1 | 1 | 0·1 | 0·53 | 0·05 | 5·82 |
| **Patients with any of the above** | **24** | **1·6** | **36** | **2·4** |  |  |  |

**Table 4b: Deaths**

|  | **Atorvastatin 40mg (n=1504)** | | **Placebo (n=1498)** | |
| --- | --- | --- | --- | --- |
| Cause of death | n | % (of group) | n | % (of group) |
| Coronary | 4 | 0·3 | 3 | 0·2 |
| Other vascular | 2 | 0·1 | 3 | 0·2 |
| Non-vascular | 19 | 1·3 | 21 | 1·4 |
| **Any** | **25** | **1·7** | **27** | **1·8** |

CV: cardiovascular.

**Supplementary_Table_5: Hospitalizations in the Atorvastatin and Placebo groups**

|  | **Atorvastatin 40mg (n=1504)** | | **Placebo (n=1498)** | |
| --- | --- | --- | --- | --- |
| **Number of hospitalizations per patient** | **n** | **% (of group)** | **n** | **% (of group)** |
| 0 | 1290 | 85·8 | 1275 | 85·1 |
| 1 | 143 | 9·5 | 168 | 11·2 |
| 2 | 53 | 3·5 | 40 | 2·7 |
| 3 | 11 | 0·7 | 10 | 0·7 |
| 4 | 4 | 0·3 | 1 | 0·1 |
| 5 | 1 | 0·1 | 2 | 0·1 |
| 6 | 1 | 0·1 | 1 | 0·1 |
| 7 | 1 | 0·1 | 1 | 0·1 |
| 1 or more | 214 | 14·2 | 223 | 14·9 |

The p value for the comparison of the number of hospitalizations per patient is 0·710 (using Kendall’s tau-b). The p value for the comparison of the proportions of patients with at least one hospitalization is 0·641 (Fisher’s exact test).

**Anti-rheumatic drug therapy at baseline, during the trial and at end of trial.**

**PREDNISOLONE USE**

|  | Atorvastatin 40mg | | Placebo | | p value* |  |
| --- | --- | --- | --- | --- | --- | --- |
|  | n | Prednisolone daily dose | n | Prednisolone daily dose |  |  |
| Baseline | 204 | 5.0 (4.0-7.5) | 202 | 5.0 (3.9-7.5) | 0.917 |  |
| 3 months | 163 | 5.0 (3.0-7.5) | 168 | 5.0 (3.8-7.5) | 0.674 |  |
| 1 year | 75 | 5.0 (2.5-7.5) | 89 | 5.0 (4.8-7.5) | 0.328 |  |
| 2 years | 18 | 5.0 (5.0-7.1) | 23 | 5.0 (3.2-7.5) | 0.399 |  |

**from Mann-Whitney test*

**DMARD USE**

Each drug has four columns in the following order: Atorvastatin arm, start of trial; Atorvastatin arm, end of trial; Placebo arm, start of trial; Placebo arm, end of trial

**DMARD USE (START AND END OF STUDY)**

1. If ALT and AST are both available in a local hospital then ALT is to be preferred for liver monitoring since it is more liver specific than AST.

   Follow-up visits for TRACE RA DAS participants

   - Month 6 from first administration of trial drug (TRACE RA DAS sub-study only)

   Patient Global Assessment (VAS)

   Nurse Global Assessment (VAS)

   28 tender and swollen joint count ESR and/or CRP

   ^4 x 2.5mls blood sample (for RNA analysis) [↑](#footnote-ref-1)
2. overall event rate in the two groups combined [↑](#footnote-ref-2)
3. serum and plasma sample for TRACE RA BioBank is not required if patient is enrolled in TRACE RA DAS of 62 [↑](#footnote-ref-3)
